# Supplementary material for: Artificial intelligence as a diagnostic aid in cross-sectional radiological imaging of surgical pathology in the abdominopelvic cavity: a systematic review
Source: BMJ Open. 2023 Mar 6;13(3):e064739. doi: 10.1136/bmjopen-2022-064739 (PMC9990659; doi:10.1136/bmjopen-2022-064739)
Supplement: Supplementary data [file bmjopen-2022-064739supp002.pdf]

Online supplementary Table 1 Performance of AI models

First author (Reference)	Study aim	Best AI model	Output measures	AI performance	Human comparator	Human performance	External validation
**Acar [25]**	Distinguish the metastatic and completely responded areas regarding sclerotic bone lesions imaged via 68Ga-PSMA PET/ CT in patients with known bone metastasis.	KNN	Sensitivity<br>Specificity<br>Predictive values (PPV, NPV)<br>AUC<br>Overall accuracy	73.5%<br>73.7%<br>86.9%, 53.8%<br>76.0%<br>73.5%	NA	NA	No
**Coy [26]**	Investigate the diagnostic value and feasibility of a deep learning-based renal lesion classifier in differentiating ccRCC from ONC on routine four-phase MDCT in patients with pathologically confirmed renal masses.	CNN	Sensitivity<br>Specificity<br>Predictive values (PPV, NPV)<br>Overall accuracy	88.3%<br>52.9%<br>79.6%, 59.5%<br>75.4%	NA	NA	No
**Han [32]**	Develop reproducible and generalizable models for discriminating three major subtypes of RCCs using CT image analysis along with a ML algorithm.	CNN	Sensitivity<br>Specificity<br>AUC<br>Overall accuracy	0.98<br>0.93<br>0.9<br>0.85	NA	NA	No
**Koizumi [34]**	Investigate the diagnostic performance of computer-assisted diagnostic system for bone scintigraphy in prostate cancer patients with and without skeletal metastasis.	ANN	Sensitivity<br>Specificity<br>AUC	82.0%<br>83.0%<br>0.89	NA	NA	No
**Lee [35]**	Evaluate the performance of deep learning classifiers in discriminating normal and abnormal 18F-FACBC PET scans based on the presence of tumour recurrence and/or metastases in patients with	CNN	Sensitivity<br>Specificity<br>AUC	90.7%<br>95.1%<br>0.97	NA	NA	No

	prostate cancer and biochemical recurrence.						
**Oberai [39]**	Classify lipid poor enhancing renal masses into 'benign' and 'malignant' categories.	CNN	Sensitivity<br>Specificity<br>AUC<br>Overall accuracy	70.0%<br>81.0%<br>0.82<br>78.0%	NA	NA	No
**Lu [36]**	To develop an assistive tool to aid radiologists to more effectively and accurately determine lymph node metastases.	CNN	Sensitivity<br>Specificity<br>AUC<br>Overall accuracy	Not reported<br>Not reported<br>0.91<br>99.9%	Radiologists (number not specified)	Values not explicitly reported.	Yes
**Nayak [38]**	Study proposes a computer-aided diagnosis system for detecting cirrhosis and HCC.	SVM	Prediction accuracy	80.8 - 86.9%	NA	NA	No
**Sethi [28]**	Paper proposed automated CAD system for classification of abdomen diseases like tumour, cyst, stone and normal tissues from CT images of abdomen.	ANN	Sensitivity<br>Specificity<br>Predictive values (PPV, NPV)<br>AUC<br>Overall accuracy	95.0%<br>98.0%<br>No raw data<br>No raw data<br>95.1%	NA	NA	No
**Yasaka [29]**	Investigate diagnostic performance by using a deep learning method with a CNN for the differentiation of liver masses at dynamic contrast agent-enhanced CT.	CNN	Sensitivity<br>Specificity<br>AUC<br>Overall accuracy	0.33 - 1.00<br>Not reported<br>0.92<br>0.84	NA	NA	No
**Yuan [30]**	Develop a CNN-based ResNet3d algorithm and SVM classified to detect synchronous peritoneal carcinomatosis in colorectal cancer.	CNN + SVM combination	Sensitivity<br>Specificity<br>Predictive values (PPV, NPV)<br>AUC<br>Overall accuracy	93.8%<br>94.4%<br>93.8%, 94.4%<br>0.92<br>94.1%	2 Radiologists (senior and junior)	57.4%, 58.1%<br>100%, 98%<br>No raw data<br>0.79, 0.78<br>No raw data	Yes
**Zhao [31]**	Develop and validate a deep-learning-based, fully automated lymph node detection and segmentation model based on mpMRI.	CNN	Sensitivity<br><br>Predictive values (PPV, NPV)	80.0%<br><br>73.5%, NA	4 Radiologists	38.2% (Int), 62.6% (Ext)<br>43.8% (Int), 35.9% (Ext)	Yes
**Saiprasad [27]**	Identify adrenal glands from a volumetric CT acquisition and	RFC	Sensitivity<br>Specificity	80.0%<br>90.0%	NA	NA	No

	determine whether they are normal or abnormal.						
**Nakagawa [37]**	Compare the performance of ML using mp-MRI and PET to distinguish between uterine sarcoma and leiomyoma.	Logistic regression (multivariate)	AUC	0.92	2 Radiologists (11 and 20 years of experience)	0.97 and 0.89	Yes
**Kawauchi [33]**	Develop a CNN-based system that can classify whole-body FDG PET as 1) benign, 2) malignant or 3) equivocal.	CNN	Prediction	91.0% - 94.9% (abdomen) 95.3% - 99.7% (pelvic region)	NA	NA	No

Abbreviations: Artificial neural network (ANN), area under the curve (AUC), benign oncocytoma (ONC), clear cell renal cell carcinoma (ccRCC), CNN (convolutional neural network), computer tomography (CT), Ext (external data set), hepatocellular carcinoma (HCC), Int (internal data set), machine learning (ML), k-nearest neighbours algorithm (KNN), multiparametric magnetic resonance imaging (mpMRI), negative predictive value (NPV), positive predictive value (PPV), positron emission tomography (PET), random forest classification (RFC) and support vector machine (SVM).

.
