# Supplementary material for: Artificial intelligence as a diagnostic aid in cross-sectional radiological imaging of surgical pathology in the abdominopelvic cavity: a systematic review
Source: BMJ Open. 2023 Mar 6;13(3):e064739. doi: 10.1136/bmjopen-2022-064739 (PMC9990659; doi:10.1136/bmjopen-2022-064739)
Supplement: Author's manuscript [file bmjopen-2022-064739.draft_revisions.pdf]

BMJ Open

BMJ Open is committed to open peer review. As part of this commitment we make the peer review history of every article we publish publicly available.

When an article is published we post the peer reviewers' comments and the authors' responses online. We also post the versions of the paper that were used during peer review. These are the versions that the peer review comments apply to.

The versions of the paper that follow are the versions that were submitted during the peer review process. They are not the versions of record or the final published versions. They should not be cited or distributed as the published version of this manuscript.

BMJ Open is an open access journal and the full, final, typeset and author-corrected version of record of the manuscript is available on our site with no access controls, subscription charges or pay-per-view fees (http://bmjopen.bmj.com).

If you have any questions on BMJ Open's open peer review process please email info.bmjopen@bmj.com

BMJ Open

Artificial intelligence as a diagnostic aid in cross-sectional radiological imaging of surgical pathology in the abdominopelvic cavity: A systematic review

Journal:	*BMJ Open*
Manuscript ID	bmjopen-2022-064739
Article Type:	Original research
Date Submitted by the Author:	20-May-2022
Complete List of Authors:	Fowler, George; NIHR Bristol Biomedical Research Centre, Surgical Innovation Theme, University of Bristol<br>Blencowe, Natalie; NIHR Bristol Biomedical Research Centre, Surgical Innovation Theme, University of Bristol<br>Hardacre, Conor; Bristol Medical School<br>Callaway, Mark; Bristol Royal Infirmary, Department of Clinical Radiology<br>Smart , Neil ; Royal Devon and Exeter NHS Foundation Trust, Exeter Surgical Health Services Research Unit (HeSRU)<br>Macefield, Rhiannon; NIHR Bristol Biomedical Research Centre, Surgical Innovation Theme, University of Bristol
Keywords:	SURGERY, Diagnostic radiology < RADIOLOGY & IMAGING, Adult surgery < SURGERY

SCHOLARONE™
Manuscripts

I, the Submitting Author has the right to grant and does grant on behalf of all authors of the Work (as defined in the below author licence), an exclusive licence and/or a non-exclusive licence for contributions from authors who are: i) UK Crown employees; ii) where BMJ has agreed a CC-BY licence shall apply, and/or iii) in accordance with the terms applicable for US Federal Government officers or employees acting as part of their official duties; on a worldwide, perpetual, irrevocable, royalty-free basis to BMJ Publishing Group Ltd ("BMJ") its licensees and where the relevant Journal is co-owned by BMJ to the co-owners of the Journal, to publish the Work in this journal and any other BMJ products and to exploit all rights, as set out in our licence.

The Submitting Author accepts and understands that any supply made under these terms is made by BMJ to the Submitting Author unless you are acting as an employee on behalf of your employer or a postgraduate student of an affiliated institution which is paying any applicable article publishing charge ("APC") for Open Access articles. Where the Submitting Author wishes to make the Work available on an Open Access basis (and intends to pay the relevant APC), the terms of reuse of such Open Access shall be governed by a Creative Commons licence – details of these licences and which Creative Commons licence will apply to this Work are set out in our licence referred to above.

Other than as permitted in any relevant BMJ Author's Self Archiving Policies, I confirm this Work has not been accepted for publication elsewhere, is not being considered for publication elsewhere and does not duplicate material already published. I confirm all authors consent to publication of this Work and authorise the granting of this licence.

For peer review only

Artificial intelligence as a diagnostic aid in cross-sectional radiological imaging of surgical pathology in the abdominopelvic cavity: A systematic review

Authors: George E Fowler[1]
george.fowler@bristol.ac.uk

Natalie S Blencowe[1]
natalie.blencowe@bristol.ac.uk

Conor Hardacre[2]
ch17008@bristol.ac.uk

Mark P Callaway[3]
mark.callaway@uhbw.nhs.uk

Neil J Smart[4]
drneilsmart@hotmail.com

Rhiannon C Macefield[1]
r.macefield@bristol.ac.uk

Corresponding author: George E Fowler
NIHR Bristol Biomedical Research Centre. Surgical Innovation Theme, University of Bristol, UK.

Institutions: [1]NIHR Bristol Biomedical Research Centre. Surgical Innovation Theme, University of Bristol, UK.
[2]Bristol Medical School, University of Bristol, UK.
[3]Department of Clinical Radiology, University Hospital Bristol and Weston NHS Foundation Trust, Bristol, UK.
[4]Exeter Surgical Health Services Research Unit (HeSRU), Royal Devon and Exeter Hospital, Exeter, UK.

For peer review only

ORCID ID(s): GEF (0000-0002-4133-802X)
RCM (0000-0002-6606-5427)
CH (0000-0001-9800-0479)
MPC
NJS (0000-0002-3043-8324)
NSB (0000-0002-6111-2175)

Acknowledgements: The authors would like to thank Ms Catherine Borwick, Information Specialist, University of Bristol, for her input and expertise to develop the search strategy. The authors are grateful to Dr Christin Hoffmann (University of Bristol) for leading patient and public feedback sessions as part of the wider programme of this research and Dr Xiaoxuan Liu (University of Birmingham and University Hospitals Birmingham NHS Foundation Trust) for providing expert comments on the manuscript.

Category: Systematic review.

Key words: Artificial intelligence; deep learning; systematic review; surgery; diagnostic

Word count: 2,482 words

For peer review only

Abstract

Objectives

There is emerging use of artificial intelligence (AI) models to aid diagnostic imaging. This systematic review examines and critically appraises the application of AI models to identify surgical pathology from cross-sectional radiological images of the abdominopelvic cavity, to identify current limitations and inform future research.

Methods

Systematic database searches (Medline, EMBASE, Cochrane Central Register of Controlled Trials) were performed. Date limitations of January 2012 to July 2021 were applied. Study characteristics, descriptions of AI models and outcomes assessing diagnostic performance were extracted. A narrative synthesis was performed in accordance with the Synthesis Without Meta-analysis guidelines. Risk of bias was assessed (QUADAS-2).

Results

Fifteen retrospective studies from a range of surgical specialities were included. Studies were diverse in the intention of the AI applications and the models used. AI training and test sets comprised a median of 130 (range: 5–2440) and 37 (range: 10–1045) patients respectively. Diagnostic performance of models varied (range: 70-95% sensitivity, 53%–98% specificity). Only four studies compared the AI model with human performance. Reporting of studies was unstandardised and often lacking in detail. Most studies (n=14) were judged as having overall high risk of bias with concerns regarding applicability.

For peer review only

Conclusion

AI application in this field is diverse. Adherence to reporting guidelines is warranted. With finite healthcare resources, future endeavours may benefit from targeting areas where radiological expertise is in high demand to provide greater efficiency in clinical care. Translation to clinical practice and adoption of a multidisciplinary approach should be of high priority.

PROSPERO registration number: CRD42021237249.

For peer review only

BOX: Strengths and limitations of this study

- This systematic review evaluating the diagnostic performance of artificial intelligence models using cross-sectional radiological images of the abdominopelvic cavity, has identified current limitations and evidence gaps, and thereby focusing future research efforts.
- Robust methodology was undertaken including duplication screening, data extraction and of risk of bias assessment.
- Findings may be limited by including English language publications only.

For peer review only

Introduction

The widespread adoption of digital healthcare provides vast data to enable the application of artificial intelligence (AI) in pattern recognition [1]. This can alleviate the burden of, or replace, tasks traditionally dependent on clinicians. Examples include the interpretation of medical images for diagnostic, prognostic, surveillance, and management decisions, which otherwise rely on a limited number of interpreters and human resources [2]. There has been a surge of research into the use of AI in diagnostic imaging, exploring how it can support clinicians and provide greater efficacy and efficiency in clinical care [3].

Systematic reviews on the diagnostic accuracy of AI in medical imaging (including respiratory medicine, ophthalmology and breast cancer) [4], gastroenterology [5], neurosurgery [6] and vascular surgery [7] have demonstrated the diverse application of AI models to detect many pathologies. A variety of imaging modalities have been explored (e.g., computer tomography (CT), magnetic resonance imaging (MRI) and positron emission tomography (PET)). AI models can demonstrate diagnostic performances equivalent to that of experts [8] and with greater efficiency, for example, in the time taken to diagnose childhood cataracts (5.7 minutes quicker than senior consultants)[9]. However, while AI technologies offer to markedly reduce the clinical workload, "black box" techniques (AI algorithms) can be difficult or impossible to interpret, which can be a barrier to adopting these techniques into clinical practice. Furthermore, many AI studies are proof-of-concept [10] and poorly reported [11,12], including limited details on participants, making it difficult to replicate or interpret the study findings [12].

For peer review only

A systematic review of the diagnostic accuracy of AI models in cross-sectional radiological imaging of the abdominopelvic cavity is lacking [13]. Synthesis of the current AI research in this area could benefit several different surgical specialities which image this region, such as endocrine surgery, gastrointestinal surgery, obstetrics and gynaecology, urology and vascular surgery to guide their clinical decision making. This study aimed to conduct a systematic review to examine and critically appraise the application of AI models to identify surgical pathology from cross-sectional radiological images of the abdominopelvic cavity, to identify current limitations and inform future research efforts.

For peer review only

METHODS

Protocol and registration

This systematic review was registered with the International Prospective Register of Systematic Reviews (PROSPERO: CRD42021237249). A study protocol has previously been published [13]. It is reported in accordance with the Preferred Reporting Items for Systematic Reviews and Meta-analysis of Diagnostic Test Accuracy Studies (PRISMA-DTA) [14].

Information sources

Electronic searches of OVID SP versions of Medline, EMBASE and the Cochrane Central Register of Controlled Trials (CENTRAL) databases were conducted to identify all potentially relevant studies. Date limitations between January 1st 2012 and July 31st 2021 were applied, to account for advancements in machine learning performance and the development of deep learning approaches since 2012, in line with existing reviews [15]. Reference lists of included articles were screened to identify further relevant studies.

Search strategy and study selection

A comprehensive search syntax was developed with guidance from an information specialist using text words and Medical Subject Headings (MeSH) related to three domains: “artificial intelligence”, “diagnostic imaging” and the “abdominopelvic cavity” (Appendix S1). Database search results were imported into reference management software (EndNote X9, Clarivate Analytics, USA) and duplicates removed.

For peer review only

Assessment of study eligibility was performed in two stages. First, titles and abstracts were screened for inclusion by two independent reviewers (GEF and CH). Any conflicts were resolved through discussion, referring to the wider study team if required. Final eligibility was assessed by a full-text review of potentially eligible studies by the same process. Management of the screening process was aided by Rayyan software (Rayyan Systems Inc., Cambridge, MA, USA) [16].

Eligibility criteria

Primary research studies were considered for eligibility using the PIRT (participants, index test(s), reference standard and target condition) framework [17]. Participants were adults with pathology within the abdominopelvic cavity diagnosed using CT, MRI or PET. Diagnostic endoscopy was excluded as existing reviews have explored the performance of AI models in this area [18,19]. The index test was studies considering AI models as an intervention with the aim to provide a diagnosis. The reference standard was 'standard practice' to allow for variation across the included studies. The target condition was abdominopelvic cavity pathology which has had, or may warrant, an invasive procedure [20] for therapeutic intent.

Excluded were secondary research studies (e.g., systematic reviews), case reports and case series, absence of full text (e.g., conference abstracts), animal studies and non-English articles.

Data extraction and management

Data extraction from the included articles was independently performed by two reviewers (GEF and CH). Data management software (REDCap version 9.5.23,

For peer review only

Vanderbilt University, USA) [21] and a pre-designed standardized form were used. Data were extracted under the following three subheadings:

(1) Study characteristics

Extracted data included the name of the first author, their affiliated country, composition of the study team (e.g., software engineers, radiologists, and surgeons who routinely operate on surgical pathology within the abdominopelvic cavity (e.g., gastrointestinal surgeons, urologists and gynaecologists)), year of publication, study aim and design (i.e., 'prospective' or 'retrospective'), surgical subspeciality and pathology studied (i.e., benign, malignant tumours, multiple or other). Information on the reporting of ethics and/or regulatory approval (e.g., Medicines and Healthcare products Regulatory Agency), patient and publication involvement and authors' mention of using a reporting guideline (e.g., Standards for Reporting of Diagnostic Accuracy Studies) was recorded.

(2) Training data

Extracted data on the input features (data used to develop the AI model) included the modality of cross-sectional radiological imaging (CT, PET/CT, MRI, PET and other), the AI model used, the reference standard and the reporting and size of the training and test sets.

(3) Outcomes

The performance of the AI models and human comparator (where applicable) were extracted. Diagnostic measures of accuracy included reported sensitivity, specificity, positive predictive values and the area under the receiver operating characteristic

For peer review only

curve (AUC). The interpretation time (seconds) for studies comparing the performance of the AI model with a human comparator (where reported) were extracted.

Risk of bias and applicability

Risk of bias was assessed independently by two reviewers (GEF and RCM) using the QUADAS-2 tool [22]. A version of the QUADAS-2 tool for AI studies [23] was still in development and not available at the time of conducting the current review. The generic QUADAS-2 tool with the pre-existing modified signalling questions was used to assess four domains including patient selection, index test, reference standard and flow and timing [22]. An overall judgement of "at risk of bias" or "concerns regarding applicability" was assigned if more one or more domains were judged as "high" or "unclear" [22]. Judgements of applicability assessed whether the study matched the review question [22].

Data Synthesis

A narrative synthesis was conducted according to the Synthesis Without Meta-analysis guidelines [24]. The synthesis was planned to focus on the primary outcome with studies grouped by the modality of radiological imaging, surgical subspeciality and pathology studied, as outlined in the protocol [13]. A broader approach was, however, adopted due to the small number of included studies and their heterogeneity. A meta-analysis was not performed due to the broad nature of the included studies.

Patient and Public Involvement

As part of the wider programme of work (Bristol Biomedical Research Centre, National Institute for Health Research (NIHR) Bristol BRC), patients and the public were

For peer review only

consulted on their views of AI being used to guide doctors to make decisions about treatment. Overall, it was perceived positively, and they were supportive of its adoption in healthcare.

For peer review only

RESULTS

Database searching identified 628 records, with a further five studies identified through reference lists of included articles. After the removal of duplicates, 580 were screened and, 52 full-text articles were assessed for eligibility. Fifteen studies were finally included (Figure 1) [25–39].

Study characteristics

Characteristics of the included studies and details of the AI models are summarised (Tables 1 and 2). All were retrospective studies conducted in six different countries: Japan (n=4), USA (n=4), China (n=3), India (n=2), Turkey (n=1) and South Korea (n=1). Studies were all proof-of-concept (i.e., not applied in a clinical setting;), from four surgical specialities: urology (n=6), gastrointestinal surgery (n=6), endocrine surgery (n=1) and gynaecology (n=1). One study was not specific to a single speciality and involved whole-body PET/CT across four anatomical regions (head-and-neck, chest, abdomen, and pelvis)[33]. Most studies focused on malignant tumours (n=11) using CT as the imaging modality (n=8). Most studies (n=14) included an ethical approval statement. No studies, however, mentioned patient and public involvement or the use of a reporting guideline.

For peer review only

Table 1 Characteristics of the included studies

Characteristics	Number of studies n=15
**Country of origin**	
Japan	4
USA	4
China	3
India	2
Turkey	1
South Korea	1
**Year of publication**	
2020	5
2019	5
2018	2
2017	1
2016	1
2013	1
**Surgical subspecialty**	
Urology	6
Gastrointestinal	6
Endocrine	1
Gynaecology	1
Not reported	1
**Pathology studied**	
Malignant tumours	11
Multiple pathologies	2
Other	2
**Modality of radiological imaging**	
CT	8
PET / CT	2
MRI	2
PET	1
MRI and PET / CT	1
Other	1

Abbreviations: CT (computer tomography), PET (positron emission tomography) and MRI (magnetic resonance imaging).

For peer review only

Table 2 Study details and AI models

First author (Reference)	Country of origin	Surgical speciality	Pathology studied	Modality of radiological imaging	AI models	Reference standard	Size of training set (patients)	Size of test set (patients)
**Acar [25]**	Turkey	Urology	Malignancy	PET/CT	KNN	2 Nuclear Medicine Physicians	"90% [total 75 patients]"	"10% [total 75 patients]"
**Coy [26]**	USA	Urology	Malignancy	CT	CNN	Pathologist (number not specified)	90% [total 179 patients]	10% [total 179 patients]
**Han [32]**	South Korea	Urology	Malignancy	CT	CNN	Pathologist (number not specified)	135	34
**Koizumi [34]**	Japan	Urology	Malignancy	Other (bone scintigraphy)	ANN	2 Radiology Nuclear Physicians	Not reported	226
**Lee [35]**	USA	Urology	Malignancy	PET	CNN	1 Imaging Physicians	Not reported	Not reported
**Oberai [39]**	USA	Urology	Malignancy	CT	CNN	1 Genitourinary Pathologists	120	23
**Lu [36]**	China	Gastrointestinal	Malignancy	MRI	CNN	Radiologists (number not specified)	Not reported	Not reported (+ 414 validation test set)
**Nayak [38]**	India	Gastrointestinal	Multiple (cirrhosis, hepatocellular carcinoma)	CT	SVM	1 Radiologist	40	Not reported
**Sethi [28]**	India	Gastrointestinal	Multiple (normal, cystic, calculus or tumour tissues)	CT	ANN, SVM	Unclear	Not reported	Not reported
**Yasaka [29]**	Japan	Gastrointestinal	Malignancy	CT	CNN	Radiologists (number not specified)	460	100

For peer review only - http://bmjopen.bmj.com/site/about/guidelines.xhtml

**Yuan [30]**	China	Gastrointestinal	Malignancy	CT	SVM, CNN	1 Pathologist	130	40
**Zhao [31]**	China	Gastrointestinal	Malignancy	MRI	CNN	3 Radiologists	293	81
**Saiprasad [27]**	USA	Endocrine Surgery	Other (normal vs abnormal)	CT	RFC	2 Radiologists	5	10
**Nakagawa [37]**	Japan	Gynaecology	Malignancy	MRI and PET/CT	Logistic regression (univariate and multivariate)	2 Radiologists and pathologists (number not specified)	66	Unclear
**Kawauchi, [33]**	Japan	Uncharacterized	Other (benign/malignant /equivocal)	PET/CT	CNN	1 Nuclear Medicine Physician	2440	1045

Abbreviations: Computerized tomography (CT), convolutional neural network (CNN), k-nearest neighbours algorithm (KNN), magnetic resonance imaging (MRI), positron emission tomography (PET), random forest classification (RFC) and support vector machine (SVM).

For peer review only

The composition and expertise within each study team varied. Three studies (n=3) comprised teams including software engineers, radiologists and surgeons. A small number of studies were comprised of only radiologists (n=2) or software engineers alone (n=1). Four study teams were comprised software engineers and radiologists together. Of the remaining five studies, three had no apparent radiological team and two were comprised of radiologists with either a team of software engineers, surgeons of physicians.

Training data

AI training and test sets within the included studies comprised a median of 130 (range: 5 – 2440) and 37 (range: 10 – 1045) patients, respectively (Table 2). This information, however, was not always available (n=6 studies). There was variability in the reference standard, including the number of clinicians (range: 1–3) for either a radiological (n=9) or histological (n=4) diagnosis or both (n=1). Only one study had an unclear reference standard (Table 2).

Outcomes

The intent of the AI applications in the included studies varied, with the majority focusing on diagnosing advanced or recurrent cancer (n=6 studies) and four studies classifying the pathology (i.e., normal or abnormal, or benign or malignant) (online supplementary Table 1). Diagnostic performance of the AI models ranged between 70.0 and 95.0% sensitivity, and 52.9% and 98.0% specificity (online supplementary Table 1). The reporting of the diagnostic measures of accuracy were unstandardised, for example, there was different output measures across the studies and three studies did not report all of their outcome measures (online supplementary Table 1).

For peer review only

Following model development with a training and tuning set, four studies had an external validation test set and compared the performance of the AI model with a human comparator (a radiologist) (online supplementary Table 1) [30,31,36,37]. The diagnostic performance of the radiologists ranged between 57.4% and 62.8% sensitivity and 0.89 and 0.97 AUC (online supplementary Table 1). In these studies, there was variability in both the number of patients (range 50–414; from one to six different centres) and number of radiologists (range 2–4) and their years of experience. These studies reported the diagnostic performance of the AI model as either superior (n=2; in rectal and advanced gastrointestinal cancer) [30,31] or comparable (n=2; in metastatic gastrointestinal pathology and a gynaecology study distinguishing between malignant tumours and benign pathology) to radiologists [36,37]. Two of these studies included a comparison of the interpretation time of the AI model with the radiologists [31,36]. AI models outperformed the radiologists in both studies (1–2 seconds vs 200 seconds per case, $p<0.05$ [31] and 20s vs 600s for an average of 100 MRI images [36]).

Risk of bias and applicability

With the exception of one study [31], all the included studies had an overall judgement of "at risk of bias" and "concerns regarding applicability" (Table 3). This was predominantly due to comparisons based on either a single clinician's assessment (n=10) or an unclear number of clinicians for the assessment (n=5), from either a small (e.g., 10 patients in one study) or unclear size of test set and most models were developed only from internal validations (n=11) (Table 2 and online supplementary Table 1).

For peer review only

Table 3 Study quality assessment (QUADAS-2 tool)

First author (reference)	RISK OF BIAS				APPLICABILITY CONCERNS		
	PATIENT SELECTION	INDEX TEST	REFERENCE STANDARD	FLOW AND TIMING	PATIENT SELECTION	INDEX TEST	REFERENCE STANDARD
Acar [25]	Low	Low	High	Low	Low	Low	High
Coy [26]	Low	Low	High	Low	Low	Low	High
Han [32]	Unclear	Low	High	Low	Low	Low	High
Kawauchi [33]	Low	Low	High	Low	Low	Low	High
Lu [36]	Unclear	Low	High	Low	Low	Low	High
Nakagawa [37]	Low	Low	High	Low	Low	Low	High
Koizumi [34]	Low	Low	High	Low	Low	Low	High
Lee [35]	Low	Low	High	Low	Low	Low	High
Nayak [38]	Unclear	Low	Unclear	Low	Low	Low	High
Oberai [39]	Low	Low	High	Low	Low	Low	High
Yasaka [29]	Unclear	Low	High	Low	Low	Low	High
Saiprasad [27]	Unclear	Low	High	Low	Low	Low	High
Sethi [28]	High	Low	Unclear	Low	Low	Low	Unclear
Yuan [30]	Low	Low	High	Low	Low	Low	High
Zhao [31]	Low	Low	Low	Low	Low	Low	Low

For peer review only

DISCUSSION

The major finding of this review was the heterogeneity in the AI applications across the included studies regardless of the surgical speciality or pathology. Early phase studies of AI innovation, particularly focusing on advanced or recurrent malignancy, were identified with promising diagnostic accuracies to support clinical decision making. Future AI research could benefit from targeting areas where radiological expertise is in high demand or the data is complex to interpret; for example, adrenal incidentalomas [40] and images from virtual colonoscopy [2]. Attention should also be directed to the governance of AI, particularly on where the responsibility lies if the AI model misses a lesion.

In this review, several reporting issues were identified, including for the reference standard and training data. Poor adherence to reporting guidelines is a common finding in the existing literature for diagnostic accuracy studies assessing AI interventions [4,11]. The Standards for Reporting of Diagnostic Accuracy-AI (STARD-AI) Steering Group are developing an AI-specific extension to the STARD statement, which aims to improve reporting of AI diagnostic accuracy studies [41]. This steering group highlighted three pitfalls, which are also reflected in this review: (1) unclear methodological interpretation (e.g. methods of validation and comparison to human performance), (2) unstandardised nomenclature (e.g. varying definition of the term 'validation'), and (3) heterogeneity of the outcome measures (e.g. sensitivity, specificity, predictive values and AUC) [42]. Endeavours to address this include the development of specific reporting guidelines for authors of AI studies, including protocols (SPIRIT-AI) [43], reports (CONSORT-AI) [44] and proposals (MINIMAR)

For peer review only

[45]. These efforts should improve both the reporting quality and make it easier to interpret and compare AI studies.

A minority of the included studies compared the diagnostic performance of the AI model with a clinician's diagnosis. These studies reported a faster and superior or equivalent diagnostic performance with the AI model. A recent review found only 51 studies worldwide reporting the implementation and evaluation of AI applications in clinical practice [46]. While many AI studies are currently retrospective and proof-of-concept [10,11,46], which may be appropriate for early phase surgical research, future efforts should evaluate the role of AI in a clinical setting. This should adopt a multidisciplinary team of all relevant stakeholders (e.g., software engineers, radiologists, and surgeons) to ensure the diverse and relevant skills sets can work together to produce both high-quality and clinically relevant AI research.

This review included a robust methodology, comprehensive search strategy and a multidisciplinary team. Some limitations, however, are acknowledged. Despite having a broad search strategy, relevant studies may have been missed by excluding articles that were not published in the English language. It did not encompass all diagnostic applications of AI in this region, such as diagnostic imaging for prognostic, surveillance, and management decisions meaning findings are not generalisable to these wider contexts. However, recommendations for prioritising future endeavours on clinical need, adhering to reporting guidelines and standardised and transparent reporting can be considered appropriate for all studies assessing AI interventions in healthcare.

For peer review only

This review identified a diverse application of AI innovation in this field. Most studies were proof-of-concept and more 'comparator' studies in the clinical setting are needed. Future AI research could build on existing studies with translation to clinical practice, adopting a multidisciplinary approach, including patient and public involvement, which was lacking in the studies of this review. This could target areas of clinical need and adherence to new and developing guidelines for reporting AI studies is also warranted.

For peer review only

ETHICS STATEMENT

Ethical approval was not required as no primary data were collected.

CONTRIBUTORSHIP STATEMENT

GEF and NSB conceived the idea for this systematic review. The search strategy was developed by GEF, RCM, CH, MPC, NJS and NSB. GEF and CH performed screening and data extraction. GEF and RCM conducted the QUADAS-2 assessments. All authors contributed to data analysis. GEF drafted the manuscript (guarantor of review), and it was critically appraised by RCM, CH, MPC, NJS and NSB. All authors approved the final manuscript before submission.

COMPETING INTERESTS

The authors have no conflicts of interest to declare.

FUNDING

NSB is funded by an MRC Clinical Scientist Award (grant number: MR/S001751/1). This study was supported by the NIHR Biomedical Research Centre at University Hospitals Bristol and Weston NHS Foundation Trust and the University of Bristol. The views expressed are those of the author(s) and not necessarily those of the NIHR or the Department of Health and Social Care.

DATA SHARING AGREEMENT

All data relevant to the study are included in the article or uploaded as supplementary information.

For peer review only

References

1 Oren O, Gersh BJ, Bhatt DL. Artificial intelligence in medical imaging: switching from radiographic pathological data to clinically meaningful endpoints. *Lancet Digit Heal* 2020;**2**:e486–8. doi:10.1016/S2589-7500(20)30160-6

2 Hosny A, Parmar C, Quackenbush J, *et al.* Artificial intelligence in radiology. *Nat Rev Cancer* 2018;**18**:500–10. doi:10.1038/s41568-018-0016-5.Artificial

3 He J, Baxter SL, Xu J, *et al.* The practical implementation of artificial intelligence technologies in medicine. *Nat Med* 2019;**25**:30–6. doi:10.1038/s41591-018-0307-0

4 Aggarwal R, Sounderajah V, Martin G, *et al.* Diagnostic accuracy of deep learning in medical imaging: a systematic review and meta-analysis. *npj Digit Med* 2021;**4**. doi:10.1038/s41746-021-00438-z

5 Jin P, Ji X, Kang W, *et al.* Artificial intelligence in gastric cancer: a systematic review. *J Cancer Res Clin Oncol* 2020;**146**:2339–50. doi:10.1007/s00432-020-03304-9

6 Senders JT, Arnaout O, Karhade A V., *et al.* Natural and artificial intelligence in neurosurgery: A systematic review. *Clin Neurosurg* 2018;**83**:181–92. doi:10.1093/neuros/nyx384

7 Raffort J, Adam C, Carrier M, *et al.* Artificial intelligence in abdominal aortic aneurysm. *J Vasc Surg* 2020;**72**:321-333.e1. doi:10.1016/j.jvs.2019.12.026

8 Esteva A, Kuprel B, Novoa RA, *et al.* Dermatologist-level classification of skin cancer with deep neural networks. *Nature* 2017;**542**:115–8. doi:10.1038/nature21056

9 Lin H, Li R, Liu Z, *et al.* Diagnostic Efficacy and Therapeutic Decision-making Capacity of an Artificial Intelligence Platform for Childhood Cataracts in Eye

For peer review only

Clinics: A Multicentre Randomized Controlled Trial. *EClinicalMedicine* 2019;**9**:52–9. doi:10.1016/j.eclinm.2019.03.001

10 Ben-Israel D, Jacobs WB, Casha S, *et al.* The impact of machine learning on patient care: A systematic review. *Artif Intell Med* 2020;**103**:101785. doi:10.1016/j.artmed.2019.101785

11 Nagendran M, Chen Y, Lovejoy CA, *et al.* Artificial intelligence versus clinicians: Systematic review of design, reporting standards, and claims of deep learning studies in medical imaging. *BMJ* 2020;**368**:1–12. doi:10.1136/bmj.m689

12 Yusuf M, Atal I, Li J, *et al.* Reporting quality of studies using machine learning models for medical diagnosis: a systematic review. *BMJ Open* 2020;**10**:e034568. doi:10.1136/bmjopen-2019-034568

13 Fowler GE, Macefield RC, Hardacre C, *et al.* Artificial intelligence as a diagnostic aid in cross-sectional radiological imaging of the abdominopelvic cavity: a protocol for a systematic review. *BMJ Open* 2021;**11**:e054411. doi:10.1136/bmjopen-2021-054411

14 McInnes MDF, Moher D, Thombs BD, *et al.* Preferred Reporting Items for a Systematic Review and Meta-analysis of Diagnostic Test Accuracy Studies The PRISMA-DTA Statement. *JAMA - J Am Med Assoc* 2018;**319**:388–96. doi:10.1001/jama.2017.19163

15 Liu X, Faes L, Kale AU, *et al.* A comparison of deep learning performance against health-care professionals in detecting diseases from medical imaging: a systematic review and meta-analysis. *Lancet Digit Heal* 2019;**1**:e271–97. doi:10.1016/S2589-7500(19)30123-2

16 Ouzzani M, Hammady H, Fedorowicz Z, *et al.* Rayyan-a web and mobile app for systematic reviews. *Syst Rev* 2016;**5**:1–10. doi:10.1186/s13643-016-0384-4

For peer review only

17 JJ, Deeks, S, Wisniewski DC, Diagnostic C, Accuracy T, *et al.* Guide to the contents of a Cochrane Diagnostic Test Accuracy Protocol. *Cochrane Handb Syst Rev Diagnostic Test Accuracy Version 100 Cochrane Collab* 2013;:1–15.http://srdta.cochrane.org/.

18 Hassan C, Spadaccini M, Iannone A, *et al.* Performance of artificial intelligence for colonoscopy regarding adenoma and polyp detection: a meta-analysis. *Gastrointest Endosc* Published Online First: 2020. doi:10.1016/j.gie.2020.06.059

19 Lui TKL, Tsui VWM, Leung WK. Accuracy of artificial intelligence–assisted detection of upper GI lesions: a systematic review and meta-analysis. *Gastrointest Endosc* 2020;**92**:821-830.e9. doi:10.1016/j.gie.2020.06.034

20 Cousins S, Blencowe NS, Blazeby JM. What is an invasive procedure? A definition to inform study design, evidence synthesis and research tracking. *BMJ Open* 2019;**9**:2018–20. doi:10.1136/bmjopen-2018-028576

21 Harris PA, Taylor R, Minor BL, *et al.* The REDCap consortium: Building an international community of software platform partners. *J Biomed Inform* 2019;**95**:103208. doi:10.1016/j.jbi.2019.103208

22 Whiting PF, Rutjes AW, Westwood ME, Mallett S, Deeks JJ, Reitsma JB, Leeflang MM, Sterne JA BP. QUADAS-2 Group. QUADAS-2: a revised tool for the quality assessment of diagnostic accuracy studies. *Ann Intern Med* 2011;**8**:29–36. doi:0.7326/0003-4819-155-8-201110180-00009. PMID: 22007046.

23 Viknesh Sounderajah, Hutan Ashrafian, Sherri Rose, Nigam H. Shah, Marzyeh Ghassemi, Robert Golub, Charles E. Kahn Jr, Andre Esteva, Alan Karthikesalingam, Bilal Mateen, Dale Webster, Dan Milea, Daniel Ting, Darren

For peer review only

Treanor, Dominic Cushnan, Dominic King, D AD. A quality assessment tool for artificial intelligence-centered diagnostic test accuracy studies: QUADAS-AI. *Nat Med* 2021;**27**:1663–5. doi:10.1038/s41591-021-01494-4

24 Campbell M, McKenzie JE, Sowden A, *et al.* Synthesis without meta-analysis (SWiM) in systematic reviews: Reporting guideline. *BMJ* 2020;**368**:1–6. doi:10.1136/bmj.l6890

25 Acar E, Leblebici A, Ellidokuz BE, *et al.* Full paper: Machine learning for differentiating metastatic and completely responded sclerotic bone lesion in prostate cancer: A retrospective radiomics study. *Br J Radiol* 2019;**92**. doi:http://dx.doi.org/10.1259/bjr.20190286

26 Coy H, Hsieh K, Wu W, *et al.* Deep learning and radiomics: the utility of Google TensorFlow™ Inception in classifying clear cell renal cell carcinoma and oncocytoma on multiphasic CT. *Abdom Radiol* 2019;**44**:2009–20. doi:10.1007/s00261-019-01929-0

27 Saiprasad G, Chang CI, Safdar N, *et al.* Adrenal gland abnormality detection using random forest classification. *J Digit Imaging* 2013;**26**:891–7. doi:http://dx.doi.org/10.1007/s10278-012-9554-7

28 Sethi G, Saini BS. Computer aided diagnosis system for abdomen diseases in computed tomography images. *Biocybern Biomed Eng* 2016;**36**:42–55. doi:10.1016/j.bbe.2015.10.008

29 Yasaka K, Akai H, Abe O, *et al.* Deep learning with CNN showed high diagnostic performance in differentiation of liver masses at dynamic CT. *Radiology* 2018;**286**:887–96.

30 Yuan Z, Xu T, Cai J, *et al.* Development and Validation of an Image-based Deep Learning Algorithm for Detection of Synchronous Peritoneal Carcinomatosis in

For peer review only

Colorectal Cancer. *Ann surgery* 2020;**16**. doi:http://dx.doi.org/10.1097/SLA.0000000000004229

31 Zhao X, Xie P, Wang M, *et al.* Deep learning-based fully automated detection and segmentation of lymph nodes on multiparametric-mri for rectal cancer: A multicentre study. *EBioMedicine* 2020;**56**:102780. doi:https://dx.doi.org/10.1016/j.ebiom.2020.102780

32 Han S, Hwang S Il, Lee HJ. The Classification of Renal Cancer in 3-Phase CT Images Using a Deep Learning Method. *J Digit Imaging* 2019;**32**:638–43. doi:10.1007/s10278-019-00230-2

33 Kawauchi K, Furuya S, Hirata K, *et al.* A convolutional neural network-based system to classify patients using FDG PET/CT examinations. *BMC Cancer* 2020;**20**:227. doi:https://dx.doi.org/10.1186/s12885-020-6694-x

34 Koizumi M, Motegi K, Koyama M, *et al.* Diagnostic performance of a computer-assisted diagnosis system for bone scintigraphy of newly developed skeletal metastasis in prostate cancer patients: search for low-sensitivity subgroups. *Ann Nucl Med* 2017;**31**:521–8. doi:http://dx.doi.org/10.1007/s12149-017-1175-2

35 Lee JJ, Yang H, Franc BL, *et al.* Deep learning detection of prostate cancer recurrence with 18F-FACBC (fluciclovine, Axumin®) positron emission tomography. *Eur J Nucl Med Mol Imaging* 2020;**47**:2992–7. doi:10.1007/s00259-020-04912-w

36 Lu Y, Yu Q, Gao Y, *et al.* Identification of metastatic lymph nodes in MR imaging with faster region-based convolutional neural networks. *Cancer Res* 2018;**78**:5135–43. doi:10.1158/0008-5472.CAN-18-0494

37 Nakagawa M, Nakaura T, Namimoto T, *et al.* A multiparametric MRI-based machine learning to distinguish between uterine sarcoma and benign

For peer review only

leiomyoma: comparison with 18F-FDG PET/CT. *Clin Radiol* 2019;**74**:167.e1-167.e7. doi:10.1016/j.crad.2018.10.010

38 Nayak A, Baidya Kayal E, Arya M, *et al.* Computer-aided diagnosis of cirrhosis and hepatocellular carcinoma using multi-phase abdomen CT. *Int J Comput Assist Radiol Surg* 2019;**14**:1341–52. doi:http://dx.doi.org/10.1007/s11548-019-01991-5

39 Oberai A, Varghese B, Cen S, *et al.* Deep learning based classification of solid lipid-poor contrast enhancing renal masses using contrast enhanced CT. *Br J Radiol* 2020;**93**:20200002. doi:http://dx.doi.org/10.1259/bjr.20200002

40 Garrett RW, Nepute JC, El Hayek M, *et al.* Adrenal Incidentalomas: Clinical controversies and modified recommendations. *Am J Roentgenol* 2016;**206**:1170–8. doi:10.2214/AJR.15.15475

41 Sounderajah V, Ashrafian H, Karthikesalingham A, Marker SR, Normanhani P, Collins GS, Bossuyt PM DD. Developing Specific Reporting Standards in Artificial Intelligence Centred Research Viknesh. *Ann Surg* 2021;:6. doi:10.1097/SLA.0000000000005294

42 Sounderajah V, Ashrafian H, Aggarwal R, *et al.* Developing specific reporting guidelines for diagnostic accuracy studies assessing AI interventions: The STARD-AI Steering Group. *Nat Med* 2020;**26**:807–8. doi:10.1038/s41591-020-0941-1

43 Cruz Rivera S, Liu X, Chan AW, *et al.* Guidelines for clinical trial protocols for interventions involving artificial intelligence: the SPIRIT-AI extension. *Nat Med* 2020;**26**:1351–63. doi:10.1038/s41591-020-1037-7

44 Liu X, Cruz Rivera S, Moher D, *et al.* Reporting guidelines for clinical trial reports for interventions involving artificial intelligence: the CONSORT-AI extension. *Nat*

For peer review only

Med 2020;**26**:1364–74. doi:10.1038/s41591-020-1034-x

45 Hernandez-Boussard T, Bozkurt S, Ioannidis JPA, *et al.* MINIMAR (MINimum Information for Medical AI Reporting): Developing reporting standards for artificial intelligence in health care. *J Am Med Informatics Assoc* 2020;**00**:1–5. doi:10.1093/jamia/ocaa088

46 Yin J, Ngiam KY TH. Role of Artificial Intelligence Applications in Real-Life Clinical Practice: Systematic Review. *J Med Internet Res* 2021;**23**:e25759. doi:10.2196/25759

For peer review only

Figure 1 PRISMA flow diagram

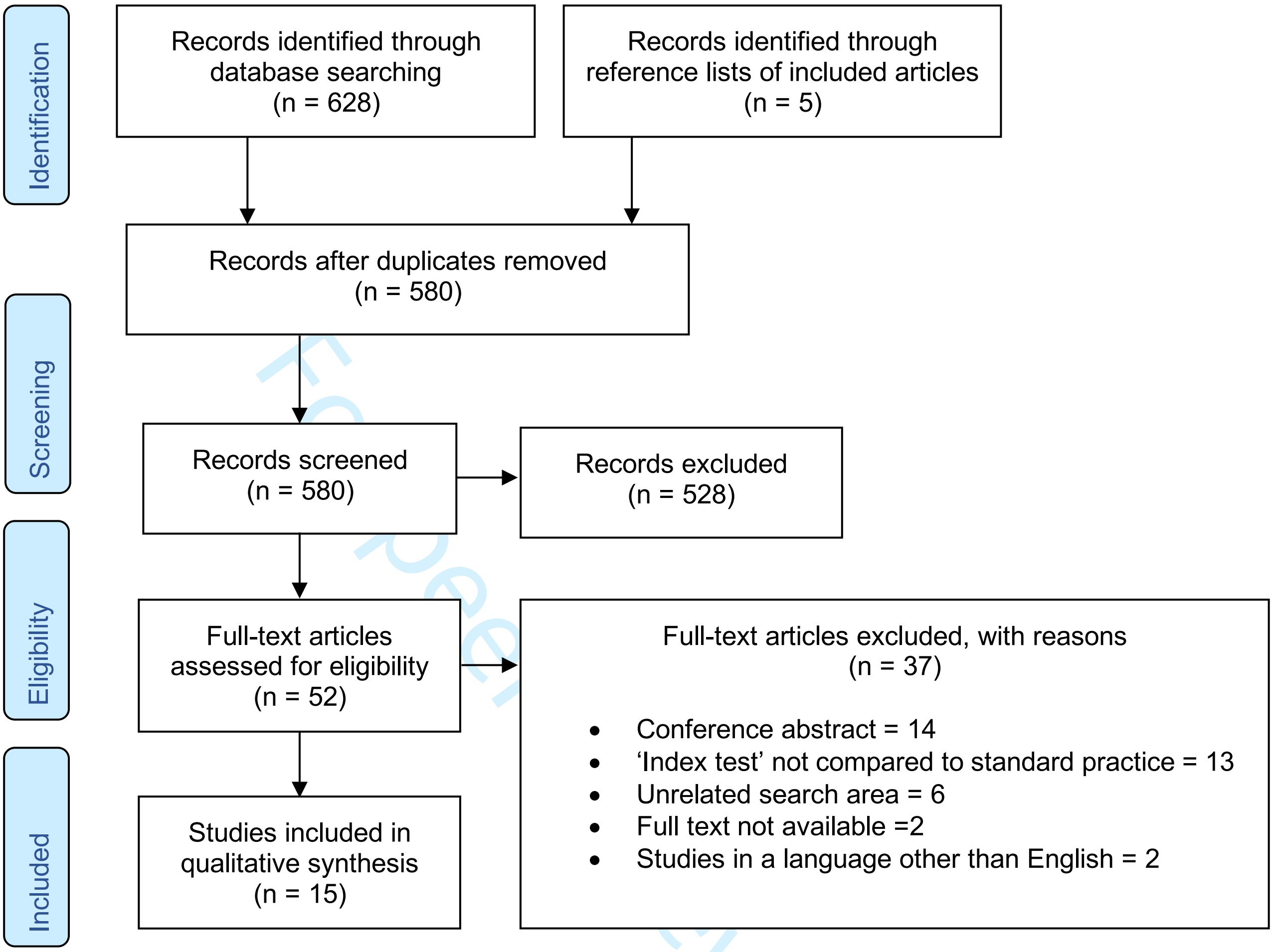

Online supplementary Table 1 Performance of AI models

First author (Reference)	Study aim	Best AI model	Output measures	AI performance	Human comparator	Human performance	External validation
**Acar [25]**	Distinguish the metastatic and completely responded areas regarding sclerotic bone lesions imaged via 68Ga-PSMA PET/ CT in patients with known bone metastasis.	KNN	Sensitivity<br>Specificity<br>Predictive values (PPV, NPV)<br>AUC<br>Overall accuracy	73.5%<br>73.7%<br>86.9%, 53.8%<br>76.0%<br>73.5%	NA	NA	No
**Coy [26]**	Investigate the diagnostic value and feasibility of a deep learning-based renal lesion classifier in differentiating ccRCC from ONC on routine four-phase MDCT in patients with pathologically confirmed renal masses.	CNN	Sensitivity<br>Specificity<br>Predictive values (PPV, NPV)<br>Overall accuracy	88.3%<br>52.9%<br>79.6%, 59.5%<br>75.4%	NA	NA	No
**Han [32]**	Develop reproducible and generalizable models for discriminating three major subtypes of RCCs using CT image analysis along with a ML algorithm.	CNN	Sensitivity<br>Specificity<br>AUC<br>Overall accuracy	0.98<br>0.93<br>0.9<br>0.85	NA	NA	No
**Koizumi [34]**	Investigate the diagnostic performance of computer-assisted diagnostic system for bone scintigraphy in prostate cancer patients with and without skeletal metastasis.	ANN	Sensitivity<br>Specificity<br>AUC	82.0%<br>83.0%<br>0.89	NA	NA	No
**Lee [35]**	Evaluate the performance of deep learning classifiers in discriminating normal and abnormal 18F-FACBC PET scans based on the presence of tumour recurrence and/or metastases in patients with	CNN	Sensitivity<br>Specificity<br>AUC	90.7%<br>95.1%<br>0.97	NA	NA	No

For peer review only

	prostate cancer and biochemical recurrence.						
**Oberai [39]**	Classify lipid poor enhancing renal masses into 'benign' and 'malignant' categories.	CNN	Sensitivity<br>Specificity<br>AUC<br>Overall accuracy	70.0%<br>81.0%<br>0.82<br>78.0%	NA	NA	No
**Lu [36]**	To develop an assistive tool to aid radiologists to more effectively and accurately determine lymph node metastases.	CNN	Sensitivity<br>Specificity<br>AUC<br>Overall accuracy	Not reported<br>Not reported<br>0.91<br>99.9%	Radiologists (number not specified)	Values not explicitly reported.	Yes
**Nayak [38]**	Study proposes a computer-aided diagnosis system for detecting cirrhosis and HCC.	SVM	Prediction accuracy	80.8 - 86.9%	NA	NA	No
**Sethi [28]**	Paper proposed automated CAD system for classification of abdomen diseases like tumour, cyst, stone and normal tissues from CT images of abdomen.	ANN	Sensitivity<br>Specificity<br>Predictive values (PPV, NPV)<br>AUC<br>Overall accuracy	95.0%<br>98.0%<br>No raw data<br>No raw data<br>95.1%	NA	NA	No
**Yasaka [29]**	Investigate diagnostic performance by using a deep learning method with a CNN for the differentiation of liver masses at dynamic contrast agent-enhanced CT.	CNN	Sensitivity<br>Specificity<br>AUC<br>Overall accuracy	0.33 - 1.00<br>Not reported<br>0.92<br>0.84	NA	NA	No
**Yuan [30]**	Develop a CNN-based ResNet3d algorithm and SVM classified to detect synchronous peritoneal carcinomatosis in colorectal cancer.	CNN + SVM combination	Sensitivity<br>Specificity<br>Predictive values (PPV, NPV)<br>AUC<br>Overall accuracy	93.8%<br>94.4%<br>93.8%, 94.4%<br>0.92<br>94.1%	2 Radiologists (senior and junior)	57.4%, 58.1%<br>100%, 98%<br>No raw data<br>0.79, 0.78<br>No raw data	Yes
**Zhao [31]**	Develop and validate a deep-learning-based, fully automated lymph node detection and segmentation model based on mpMRI.	CNN	Sensitivity<br><br>Predictive values (PPV, NPV)	80.0%<br><br>73.5%, NA	4 Radiologists	38.2% (Int), 62.6% (Ext)<br>43.8% (Int), 35.9% (Ext)	Yes
**Saiprasad [27]**	Identify adrenal glands from a volumetric CT acquisition and	RFC	Sensitivity<br>Specificity	80.0%<br>90.0%	NA	NA	No

	determine whether they are normal or abnormal.						
**Nakagawa [37]**	Compare the performance of ML using mp-MRI and PET to distinguish between uterine sarcoma and leiomyoma.	Logistic regression (multivariate)	AUC	0.92	2 Radiologists (11 and 20 years of experience)	0.97 and 0.89	Yes
**Kawauchi [33]**	Develop a CNN-based system that can classify whole-body FDG PET as 1) benign, 2) malignant or 3) equivocal.	CNN	Prediction	91.0% - 94.9% (abdomen) 95.3% - 99.7% (pelvic region)	NA	NA	No

Abbreviations: Area under the curve (AUC), benign oncocytoma (ONC), clear cell renal cell carcinoma (ccRCC), CNN (convolutional neural network), Computer tomography (CT), Ext (external data set), hepatocellular carcinoma (HCC), Int (internal data set), machine learning (ML), k-nearest neighbours algorithm (KNN), multiparametric magnetic resonance imaging (mpMRI), negative predictive value (NPV), positive predictive value (PPV), positron emission tomography (PET), random forest classification (RFC) and support vector machine (SVM).

.

For peer review only

APPENDIX S1

Artificial intelligence as a diagnostic aid in cross-sectional radiological imaging of surgical pathology in the abdominopelvic cavity: A systematic review

Medline Search

1. artificial intelligence/ or machine learning/ or deep learning/ or supervised machine learning/ or support vector machine/ or unsupervised machine learning/
2. computer heuristics/ or expert systems/ or fuzzy logic/ or knowledge bases/ or biological ontologies/ or gene ontology/ or natural language processing/ or neural networks, computer/ or robotics/
3. Machine learning.ti,ab,kf.
4. Artificial Intelligence.ti,ab,kf.
5. Naive Bayes.ti,ab,kf.
6. bayesian learning.ti,ab,kf.
7. Neural network*.ti,ab,kf.
8. Natural language processing.ti,ab,kf.
9. support vector*.ti,ab,kf.
10. random forest*.ti,ab,kf.
11. boosting.ti,ab,kf.
12. deep learning.ti,ab,kf.
13. machine intelligence.ti,ab,kf.
14. computational intelligence.ti,ab,kf.
15. computer reasoning.ti,ab,kf.
16. 1 or 2 or 3 or 4 or 5 or 6 or 7 or 8 or 9 or 10 or 11 or 12 or 13 or 14 or 15

For peer review only

17. tomography/ or colonography, computed tomographic/ or computed tomography angiography/ or positron emission tomography computed tomography/

18. magnetic resonance imaging/ or cholangiopancreatography, magnetic resonance/ or diffusion magnetic resonance imaging/ or diffusion tensor imaging/ or echo-planar imaging/ or fluorine-19 magnetic resonance imaging/ or magnetic resonance angiography/ or magnetic resonance imaging, cine/ or multiparametric magnetic resonance imaging/

19. (MRI or Magnetic Resonance Imaging).ti,ab,kf.

20. 17 or 18 or 19

21. pelvis/ or lesser pelvis/ or pelvic floor/

22. (radiography adj3 abdominal).ti,ab,kf.

23. (Abdomen or abdominal cavity or peritoneum or douglas' pouch or mesentery or mesocolon or omentum or peritoneal cavity or peritoneal stomata or retroperitoneal space).ti,ab,kf.

24. (Pelvis or pelvic floor).ti,ab,kf.

25. 21 or 22 or 23 or 24

26. exp Animals/ not Humans/

27. (animal model* or rat or rats or mouse or mice or rodent* or sheep or lambs or murine or pigs or piglets or swine or porcine or rabbit or rabbits or cat or cats or feline or dog or dogs or canine or cattle or bovine or marmoset* or monkey or monkeys or trout or zebra fish*).ti.

28. (Comment or editorial or letter or case reports).pt.

29. (endoscope or gastroscope or colonoscope or capsule endoscopy or endoscopy).ti,ab,kf.

30. 26 or 27 or 28 or 29

For peer review only

31. 16 and 20 and 25

32. 31 not 30

33. (2012* or 2013* or 2014* or 2015* or 2016* or 2017* or 2018* or 2019* or 2020* or 202101*).yr,ed,dc,ep.

34. 32 and 33

For peer review only

Embase via Ovid

1. artificial intelligence/ or ambient intelligence/ or automated reasoning/ or computer heuristics/ or multicriteria decision analysis/
2. exp machine learning/
3. deep learning/
4. supervised machine learning/
5. exp support vector machine/
6. unsupervised machine learning/
7. hyperheuristics/ or metaheuristics/
8. expert systems/ or fuzzy logic/ or knowledge bases/ or natural language processing/ or neural networks, computer/ or robotics/
9. biological ontologies/ or gene ontology/
10. ((machine or bayesian or deep) adj1 learning).ti,ab,kw.
11. ((artificial or machine or computational) adj1 intelligence).ti,ab,kw.
12. Naive Bayes.ti,ab,kw.
13. Neural network*.ti,ab,kw.
14. Natural language processing.ti,ab,kw.
15. support vector*.ti,ab,kw.
16. random forest*.ti,ab,kw.
17. boosting.ti,ab,kw.
18. computer reasoning.ti,ab,kw.
19. 1 or 2 or 3 or 4 or 5 or 6 or 7 or 8 or 9 or 10 or 11 or 12 or 13 or 14 or 15 or 16 or 17 or 18
20. exp Tomography/

For peer review only

21. colonography, computed tomographic/ or computed tomography angiography/ or four-dimensional computed tomography/ or positron emission tomography computed tomography/ or single photon emission computed tomography computed tomography/ or tomography, spiral computed/

22. magnetic resonance imaging/ or cholangiopancreatography, magnetic resonance/ or diffusion magnetic resonance imaging/ or diffusion tensor imaging/ or echo-planar imaging/ or fluorine-19 magnetic resonance imaging/ or magnetic resonance angiography/ or magnetic resonance imaging, cine/ or multiparametric magnetic resonance imaging/

23. (MRI or Magnetic Resonance Imaging).ti,ab,kw.

24. 20 or 21 or 22 or 23

25. pelvis/ or lesser pelvis/ or pelvic floor/

26. (radiography adj3 abdominal).ti,ab,kw.

27. (Abdomen or abdominal cavity or peritoneum or douglas' pouch or mesentery or mesocolon or omentum or peritoneal cavity or peritoneal stomata or retroperitoneal space).ti,ab,kw.

28. (Pelvis or pelvic floor).ti,ab,kw.

29. 25 or 26 or 27 or 28

30. (Animal/ or Nonhuman/) not Human/

31. Animal Experiment/ not (Human Experiment/ or Human/)

32. (animal model* or rat or rats or mouse or mice or rodent* or sheep or lambs or murine or pigs or piglets or swine or porcine or rabbit or rabbits or cat or cats or feline or dog or dogs or canine or cattle or bovine or marmoset* or monkey or monkeys or trout or zebra fish*).ti.

33. (Comment or editorial or letter or case reports).pt.

For peer review only

34. (endoscope or gastroscope or colonoscope or capsule endoscopy or endoscopy).ti,ab,kw.

35. 30 or 31 or 32 or 33 or 34

36. 19 and 24 and 29

37. 36 not 35

38. (2012* or 2013* or 2014* or 2015* or 2016* or 2017* or 2018* or 2019* or 2020* or 202101*).yr,em.

39. 37 and 38

For peer review only

Cochrane Central Register of Controlled Trials

#1 MeSH descriptor: [Artificial Intelligence] this term only MeSH

#2 MeSH descriptor: [Machine Learning] this term only MeSH

#3 MeSH descriptor: [Deep Learning] this term only MeSH

#4 MeSH descriptor: [Supervised Machine Learning] this term only MeSH

#5 MeSH descriptor: [Unsupervised Machine Learning] this term only MeSH

#6 MeSH descriptor: [Support Vector Machine] this term only MeSH

#7 MeSH descriptor: [Computer Heuristics] this term only MeSH

#8 MeSH descriptor: [Expert Systems] this term only MeSH

#9 MeSH descriptor: [Fuzzy Logic] this term only MeSH

#10 MeSH descriptor: [Knowledge Bases] this term only MeSH

#11 MeSH descriptor: [Biological Ontologies] this term only MeSH

#12 MeSH descriptor: [Gene Ontology] this term only MeSH

#13 MeSH descriptor: [Natural Language Processing] this term only MeSH

#14 MeSH descriptor: [Neural Networks, Computer] this term only MeSH

#15 MeSH descriptor: [Robotics] this term only MeSH

#16 (machine learning): ti,ab,kw

#17 (artificial intelligence):ti,ab,kw

#18 (naive bayes):ti,ab,kw

#19 (bayesian learning):ti,ab,kw

#20 (neural network*):ti,ab,kw

#21 (natural language processing):ti,ab,kw

#22 (support vector*):ti,ab,kw

#23 (random forest*):ti,ab,kw

#24 (boosting):ti,ab,kw

For peer review only

#25 (deep learning):ti,ab,kw

#26 (machine intelligence):ti,ab,kw

#27 (computational intelligence):ti,ab,kw

#28 (computer reasoning):ti,ab,kw

#29 #1 or #2 or #3 or #4 or #5 or #6 or #7 or #8 or #9 or #10 or #11 or #12 or #13 or #14 or #15 or #16 or #17 or #18 or #19 or #20 or #21 or #22 or #23 or #24 or #25 or #26 or #27 or #28

#30 MeSH descriptor: [Tomography] this term only MeSH

#31 MeSH descriptor: [Colonography, Computed Tomographic] this term only MeSH

#32 MeSH descriptor: [Computed Tomography Angiography] this term only MeSH

#33 MeSH descriptor: [Positron-Emission Tomography] this term only MeSH

#34 MeSH descriptor: [Positron Emission Tomography Computed Tomography] this term only MeSH

#35 MeSH descriptor: [Magnetic Resonance Imaging] this term only MeSH

#36 MeSH descriptor: [Cholangiopancreatography, Magnetic Resonance] this term only MeSH

#37 MeSH descriptor: [Diffusion Magnetic Resonance Imaging] this term only MeSH

#38 MeSH descriptor: [Diffusion Tensor Imaging] this term only MeSH

#39 MeSH descriptor: [Echo-Planar Imaging] this term only MeSH

#40 MeSH descriptor: [Fluorine-19 Magnetic Resonance Imaging] this term only MeSH

#41 MeSH descriptor: [Magnetic Resonance Angiography] this term only MeSH

#42 MeSH descriptor: [Magnetic Resonance Imaging, Cine] this term only MeSH

#43 MeSH descriptor: [Multiparametric Magnetic Resonance Imaging] this term only MeSH

For peer review only

#44 (MRI):ti,ab,kw

#45 (magnetic resonance imaging):ti,ab,kw

#46 #30 or #31 or #32 or #33 or #34 or #35 or #36 or #37 or #38 or #39 or #40 or #41 or #42 or #43 or #44 or #45

#47 MeSH descriptor: [Pelvis] this term only MeSH

#48 MeSH descriptor: [Lesser Pelvis] this term only MeSH

#49 MeSH descriptor: [Pelvic Floor] this term only MeSH

#50 ((radiography NEAR/3 abdominal)):ti,ab,kw

#51 ((radiography NEAR/3 abdomen)):ti,ab,kw

#52 ((Abdomen or abdominal cavity or peritoneum or douglas' pouch or mesentery or mesocolon or omentum or peritoneal cavity or peritoneal stomata or retroperitoneal space)):ti,ab,kw

#53 (pelvis or pelvic floor):ti,ab,kw

#54 #47 or #48 or #49 or #50 or #51 or #52 or #53

#55 #29 and #46 and #54

For peer review only

PRISMA-DTA for Abstracts Checklist

Section/topic	#	PRISMA-DTA for Abstracts Checklist item	Reported on page #
**TITLE and PURPOSE**			
Title	1	Identify the report as a systematic review (+/- meta-analysis) of diagnostic test accuracy (DTA) studies.	1
Objectives	2	Indicate the research question, including components such as participants, index test, and target conditions.	4
**METHODS**			
Eligibility criteria	3	Include study characteristics used as criteria for eligibility.	4
Information sources	4	List the key databases searched and the search dates.	4
Risk of bias & applicability	5	Indicate the methods of assessing risk of bias and applicability.	4
Synthesis of results	A1	Indicate the methods for the data synthesis.	4
**RESULTS**			
Included studies	6	Indicate the number and type of included studies and the participants and relevant characteristics of the studies (including the reference standard).	4
Synthesis of results	7	Include the results for the analysis of diagnostic accuracy, preferably indicating the number of studies and participants. Describe test accuracy including variability; if meta-analysis was done, include summary results and confidence intervals.	4
**DISCUSSION**			
Strengths and limitations	9	Provide a brief summary of the strengths and limitations of the evidence	-
Interpretation	10	Provide a general interpretation of the results and the important implications.	4-5
**OTHER**			
Funding	11	Indicate the primary source of funding for the review.	2
Registration	12	Provide the registration number and the registry name	8

Adapted From: McInnes MDF, Moher D, Thombs BD, McGrath TA, Bossuyt PM, The PRISMA-DTA Group (2018). Preferred Reporting Items for a Systematic Review and Meta-analysis of Diagnostic Test Accuracy Studies: The PRISMA-DTA Statement. JAMA. 2018 Jan 23;319(4):388-396. doi: 10.1001/jama.2017.19163.

For more information, visit: www.prisma-statement.org.

Page 1 of 1

For peer review only

PRISMA-DTA Checklist

Section/topic	#	PRISMA-DTA Checklist Item	Reported on page #
**TITLE / ABSTRACT**			
Title	1	Identify the report as a systematic review (+/- meta-analysis) of diagnostic test accuracy (DTA) studies.	1
Abstract	2	Abstract: See PRISMA-DTA for abstracts.	4-5
**INTRODUCTION**			
Rationale	3	Describe the rationale for the review in the context of what is already known.	6-7
Clinical role of index test	D1	State the scientific and clinical background, including the intended use and clinical role of the index test, and if applicable, the rationale for minimally acceptable test accuracy (or minimum difference in accuracy for comparative design).	6-7
Objectives	4	Provide an explicit statement of question(s) being addressed in terms of participants, index test(s), and target condition(s).	9
**METHODS**			
Protocol and registration	5	Indicate if a review protocol exists, if and where it can be accessed (e.g., Web address), and, if available, provide registration information including registration number.	7
Eligibility criteria	6	Specify study characteristics (participants, setting, index test(s), reference standard(s), target condition(s), and study design) and report characteristics (e.g., years considered, language, publication status) used as criteria for eligibility, giving rationale.	9
Information sources	7	Describe all information sources (e.g., databases with dates of coverage, contact with study authors to identify additional studies) in the search and date last searched.	8
Search	8	Present full search strategies for all electronic databases and other sources searched, including any limits used, such that they could be repeated.	8-9
Study selection	9	State the process for selecting studies (i.e., screening, eligibility, included in systematic review, and, if applicable, included in the meta-analysis).	8-9
Data collection process	10	Describe method of data extraction from reports (e.g., piloted forms, independently, in duplicate) and any processes for obtaining and confirming data from investigators.	9-11
Definitions for data extraction	11	Provide definitions used in data extraction and classifications of target condition(s), index test(s), reference standard(s) and other characteristics (e.g. study design, clinical setting).	9-11
Risk of bias and applicability	12	Describe methods used for assessing risk of bias in individual studies and concerns regarding the applicability to the review question.	11
Diagnostic accuracy measures	13	State the principal diagnostic accuracy measure(s) reported (e.g. sensitivity, specificity) and state the unit of assessment (e.g. per-patient, per-lesion).	10-11
Synthesis of results	14	Describe methods of handling data, combining results of studies and describing variability between studies. This could include, but is not limited to: a) handling of multiple definitions of target condition. b) handling of multiple thresholds of test positivity, c) handling multiple index test readers, d) handling of indeterminate test results, e) grouping and comparing tests, f) handling of different reference standards	11

For peer review only - http://bmjopen.bmj.com/site/about/guidelines.xhtml

PRISMA-DTA Checklist

Section/topic	#	PRISMA-DTA Checklist Item	Reported on page #
Meta-analysis	D2	Report the statistical methods used for meta-analyses, if performed.	NA
Additional analyses	16	Describe methods of additional analyses (e.g., sensitivity or subgroup analyses, meta-regression), if done, indicating which were pre-specified.	11
**RESULTS**			
Study selection	17	Provide numbers of studies screened, assessed for eligibility, included in the review (and included in meta-analysis, if applicable) with reasons for exclusions at each stage, ideally with a flow diagram.	12
Study characteristics	18	For each included study provide citations and present key characteristics including: a) participant characteristics (presentation, prior testing), b) clinical setting, c) study design, d) target condition definition, e) index test, f) reference standard, g) sample size, h) funding sources	12-13
Risk of bias and applicability	19	Present evaluation of risk of bias and concerns regarding applicability for each study.	14
Results of individual studies	20	For each analysis in each study (e.g. unique combination of index test, reference standard, and positivity threshold) report 2x2 data (TP, FP, FN, TN) with estimates of diagnostic accuracy and confidence intervals, ideally with a forest or receiver operator characteristic (ROC) plot.	NA
Synthesis of results	21	Describe test accuracy, including variability; if meta-analysis was done, include results and confidence intervals.	12-14
Additional analysis	23	Give results of additional analyses, if done (e.g., sensitivity or subgroup analyses, meta-regression; analysis of index test: failure rates, proportion of inconclusive results, adverse events).	NA
**DISCUSSION**			
Summary of evidence	24	Summarize the main findings including the strength of evidence.	15-16
Limitations	25	Discuss limitations from included studies (e.g. risk of bias and concerns regarding applicability) and from the review process (e.g. incomplete retrieval of identified research).	16
Conclusions	26	Provide a general interpretation of the results in the context of other evidence. Discuss implications for future research and clinical practice (e.g. the intended use and clinical role of the index test).	17
**FUNDING**			
Funding	27	For the systematic review, describe the sources of funding and other support and the role of the funders.	2

Adapted From: McInnes MDF, Moher D, Thombs BD, McGrath TA, Bossuyt PM, The PRISMA-DTA Group (2018). Preferred Reporting Items for a Systematic Review and Meta-analysis of Diagnostic Test Accuracy Studies: The PRISMA-DTA Statement. JAMA. 2018 Jan 23;319(4):388-396. doi: 10.1001/jama.2017.19163.

For more information, visit: **www.prisma-statement.org**.

For peer review only - http://bmjopen.bmj.com/site/about/guidelines.xhtml

BMJ Open

Artificial intelligence as a diagnostic aid in cross-sectional radiological imaging of surgical pathology in the abdominopelvic cavity: A systematic review

Journal:	*BMJ Open*
Manuscript ID	bmjopen-2022-064739.R1
Article Type:	Original research
Date Submitted by the Author:	16-Sep-2022
Complete List of Authors:	Fowler, George; NIHR Bristol Biomedical Research Centre, Population Health Sciences, Bristol Medical School. University of Bristol, UK<br>Blencowe, Natalie; NIHR Bristol Biomedical Research Centre, Population Health Sciences, Bristol Medical School. University of Bristol, UK<br>Hardacre, Conor; Bristol Medical School<br>Callaway, Mark; Bristol Royal Infirmary, Department of Clinical Radiology<br>Smart , Neil ; Royal Devon and Exeter NHS Foundation Trust, Exeter Surgical Health Services Research Unit (HeSRU)<br>Macefield, Rhiannon; NIHR Bristol Biomedical Research Centre, Population Health Sciences, Bristol Medical School. University of Bristol, UK
**Primary Subject Heading**:	Surgery
Secondary Subject Heading:	Diagnostics
Keywords:	SURGERY, Diagnostic radiology < RADIOLOGY & IMAGING, Adult surgery < SURGERY

SCHOLARONE™
Manuscripts

I, the Submitting Author has the right to grant and does grant on behalf of all authors of the Work (as defined in the below author licence), an exclusive licence and/or a non-exclusive licence for contributions from authors who are: i) UK Crown employees; ii) where BMJ has agreed a CC-BY licence shall apply, and/or iii) in accordance with the terms applicable for US Federal Government officers or employees acting as part of their official duties; on a worldwide, perpetual, irrevocable, royalty-free basis to BMJ Publishing Group Ltd ("BMJ") its licensees and where the relevant Journal is co-owned by BMJ to the co-owners of the Journal, to publish the Work in this journal and any other BMJ products and to exploit all rights, as set out in our licence.

The Submitting Author accepts and understands that any supply made under these terms is made by BMJ to the Submitting Author unless you are acting as an employee on behalf of your employer or a postgraduate student of an affiliated institution which is paying any applicable article publishing charge ("APC") for Open Access articles. Where the Submitting Author wishes to make the Work available on an Open Access basis (and intends to pay the relevant APC), the terms of reuse of such Open Access shall be governed by a Creative Commons licence – details of these licences and which Creative Commons licence will apply to this Work are set out in our licence referred to above.

Other than as permitted in any relevant BMJ Author's Self Archiving Policies, I confirm this Work has not been accepted for publication elsewhere, is not being considered for publication elsewhere and does not duplicate material already published. I confirm all authors consent to publication of this Work and authorise the granting of this licence.

For peer review only

Artificial intelligence as a diagnostic aid in cross-sectional radiological imaging of surgical pathology in the abdominopelvic cavity: A systematic review

For peer review only

Authors: George E Fowler[1]
george.fowler@bristol.ac.uk

Natalie S Blencowe[1]
natalie.blencowe@bristol.ac.uk

Conor Hardacre[2]
ch17008@bristol.ac.uk

Mark P Callaway[3]
mark.callaway@uhbw.nhs.uk

Neil J Smart[4]
drneilsmart@hotmail.com

Rhiannon C Macefield[1]
r.macefield@bristol.ac.uk

Corresponding author: George E Fowler
NIHR Bristol Biomedical Research Centre, Population Health Sciences, Bristol Medical School. University of Bristol, UK.

Institutions: [1]NIHR Bristol Biomedical Research Centre, Population Health Sciences, Bristol Medical School. University of Bristol, UK.
[2]Bristol Medical School, University of Bristol, UK.
[3]Department of Clinical Radiology, University Hospital Bristol and Weston NHS Foundation Trust, Bristol, UK.

[4]Exeter Surgical Health Services Research Unit (HeSRU), Royal Devon and Exeter Hospital, Exeter, UK.

ORCID ID(s):
GEF (0000-0002-4133-802X)
RCM (0000-0002-6606-5427)
CH (0000-0001-9800-0479)
MPC
NJS (0000-0002-3043-8324)
NSB (0000-0002-6111-2175)

Acknowledgements: The authors would like to thank Ms Catherine Borwick, Information Specialist, University of Bristol, for her input and expertise to develop the search strategy. The authors are grateful to Dr Christin Hoffmann (University of Bristol) for leading patient and public feedback sessions as part of the wider programme of this research and Dr Xiaoxuan Liu (University of Birmingham and University Hospitals Birmingham NHS Foundation Trust) for providing expert comments on the manuscript.

Category: Systematic review.

Key words: Artificial intelligence; deep learning; systematic review; surgery; diagnostic

Word count: 2,541 words

For peer review only

Abstract

Objectives

There is emerging use of artificial intelligence (AI) models to aid diagnostic imaging. This review examined and critically appraised the application of AI models to identify surgical pathology from radiological images of the abdominopelvic cavity, to identify current limitations and inform future research.

Methods

Systematic database searches (Medline, EMBASE, Cochrane Central Register of Controlled Trials) were performed. Date limitations (January 2012-July 2021) were applied. Study characteristics, descriptions of AI models and outcomes assessing diagnostic performance were extracted by independent reviewers. A narrative synthesis was performed in accordance with the Synthesis Without Meta-analysis guidelines. Risk of bias was assessed (QUADAS-2).

Results

Fifteen retrospective studies were included. Studies were diverse in surgical speciality, the intention of the AI applications and the models used. AI training and test sets comprised a median of 130 (range: 5–2440) and 37 (range: 10–1045) patients respectively. Diagnostic performance of models varied (range: 70-95% sensitivity, 53%–98% specificity). Only four studies compared the AI model with human performance. Reporting of studies was unstandardised and often lacking in detail. Most studies (n=14) were judged as having overall high risk of bias with concerns regarding applicability. Limitations included English language publications only.

For peer review only

Conclusion

AI application in this field is diverse. Adherence to reporting guidelines is warranted. With finite healthcare resources, future endeavours may benefit from targeting areas where radiological expertise is in high demand to provide greater efficiency in clinical care. Translation to clinical practice and adoption of a multidisciplinary approach should be of high priority.

PROSPERO registration number: CRD42021237249.

For peer review only

BOX: Strengths and limitations of this study

- This systematic review examined and critically appraised the application of artificial intelligence models to identify surgical pathology from cross-sectional radiological images, including CT, MR, CT-PET and bone scans, and identified current limitations and evidence gaps, and thereby focusing future research efforts.
- Robust methodology was undertaken including screening, data extraction and of risk of bias assessment by two independent reviewers.
- Findings may be limited by including English language publications only.

For peer review only

Introduction

The widespread adoption of digital healthcare provides vast data to enable the application of artificial intelligence (AI) in pattern recognition [1]. This can alleviate the burden of, or replace, tasks traditionally dependent on clinicians. Examples include the interpretation of medical images for diagnostic, prognostic, surveillance, and management decisions, which otherwise rely on a limited number of interpreters and human resources [2]. There has been a surge of research into the use of AI in diagnostic imaging, exploring how it can support clinicians and provide greater efficacy and efficiency in clinical care [3].

Systematic reviews on the diagnostic accuracy of AI in medical imaging (including respiratory medicine, ophthalmology and breast cancer) [4], gastroenterology [5], neurosurgery [6] and vascular surgery [7] have demonstrated the diverse application of AI models to detect many pathologies. A variety of imaging modalities have been explored (e.g., computer tomography (CT), magnetic resonance (MR) and positron emission tomography (PET)). AI models can demonstrate diagnostic performances equivalent to that of experts [8] and with greater efficiency, for example, in the time taken to diagnose childhood cataracts (5.7 minutes quicker than senior consultants) [9]. However, while AI technologies offer to markedly reduce the clinical workload, “black box” techniques (AI algorithms) can be difficult or impossible to interpret, which can be a barrier to adopting these techniques into clinical practice. Furthermore, many AI studies are proof-of-concept [10] and poorly reported [11,12], including limited details on participants, making it difficult to replicate or interpret the study findings [12].

For peer review only

A systematic review of the diagnostic accuracy of AI models in cross-sectional radiological imaging of the abdominopelvic cavity is lacking [13]. Synthesis of the current AI research in this area could benefit several different surgical specialities which image this region, such as endocrine surgery, gastrointestinal surgery, obstetrics and gynaecology, urology and vascular surgery to guide their clinical decision making. This study aimed to conduct a systematic review to examine and critically appraise the application of AI models to identify surgical pathology from cross-sectional radiological images, including CT, MR, CT-PET and bone scans, of the abdominopelvic cavity, to identify current limitations and inform future research efforts.

For peer review only

METHODS

Protocol and registration

This systematic review was registered with the International Prospective Register of Systematic Reviews (PROSPERO: CRD42021237249). A study protocol has previously been published [13]. It is reported in accordance with the Preferred Reporting Items for Systematic Reviews and Meta-analysis of Diagnostic Test Accuracy Studies (PRISMA-DTA) [14].

Information sources

Electronic searches of OVID SP versions of Medline, EMBASE and the Cochrane Central Register of Controlled Trials (CENTRAL) databases were conducted to identify all potentially relevant studies. Date limitations between January 1st 2012 and July 31st 2021 were applied, to account for advancements in machine learning performance and the development of deep learning approaches since 2012, in line with existing reviews [15]. Reference lists of included articles were screened to identify further relevant studies.

Search strategy and study selection

A comprehensive search syntax was developed with adaptation from three existing search strategies [6,7,16] and guidance from an information specialist using text words and Medical Subject Headings (MeSH) related to three domains: "artificial intelligence", "diagnostic imaging" and the "abdominopelvic cavity" (Appendix S1). Database search results were imported into reference management software (EndNote X9, Clarivate Analytics, USA) and duplicates removed.

For peer review only

Assessment of study eligibility was performed in two stages. First, titles and abstracts were screened for inclusion by two independent reviewers (GEF and CH). Any conflicts were resolved through discussion, referring to the wider study team if required. Final eligibility was assessed by a full-text review of potentially eligible studies by the same process. Management of the screening process was aided by Rayyan software (Rayyan Systems Inc., Cambridge, MA, USA) [17].

Eligibility criteria

Primary research studies were considered for eligibility using the PIRT (participants, index test(s), reference standard and target condition) framework [18]. Participants were adults with pathology within the abdominopelvic cavity diagnosed using the following radiological modalities: CT, MR, CT-PET or bone scans. Diagnostic endoscopy was excluded as existing reviews have explored the performance of AI models in this area [19,20]. The index test was studies considering AI models as an intervention with the aim to provide a diagnosis. The reference standard was 'standard practice' to allow for variation across the included studies. The target condition was abdominopelvic cavity pathology which has had, or may warrant, an invasive procedure [21] for therapeutic intent.

For peer review only

Excluded were secondary research studies (e.g., systematic reviews), case reports and case series, absence of full text (e.g., conference abstracts), animal studies and non-English articles.

Data extraction and management

Data extraction from the included articles was independently performed by two reviewers (GEF and CH). Data management software (REDCap version 9.5.23, Vanderbilt University, USA) [22] and a pre-designed standardized form were used. Data were extracted under the following three subheadings:

(1) Study characteristics

Extracted data included the name of the first author, their affiliated country, composition of the study team (e.g., software engineers, radiologists, and surgeons who routinely operate on surgical pathology within the abdominopelvic cavity (e.g., gastrointestinal surgeons, urologists and gynaecologists)), year of publication, study aim and design (i.e., 'prospective' or 'retrospective'), surgical subspeciality and pathology studied (i.e., benign, malignant tumours, multiple or other). Information on the reporting of ethics and/or regulatory approval (e.g., Medicines and Healthcare products Regulatory Agency), patient and publication involvement and authors' mention of using a reporting guideline (e.g., Standards for Reporting of Diagnostic Accuracy Studies) was recorded.

(2) Training data

Extracted data on the input features (data used to develop the AI model) included the modality of cross-sectional radiological imaging (CT, MR, CT-PET and bone scans), the AI model used, the reference standard and the reporting and size of the training and test sets. Information on whether the training data came from the studies collected data or readily available datasets was recorded.

(3) Outcomes

For peer review only

The performance of the AI models and human comparator (where applicable) were extracted. Diagnostic measures of accuracy included reported sensitivity, specificity, positive predictive values and the area under the receiver operating characteristic curve (AUC). The interpretation time (seconds) for studies comparing the performance of the AI model with a human comparator (where reported) were extracted.

Risk of bias and applicability

Risk of bias was assessed independently by two reviewers (GEF and RCM) using the QUADAS-2 tool [23]. A version of the QUADAS-2 tool for AI studies [24] was still in development and not available at the time of conducting the current review. The generic QUADAS-2 tool with the pre-existing modified signalling questions was used to assess four domains including patient selection, index test, reference standard and flow and timing [23]. An overall judgement of “at risk of bias” or “concerns regarding applicability” was assigned if more one or more domains were judged as “high” or “unclear” [23]. Judgements of applicability assessed whether the study matched the review question [23].

Data Synthesis

A narrative synthesis was conducted according to the Synthesis Without Meta-analysis guidelines [25]. The synthesis was planned to focus on the primary outcome with studies grouped by the modality of radiological imaging, surgical subspeciality and pathology studied, as outlined in the protocol [13]. A broader approach was, however, adopted due to the small number of included studies and their heterogeneity. A meta-analysis was not performed due to the broad nature of the included studies.

For peer review only

Patient and Public Involvement

As part of the wider programme of work (Bristol Biomedical Research Centre, National Institute for Health Research (NIHR) Bristol BRC), patients and the public were consulted on their views of AI being used to guide doctors to make decisions about treatment. Overall, it was perceived positively, and they were supportive of its adoption in healthcare.

For peer review only

RESULTS

Database searching identified 628 records, with a further five studies identified through reference lists of included articles. After the removal of duplicates, 580 were screened and, 52 full-text articles were assessed for eligibility. Fifteen studies were finally included (Figure 1) [26–40].

Study characteristics

Characteristics of the included studies and details of the AI models are summarised (Tables 1 and 2). All were retrospective studies conducted in six different countries: Japan (n=4), USA (n=4), China (n=3), India (n=2), Turkey (n=1) and South Korea (n=1). Studies were all proof-of-concept (i.e., not applied in a clinical setting;), from four surgical specialities: urology (n=6), gastrointestinal surgery (n=6), endocrine surgery (n=1) and gynaecology (n=1). One study was not specific to a single speciality and involved whole-body CT-PET across four anatomical regions (head-and-neck, chest, abdomen, and pelvis)[29]. Most studies focused on malignant tumours (n=11) using CT as the imaging modality (n=8). Most studies (n=14) included an ethical approval statement. No studies, however, mentioned patient and public involvement or the use of a reporting guideline.

For peer review only

Table 1 Characteristics of the included studies

Characteristics	Number of studies n=15
**Country of origin**	
Japan	4
USA	4
China	3
India	2
Turkey	1
South Korea	1
**Year of publication**	
2020	5
2019	5
2018	2
2017	1
2016	1
2013	1
**Surgical subspecialty**	
Urology	6
Gastrointestinal	6
Endocrine	1
Gynaecology	1
Not reported	1
**Pathology studied**	
Malignant tumours	11
Multiple pathologies	2
Other	2
**Modality of radiological imaging**	
CT	8
CT-PET	2
MR	2
PET	1
MR and CT-PET	1
Bone scans	1

Abbreviations: CT (computer tomography), PET (positron emission tomography) and MR (magnetic resonance).

For peer review only

Table 2 Study details and AI models

First author (Reference)	Country of origin	Surgical speciality	Pathology studied	Modality of radiological imaging	AI models	Reference standard	Size of training set (patients)	Size of test set (patients)
**Acar [25]**	Turkey	Urology	Malignancy	CT-PET	KNN	2 Nuclear Medicine Physicians	"90% [total 75 patients]"	"10% [total 75 patients]"
**Coy [26]**	USA	Urology	Malignancy	CT	CNN	Pathologist (number not specified)	90% [total 179 patients]	10% [total 179 patients]
**Han [32]**	South Korea	Urology	Malignancy	CT	CNN	Pathologist (number not specified)	135	34
**Koizumi [34]**	Japan	Urology	Malignancy	Bone scans	ANN	2 Radiology Nuclear Physicians	Not reported	226
**Lee [35]**	USA	Urology	Malignancy	PET	CNN	1 Imaging Physicians	Not reported	Not reported
**Oberai [39]**	USA	Urology	Malignancy	CT	CNN	1 Genitourinary Pathologists	120	23
**Lu [36]**	China	Gastrointestinal	Malignancy	MR	CNN	Radiologists (number not specified)	Not reported	Not reported (+ 414 validation test set)
**Nayak [38]**	India	Gastrointestinal	Multiple (cirrhosis, hepatocellular carcinoma)	CT	SVM	1 Radiologist	40	Not reported
**Sethi [28]**	India	Gastrointestinal	Multiple (normal, cystic, calculus or tumour tissues)	CT	ANN, SVM	Unclear	Not reported	Not reported
**Yasaka [29]**	Japan	Gastrointestinal	Malignancy	CT	CNN	Radiologists (number not specified)	460	100

**Yuan [30]**	China	Gastrointestinal	Malignancy	CT	SVM, CNN	1 Pathologist	130	40
**Zhao [31]**	China	Gastrointestinal	Malignancy	MR	CNN	3 Radiologists	293	81
**Saiprasad [27]**	USA	Endocrine Surgery	Other (normal vs abnormal)	CT	RFC	2 Radiologists	5	10
**Nakagawa [37]**	Japan	Gynaecology	Malignancy	MR and CT-PET	Logistic regression (univariate and multivariate)	2 Radiologists and pathologists (number not specified)	66	Unclear
**Kawauchi, [33]**	Japan	Uncharacterized	Other (benign/malignant /equivocal)	CT-PET	CNN	1 Nuclear Medicine Physician	2440	1045

Abbreviations: Artificial neural network (ANN), Computerized tomography (CT), convolutional neural network (CNN), k-nearest neighbours algorithm (KNN), magnetic resonance (MR), positron emission tomography (PET), random forest classification (RFC) and support vector machine (SVM).

For peer review only

The composition and expertise within each study team varied. Three studies (n=3) comprised teams including software engineers, radiologists and surgeons. A small number of studies were comprised of only radiologists (n=2) or software engineers alone (n=1). Four study teams were comprised software engineers and radiologists together. Of the remaining five studies, three had no apparent radiological team and two were comprised of radiologists with either a team of software engineers, surgeons or physicians.

Training data

AI training and test sets within the included studies comprised a median of 130 (range: 5 – 2440) and 37 (range: 10 – 1045) patients, respectively (Table 2). This information, however, was not always available (n=6 studies). All training data came from the studies collected data and not from pre-existing readily available datasets. There was variability in the reference standard, including the number of clinicians (range: 1–3) for either a radiological (n=9) or histological (n=4) diagnosis or both (n=1). Only one study had an unclear reference standard (Table 2).

Outcomes

The intent of the AI applications in the included studies varied, with the majority focusing on diagnosing advanced or recurrent cancer (n=6 studies) and four studies classifying the pathology (i.e., normal or abnormal, or benign or malignant) (online supplementary Table 1). Diagnostic performance of the AI models ranged between 70.0 and 95.0% sensitivity, and 52.9% and 98.0% specificity (online supplementary Table 1). The reporting of the diagnostic measures of accuracy were unstandardised,

For peer review only

for example, there was different output measures across the studies and three studies did not report all of their outcome measures (online supplementary Table 1).

Following model development with a training and tuning set, four studies had an external validation test set and compared the performance of the AI model with a human comparator (a radiologist) (online supplementary Table 1) [32,33,39,40]. The diagnostic performance of the radiologists ranged between 57.4% and 62.8% sensitivity and 0.89 and 0.97 AUC (online supplementary Table 1). In these studies, there was variability in both the number of patients (range 50–414; from one to six different centres) and number of radiologists (range 2–4) and their years of experience. These studies reported the diagnostic performance of the AI model as either superior (n=2; in rectal and advanced gastrointestinal cancer) [39,40] or comparable (n=2; in metastatic gastrointestinal pathology and a gynaecology study distinguishing between malignant tumours and benign pathology) to radiologists [32,33]. Two of these studies included a comparison of the interpretation time of the AI model with the radiologists [32,40]. AI models outperformed the radiologists in both studies (1–2 seconds vs 200 seconds per case, p<0.05 [40] and 20s vs 600s for an average of 100 MR images [32]).

Risk of bias and applicability

With the exception of one study [40], all the included studies had an overall judgement of “at risk of bias” and “concerns regarding applicability” (Table 3). This was predominantly due to comparisons based on either a single clinician’s assessment (n=10) or an unclear number of clinicians for the assessment (n=5), from either a small (e.g., 10 patients in one study) or unclear size of test set and most models were

For peer review only

developed only from internal validations (n=11) (Table 2 and online supplementary Table 1).

For peer review only

Table 3 Study quality assessment (QUADAS-2 tool)

First author (reference)	RISK OF BIAS				APPLICABILITY CONCERNS		
	PATIENT SELECTION	INDEX TEST	REFERENCE STANDARD	FLOW AND TIMING	PATIENT SELECTION	INDEX TEST	REFERENCE STANDARD
Acar [25]	Low	Low	High	Low	Low	Low	High
Coy [26]	Low	Low	High	Low	Low	Low	High
Han [32]	Unclear	Low	High	Low	Low	Low	High
Kawauchi [33]	Low	Low	High	Low	Low	Low	High
Lu [36]	Unclear	Low	High	Low	Low	Low	High
Nakagawa [37]	Low	Low	High	Low	Low	Low	High
Koizumi [34]	Low	Low	High	Low	Low	Low	High
Lee [35]	Low	Low	High	Low	Low	Low	High
Nayak [38]	Unclear	Low	Unclear	Low	Low	Low	High
Oberai [39]	Low	Low	High	Low	Low	Low	High
Yasaka [29]	Unclear	Low	High	Low	Low	Low	High
Saiprasad [27]	Unclear	Low	High	Low	Low	Low	High
Sethi [28]	High	Low	Unclear	Low	Low	Low	Unclear
Yuan [30]	Low	Low	High	Low	Low	Low	High
Zhao [31]	Low	Low	Low	Low	Low	Low	Low

For peer review only

DISCUSSION

The major finding of this review was the heterogeneity in the AI applications across the included studies regardless of the surgical speciality or pathology. Early phase studies of AI innovation, particularly focusing on advanced or recurrent malignancy, were identified with promising diagnostic accuracies to support clinical decision making. Future AI research could benefit from targeting areas where radiological expertise is in high demand or the data is complex to interpret; for example, adrenal incidentalomas [41] and images from virtual colonoscopy [2]. Attention should also be directed to the governance of AI, particularly on where the responsibility lies if the AI model misses a lesion.

In this review, several reporting issues were identified, including for the reference standard and training data. Poor adherence to reporting guidelines is a common finding in the existing literature for diagnostic accuracy studies assessing AI interventions [4,11]. The Standards for Reporting of Diagnostic Accuracy-AI (STARD-AI) Steering Group are developing an AI-specific extension to the STARD statement, which aims to improve reporting of AI diagnostic accuracy studies [42]. This steering group highlighted three pitfalls, which are also reflected in this review: (1) unclear methodological interpretation (e.g. methods of validation and comparison to human performance), (2) unstandardised nomenclature (e.g. varying definition of the term ‘validation’), and (3) heterogeneity of the outcome measures (e.g. sensitivity, specificity, predictive values and AUC) [43]. Endeavours to address this include the development of specific reporting guidelines for authors of AI studies, including protocols (SPIRIT-AI) [44], reports (CONSORT-AI) [45] and proposals (MINIMAR)

For peer review only

[46]. These efforts should improve both the reporting quality and make it easier to interpret and compare AI studies.

A minority of the included studies compared the diagnostic performance of the AI model with a clinician's diagnosis. These studies reported a faster and superior or equivalent diagnostic performance with the AI model. A recent review found only 51 studies worldwide reporting the implementation and evaluation of AI applications in clinical practice [47]. While many AI studies are currently retrospective and proof-of-concept [10,11,47], which may be appropriate for early phase surgical research, future efforts should evaluate the role of AI in a clinical setting. This should adopt a multidisciplinary team of all relevant stakeholders (e.g., software engineers, radiologists, and surgeons) to ensure the diverse and relevant skills sets can work together to produce both high-quality and clinically relevant AI research.

This review included a robust methodology, comprehensive search strategy and a multidisciplinary team. Some limitations, however, are acknowledged. Despite having a broad search strategy, relevant studies may have been missed by excluding articles that were not published in the English language. It did not encompass all diagnostic applications of AI in this region, such as diagnostic imaging for prognostic, surveillance, and management decisions meaning findings are not generalisable to these wider contexts. However, recommendations for prioritising future endeavours on clinical need, adhering to reporting guidelines and standardised and transparent reporting can be considered appropriate for all studies assessing AI interventions in healthcare.

For peer review only

This review identified a diverse application of AI innovation in this field. Most studies were proof-of-concept and more 'comparator' studies in the clinical setting are needed. Future AI research could build on existing studies with translation to clinical practice, adopting a multidisciplinary approach, including patient and public involvement, which was lacking in the studies of this review. This could target areas of clinical need. Adherence to new and developing guidelines for reporting AI studies, such as SPIRIT-AI, CONSORT-AI and STARD-AI, are warranted.

For peer review only

ETHICS STATEMENT

Ethical approval was not required as no primary data were collected.

CONTRIBUTORSHIP STATEMENT

GEF and NSB conceived the idea for this systematic review. All authors (GEF, NSB, CH, MPC, NJS and RCM) contributed to the design of the study. GEF and CH performed screening and data extraction. GEF and RCM conducted the QUADAS-2 assessments. All authors (GEF, NSB, CH, MPC, NJS and RCM) contributed towards the data analysis and interpretation of the data. GEF drafted the manuscript (guarantor of review) and all authors (GEF, NSB, CH, MPC, NJS and RCM) were involved in critiquing the manuscript. All authors approved the final manuscript before submission.

COMPETING INTERESTS

The authors have no conflicts of interest to declare.

FUNDING

NSB is funded by an MRC Clinical Scientist Award (grant number: MR/S001751/1). This study was supported by the National Institute for Health and Care Research Bristol Biomedical Research Centre. The views expressed are those of the author(s) and not necessarily those of the NIHR or the Department of Health and Social Care.

DATA SHARING AGREEMENT

All data relevant to the study are included in the article or uploaded as supplementary information.

For peer review only

FIGURE LEGEND/CAPTIONS

(1) Table 1 Characteristics of the included studies

Abbreviations: CT (computer tomography), PET (positron emission tomography) and MR (magnetic resonance).

(2) Table 2 Study details and AI models

Abbreviations: Artificial neural network (ANN), Computerized tomography (CT), convolutional neural network (CNN), k-nearest neighbours algorithm (KNN), magnetic resonance (MR), positron emission tomography (PET), random forest classification (RFC) and support vector machine (SVM).

(3) Table 3 Study quality assessment (QUADAS-2 tool)

(4) Figure 1 PRISMA flow diagram

(5) Online supplementary Table 1 Performance of AI models

Abbreviations: Artificial neural network (ANN), area under the curve (AUC), benign oncocytoma (ONC), clear cell renal cell carcinoma (ccRCC), CNN (convolutional neural network), computer tomography (CT), Ext (external data set), hepatocellular carcinoma (HCC), Int (internal data set), machine learning (ML), k-nearest neighbours algorithm (KNN), multiparametric magnetic resonance imaging (mpMRI), negative predictive value (NPV), positive predictive value (PPV), positron emission tomography (PET), random forest classification (RFC) and support vector machine (SVM).

For peer review only

References

1 Oren O, Gersh BJ, Bhatt DL. Artificial intelligence in medical imaging: switching from radiographic pathological data to clinically meaningful endpoints. *Lancet Digit Health* 2020;**2**:e486–8. doi:10.1016/S2589-7500(20)30160-6

2 Hosny A, Parmar C, Quackenbush J, *et al.* Artificial intelligence in radiology. *Nat Rev Cancer* 2018;**18**:500–10. doi:10.1038/s41568-018-0016-5.Artificial

3 He J, Baxter SL, Xu J, *et al.* The practical implementation of artificial intelligence technologies in medicine. *Nat Med* 2019;**25**:30–6. doi:10.1038/s41591-018-0307-0

4 Aggarwal R, Sounderajah V, Martin G, *et al.* Diagnostic accuracy of deep learning in medical imaging: a systematic review and meta-analysis. *NPJ Digit Med* 2021;**4**. doi:10.1038/s41746-021-00438-z

5 Jin P, Ji X, Kang W, *et al.* Artificial intelligence in gastric cancer: a systematic review. *J Cancer Res Clin Oncol* 2020;**146**:2339–50. doi:10.1007/s00432-020-03304-9

6 Senders JT, Arnaout O, Karhade A v., *et al.* Natural and artificial intelligence in neurosurgery: A systematic review. *Clin Neurosurg* 2018;**83**:181–92. doi:10.1093/neuros/nyx384

7 Raffort J, Adam C, Carrier M, *et al.* Artificial intelligence in abdominal aortic aneurysm. *J Vasc Surg* 2020;**72**:321-333.e1. doi:10.1016/j.jvs.2019.12.026

8 Esteva A, Kuprel B, Novoa RA, *et al.* Dermatologist-level classification of skin cancer with deep neural networks. *Nature* 2017;**542**:115–8. doi:10.1038/nature21056

9 Lin H, Li R, Liu Z, *et al.* Diagnostic Efficacy and Therapeutic Decision-making Capacity of an Artificial Intelligence Platform for Childhood Cataracts in Eye Clinics: A Multicentre Randomized Controlled Trial. *EClinicalMedicine* 2019;**9**:52–9. doi:10.1016/j.eclinm.2019.03.001

10 Ben-Israel D, Jacobs WB, Casha S, *et al.* The impact of machine learning on patient care: A systematic review. *Artif Intell Med* 2020;**103**:101785. doi:10.1016/j.artmed.2019.101785

11 Nagendran M, Chen Y, Lovejoy CA, *et al.* Artificial intelligence versus clinicians: Systematic review of design, reporting standards, and claims of deep learning studies in medical imaging. *The BMJ* 2020;**368**:1–12. doi:10.1136/bmj.m689

12 Yusuf M, Atal I, Li J, *et al.* Reporting quality of studies using machine learning models for medical diagnosis: a systematic review. *BMJ Open* 2020;**10**:e034568. doi:10.1136/bmjopen-2019-034568

13 Fowler GE, Macefield RC, Hardacre C, *et al.* Artificial intelligence as a diagnostic aid in cross-sectional radiological imaging of the abdominopelvic cavity: a protocol for a systematic review. *BMJ Open* 2021;**11**:e054411. doi:10.1136/bmjopen-2021-054411

14 McInnes MDF, Moher D, Thombs BD, *et al.* Preferred Reporting Items for a Systematic Review and Meta-analysis of Diagnostic Test Accuracy Studies The PRISMA-DTA Statement. *JAMA - Journal of the American Medical Association* 2018;**319**:388–96. doi:10.1001/jama.2017.19163

15 Liu X, Faes L, Kale AU, *et al.* A comparison of deep learning performance against health-care professionals in detecting diseases from medical imaging:

For peer review only

a systematic review and meta-analysis. *Lancet Digit Health* 2019;**1**:e271–97. doi:10.1016/S2589-7500(19)30123-2
16 Yang Y, Jin G, Pang Y, *et al.* The diagnostic accuracy of artificial intelligence in thoracic diseases. *Medicine* 2020;**99**:e19114. doi:10.1097/md.0000000000019114
17 Ouzzani M, Hammady H, Fedorowicz Z, *et al.* Rayyan-a web and mobile app for systematic reviews. *Syst Rev* 2016;**5**:1–10. doi:10.1186/s13643-016-0384-4
18 JJ, Deeks, S, Wisniewski DC, Diagnostic C, Accuracy T, *et al.* Guide to the contents of a Cochrane Diagnostic Test Accuracy Protocol. *Cochrane Handbook for Systematic Reviews of Diagnostic Test Accuracy Version 100 The Cochrane Collaboration* 2013;:1–15.
19 Hassan C, Spadaccini M, Iannone A, *et al.* Performance of artificial intelligence for colonoscopy regarding adenoma and polyp detection: a meta-analysis. *Gastrointest Endosc* Published Online First: 2020. doi:10.1016/j.gie.2020.06.059
20 Lui TKL, Tsui VWM, Leung WK. Accuracy of artificial intelligence–assisted detection of upper GI lesions: a systematic review and meta-analysis. *Gastrointest Endosc* 2020;**92**:821-830.e9. doi:10.1016/j.gie.2020.06.034
21 Cousins S, Blencowe NS, Blazeby JM. What is an invasive procedure? A definition to inform study design, evidence synthesis and research tracking. *BMJ Open* 2019;**9**:2018–20. doi:10.1136/bmjopen-2018-028576
22 Harris PA, Taylor R, Minor BL, *et al.* The REDCap consortium: Building an international community of software platform partners. *J Biomed Inform* 2019;**95**:103208. doi:10.1016/j.jbi.2019.103208
23 Whiting PF, Rutjes AW, Westwood ME, Mallett S, Deeks JJ, Reitsma JB, Leeflang MM, Sterne JA BP. QUADAS-2 Group. QUADAS-2: a revised tool for the quality assessment of diagnostic accuracy studies. *Ann Intern Med* 2011;**8**:29–36. doi:0.7326/0003-4819-155-8-201110180-00009. PMID: 22007046.
24 Viknesh Sounderajah, Hutan Ashrafian, Sherri Rose, Nigam H. Shah, Marzyeh Ghassemi, Robert Golub, Charles E. Kahn Jr, Andre Esteva, Alan Karthikesalingam, Bilal Mateen, Dale Webster, Dan Milea, Daniel Ting, Darren Treanor, Dominic Cushnan, Dominic King, D AD. A quality assessment tool for artificial intelligence-centered diagnostic test accuracy studies: QUADAS-AI. *Nat Med* 2021;**27**:1663–5. doi:10.1038/s41591-021-01494-4
25 Campbell M, McKenzie JE, Sowden A, *et al.* Synthesis without meta-analysis (SWiM) in systematic reviews: Reporting guideline. *The BMJ* 2020;**368**:1–6. doi:10.1136/bmj.l6890
26 Acar E, Leblebici A, Ellidokuz BE, *et al.* Full paper: Machine learning for differentiating metastatic and completely responded sclerotic bone lesion in prostate cancer: A retrospective radiomics study. *British Journal of Radiology* 2019;**92**. doi:http://dx.doi.org/10.1259/bjr.20190286
27 Coy H, Hsieh K, Wu W, *et al.* Deep learning and radiomics: the utility of Google TensorFlow™ Inception in classifying clear cell renal cell carcinoma and oncocytoma on multiphasic CT. *Abdominal Radiology* 2019;**44**:2009–20. doi:10.1007/s00261-019-01929-0
28 Han S, Hwang S Il, Lee HJ. The Classification of Renal Cancer in 3-Phase CT Images Using a Deep Learning Method. *J Digit Imaging* 2019;**32**:638–43. doi:10.1007/s10278-019-00230-2

For peer review only - http://bmjopen.bmj.com/site/about/guidelines.xhtml

29 Kawauchi K, Furuya S, Hirata K, *et al.* A convolutional neural network-based system to classify patients using FDG PET/CT examinations. *BMC Cancer* 2020;**20**:227. doi:https://dx.doi.org/10.1186/s12885-020-6694-x
30 Koizumi M, Motegi K, Koyama M, *et al.* Diagnostic performance of a computer-assisted diagnosis system for bone scintigraphy of newly developed skeletal metastasis in prostate cancer patients: search for low-sensitivity subgroups. *Ann Nucl Med* 2017;**31**:521–8. doi:http://dx.doi.org/10.1007/s12149-017-1175-2
31 Lee JJ, Yang H, Franc BL, *et al.* Deep learning detection of prostate cancer recurrence with 18F-FACBC (fluciclovine, Axumin®) positron emission tomography. *Eur J Nucl Med Mol Imaging* 2020;**47**:2992–7. doi:10.1007/s00259-020-04912-w
32 Lu Y, Yu Q, Gao Y, *et al.* Identification of metastatic lymph nodes in MR imaging with faster region-based convolutional neural networks. *Cancer Res* 2018;**78**:5135–43. doi:10.1158/0008-5472.CAN-18-0494
33 Nakagawa M, Nakaura T, Namimoto T, *et al.* A multiparametric MRI-based machine learning to distinguish between uterine sarcoma and benign leiomyoma: comparison with 18F-FDG PET/CT. *Clin Radiol* 2019;**74**:167.e1-167.e7. doi:10.1016/j.crad.2018.10.010
34 Nayak A, Baidya Kayal E, Arya M, *et al.* Computer-aided diagnosis of cirrhosis and hepatocellular carcinoma using multi-phase abdomen CT. *Int J Comput Assist Radiol Surg* 2019;**14**:1341–52. doi:http://dx.doi.org/10.1007/s11548-019-01991-5
35 Oberai A, Varghese B, Cen S, *et al.* Deep learning based classification of solid lipid-poor contrast enhancing renal masses using contrast enhanced CT. *Br J Radiol* 2020;**93**:20200002. doi:http://dx.doi.org/10.1259/bjr.20200002
36 Saiprasad G, Chang CI, Safdar N, *et al.* Adrenal gland abnormality detection using random forest classification. *J Digit Imaging* 2013;**26**:891–7. doi:http://dx.doi.org/10.1007/s10278-012-9554-7
37 Sethi G, Saini BS. Computer aided diagnosis system for abdomen diseases in computed tomography images. *Biocybern Biomed Eng* 2016;**36**:42–55. doi:10.1016/j.bbe.2015.10.008
38 Yasaka K, Akai H, Abe O, *et al.* Deep learning with CNN showed high diagnostic performance in differentiation of liver masses at dynamic CT. *Radiology* 2018;**286**:887–96.
39 Yuan Z, Xu T, Cai J, *et al.* Development and Validation of an Image-based Deep Learning Algorithm for Detection of Synchronous Peritoneal Carcinomatosis in Colorectal Cancer. *Ann Surg* 2020;**16**. doi:http://dx.doi.org/10.1097/SLA.0000000000004229
40 Zhao X, Xie P, Wang M, *et al.* Deep learning-based fully automated detection and segmentation of lymph nodes on multiparametric-mri for rectal cancer: A multicentre study. *EBioMedicine* 2020;**56**:102780. doi:https://dx.doi.org/10.1016/j.ebiom.2020.102780
41 Garrett RW, Nepute JC, El Hayek M, *et al.* Adrenal Incidentalomas: Clinical controversies and modified recommendations. *American Journal of Roentgenology* 2016;**206**:1170–8. doi:10.2214/AJR.15.15475
42 Sounderajah V, Ashrafian H, Karthikesalingham A, Marker SR, Normanhani P, Collins GS, Bossuyt PM DD. Developing Specific Reporting Standards in Artificial Intelligence Centred Research Viknesh. *Ann Surg* 2021;:6. doi:10.1097/SLA.0000000000005294

43 Sounderajah V, Ashrafian H, Aggarwal R, *et al.* Developing specific reporting guidelines for diagnostic accuracy studies assessing AI interventions: The STARD-AI Steering Group. *Nat Med* 2020;**26**:807–8. doi:10.1038/s41591-020-0941-1

44 Cruz Rivera S, Liu X, Chan AW, *et al.* Guidelines for clinical trial protocols for interventions involving artificial intelligence: the SPIRIT-AI extension. *Nat Med* 2020;**26**:1351–63. doi:10.1038/s41591-020-1037-7

45 Liu X, Cruz Rivera S, Moher D, *et al.* Reporting guidelines for clinical trial reports for interventions involving artificial intelligence: the CONSORT-AI extension. *Nat Med* 2020;**26**:1364–74. doi:10.1038/s41591-020-1034-x

46 Hernandez-Boussard T, Bozkurt S, Ioannidis JPA, *et al.* MINIMAR (MINimum Information for Medical AI Reporting): Developing reporting standards for artificial intelligence in health care. *Journal of the American Medical Informatics Association* 2020;**00**:1–5. doi:10.1093/jamia/ocaa088

47 Yin J, Ngiam KY TH. Role of Artificial Intelligence Applications in Real-Life Clinical Practice: Systematic Review. *J Med Internet Res* 2021;**23**:e25759. doi:10.2196/25759

For peer review only

Figure 1 PRISMA flow diagram

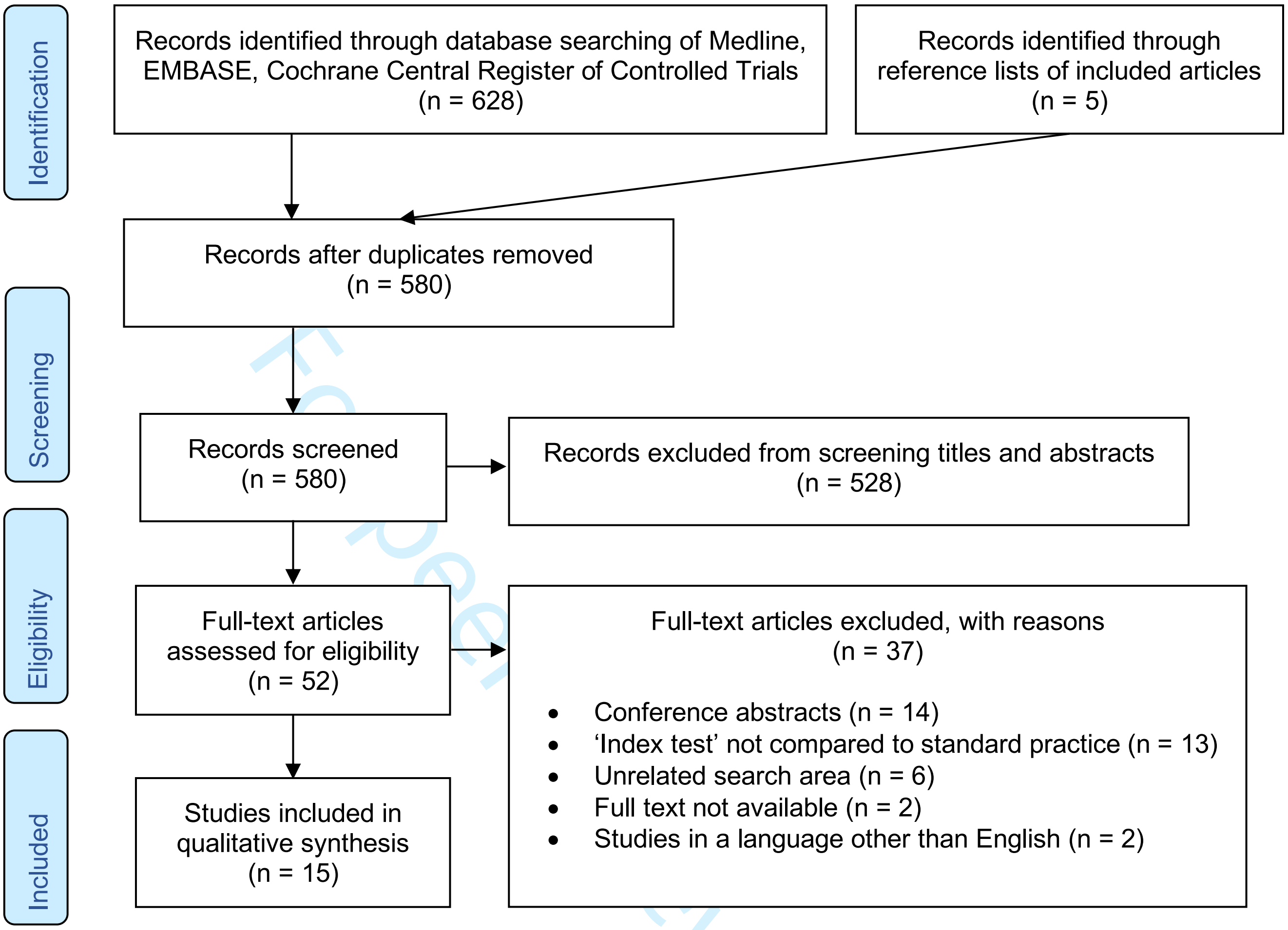

For peer review only

Online supplementary Table 1 Performance of AI models

First author (Reference)	Study aim	Best AI model	Output measures	AI performance	Human comparator	Human performance	External validation
**Acar [25]**	Distinguish the metastatic and completely responded areas regarding sclerotic bone lesions imaged via 68Ga-PSMA PET/CT in patients with known bone metastasis.	KNN	Sensitivity<br>Specificity<br>Predictive values (PPV, NPV)<br>AUC<br>Overall accuracy	73.5%<br>73.7%<br>86.9%, 53.8%<br>76.0%<br>73.5%	NA	NA	No
**Coy [26]**	Investigate the diagnostic value and feasibility of a deep learning-based renal lesion classifier in differentiating ccRCC from ONC on routine four-phase MDCT in patients with pathologically confirmed renal masses.	CNN	Sensitivity<br>Specificity<br>Predictive values (PPV, NPV)<br>Overall accuracy	88.3%<br>52.9%<br>79.6%, 59.5%<br>75.4%	NA	NA	No
**Han [32]**	Develop reproducible and generalizable models for discriminating three major subtypes of RCCs using CT image analysis along with a ML algorithm.	CNN	Sensitivity<br>Specificity<br>AUC<br>Overall accuracy	0.98<br>0.93<br>0.9<br>0.85	NA	NA	No
**Koizumi [34]**	Investigate the diagnostic performance of computer-assisted diagnostic system for bone scintigraphy in prostate cancer patients with and without skeletal metastasis.	ANN	Sensitivity<br>Specificity<br>AUC	82.0%<br>83.0%<br>0.89	NA	NA	No
**Lee [35]**	Evaluate the performance of deep learning classifiers in discriminating normal and abnormal 18F-FACBC PET scans based on the presence of tumour recurrence and/or metastases in patients with	CNN	Sensitivity<br>Specificity<br>AUC	90.7%<br>95.1%<br>0.97	NA	NA	No

	prostate cancer and biochemical recurrence.						
**Oberai [39]**	Classify lipid poor enhancing renal masses into 'benign' and 'malignant' categories.	CNN	Sensitivity<br>Specificity<br>AUC<br>Overall accuracy	70.0%<br>81.0%<br>0.82<br>78.0%	NA	NA	No
**Lu [36]**	To develop an assistive tool to aid radiologists to more effectively and accurately determine lymph node metastases.	CNN	Sensitivity<br>Specificity<br>AUC<br>Overall accuracy	Not reported<br>Not reported<br>0.91<br>99.9%	Radiologists (number not specified)	Values not explicitly reported.	Yes
**Nayak [38]**	Study proposes a computer-aided diagnosis system for detecting cirrhosis and HCC.	SVM	Prediction accuracy	80.8 - 86.9%	NA	NA	No
**Sethi [28]**	Paper proposed automated CAD system for classification of abdomen diseases like tumour, cyst, stone and normal tissues from CT images of abdomen.	ANN	Sensitivity<br>Specificity<br>Predictive values (PPV, NPV)<br>AUC<br>Overall accuracy	95.0%<br>98.0%<br>No raw data<br>No raw data<br>95.1%	NA	NA	No
**Yasaka [29]**	Investigate diagnostic performance by using a deep learning method with a CNN for the differentiation of liver masses at dynamic contrast agent-enhanced CT.	CNN	Sensitivity<br>Specificity<br>AUC<br>Overall accuracy	0.33 - 1.00<br>Not reported<br>0.92<br>0.84	NA	NA	No
**Yuan [30]**	Develop a CNN-based ResNet3d algorithm and SVM classified to detect synchronous peritoneal carcinomatosis in colorectal cancer.	CNN + SVM combination	Sensitivity<br>Specificity<br>Predictive values (PPV, NPV)<br>AUC<br>Overall accuracy	93.8%<br>94.4%<br>93.8%, 94.4%<br>0.92<br>94.1%	2 Radiologists (senior and junior)	57.4%, 58.1%<br>100%, 98%<br>No raw data<br>0.79, 0.78<br>No raw data	Yes
**Zhao [31]**	Develop and validate a deep-learning-based, fully automated lymph node detection and segmentation model based on mpMRI.	CNN	Sensitivity<br><br>Predictive values (PPV, NPV)	80.0%<br><br>73.5%, NA	4 Radiologists	38.2% (Int), 62.6% (Ext)<br>43.8% (Int), 35.9% (Ext)	Yes
**Saiprasad [27]**	Identify adrenal glands from a volumetric CT acquisition and	RFC	Sensitivity<br>Specificity	80.0%<br>90.0%	NA	NA	No

	determine whether they are normal or abnormal.						
**Nakagawa [37]**	Compare the performance of ML using mp-MRI and PET to distinguish between uterine sarcoma and leiomyoma.	Logistic regression (multivariate)	AUC	0.92	2 Radiologists (11 and 20 years of experience)	0.97 and 0.89	Yes
**Kawauchi [33]**	Develop a CNN-based system that can classify whole-body FDG PET as 1) benign, 2) malignant or 3) equivocal.	CNN	Prediction	91.0% - 94.9% (abdomen) 95.3% - 99.7% (pelvic region)	NA	NA	No

Abbreviations: Artificial neural network (ANN), area under the curve (AUC), benign oncocytoma (ONC), clear cell renal cell carcinoma (ccRCC), CNN (convolutional neural network), computer tomography (CT), Ext (external data set), hepatocellular carcinoma (HCC), Int (internal data set), machine learning (ML), k-nearest neighbours algorithm (KNN), multiparametric magnetic resonance imaging (mpMRI), negative predictive value (NPV), positive predictive value (PPV), positron emission tomography (PET), random forest classification (RFC) and support vector machine (SVM).

.

For peer review only

APPENDIX S1

Artificial intelligence as a diagnostic aid in cross-sectional radiological imaging of surgical pathology in the abdominopelvic cavity: A systematic review

Medline Search

1. artificial intelligence/ or machine learning/ or deep learning/ or supervised machine learning/ or support vector machine/ or unsupervised machine learning/
2. computer heuristics/ or expert systems/ or fuzzy logic/ or knowledge bases/ or biological ontologies/ or gene ontology/ or natural language processing/ or neural networks, computer/ or robotics/
3. Machine learning.ti,ab,kf.
4. Artificial Intelligence.ti,ab,kf.
5. Naive Bayes.ti,ab,kf.
6. bayesian learning.ti,ab,kf.
7. Neural network*.ti,ab,kf.
8. Natural language processing.ti,ab,kf.
9. support vector*.ti,ab,kf.
10. random forest*.ti,ab,kf.
11. boosting.ti,ab,kf.
12. deep learning.ti,ab,kf.
13. machine intelligence.ti,ab,kf.
14. computational intelligence.ti,ab,kf.
15. computer reasoning.ti,ab,kf.
16. 1 or 2 or 3 or 4 or 5 or 6 or 7 or 8 or 9 or 10 or 11 or 12 or 13 or 14 or 15

For peer review only

17. tomography/ or colonography, computed tomographic/ or computed tomography angiography/ or positron emission tomography computed tomography/

18. magnetic resonance imaging/ or cholangiopancreatography, magnetic resonance/ or diffusion magnetic resonance imaging/ or diffusion tensor imaging/ or echo-planar imaging/ or fluorine-19 magnetic resonance imaging/ or magnetic resonance angiography/ or magnetic resonance imaging, cine/ or multiparametric magnetic resonance imaging/

19. (MRI or Magnetic Resonance Imaging).ti,ab,kf.

20. 17 or 18 or 19

21. pelvis/ or lesser pelvis/ or pelvic floor/

22. (radiography adj3 abdominal).ti,ab,kf.

23. (Abdomen or abdominal cavity or peritoneum or douglas' pouch or mesentery or mesocolon or omentum or peritoneal cavity or peritoneal stomata or retroperitoneal space).ti,ab,kf.

24. (Pelvis or pelvic floor).ti,ab,kf.

25. 21 or 22 or 23 or 24

26. exp Animals/ not Humans/

27. (animal model* or rat or rats or mouse or mice or rodent* or sheep or lambs or murine or pigs or piglets or swine or porcine or rabbit or rabbits or cat or cats or feline or dog or dogs or canine or cattle or bovine or marmoset* or monkey or monkeys or trout or zebra fish*).ti.

28. (Comment or editorial or letter or case reports).pt.

29. (endoscope or gastroscope or colonoscope or capsule endoscopy or endoscopy).ti,ab,kf.

30. 26 or 27 or 28 or 29

For peer review only

31. 16 and 20 and 25

32. 31 not 30

33. (2012* or 2013* or 2014* or 2015* or 2016* or 2017* or 2018* or 2019* or 2020* or 202101*).yr,ed,dc,ep.

34. 32 and 33

For peer review only

Embase via Ovid

1. artificial intelligence/ or ambient intelligence/ or automated reasoning/ or computer heuristics/ or multicriteria decision analysis/

2. exp machine learning/

3. deep learning/

4. supervised machine learning/

5. exp support vector machine/

6. unsupervised machine learning/

7. hyperheuristics/ or metaheuristics/

8. expert systems/ or fuzzy logic/ or knowledge bases/ or natural language processing/ or neural networks, computer/ or robotics/

9. biological ontologies/ or gene ontology/

10. ((machine or bayesian or deep) adj1 learning).ti,ab,kw.

11. ((artificial or machine or computational) adj1 intelligence).ti,ab,kw.

12. Naive Bayes.ti,ab,kw.

13. Neural network*.ti,ab,kw.

14. Natural language processing.ti,ab,kw.

15. support vector*.ti,ab,kw.

16. random forest*.ti,ab,kw.

17. boosting.ti,ab,kw.

18. computer reasoning.ti,ab,kw.

19. 1 or 2 or 3 or 4 or 5 or 6 or 7 or 8 or 9 or 10 or 11 or 12 or 13 or 14 or 15 or 16 or 17 or 18

20. exp Tomography/

For peer review only

21. colonography, computed tomographic/ or computed tomography angiography/ or four-dimensional computed tomography/ or positron emission tomography computed tomography/ or single photon emission computed tomography computed tomography/ or tomography, spiral computed/

22. magnetic resonance imaging/ or cholangiopancreatography, magnetic resonance/ or diffusion magnetic resonance imaging/ or diffusion tensor imaging/ or echo-planar imaging/ or fluorine-19 magnetic resonance imaging/ or magnetic resonance angiography/ or magnetic resonance imaging, cine/ or multiparametric magnetic resonance imaging/

23. (MRI or Magnetic Resonance Imaging).ti,ab,kw.

24. 20 or 21 or 22 or 23

25. pelvis/ or lesser pelvis/ or pelvic floor/

26. (radiography adj3 abdominal).ti,ab,kw.

27. (Abdomen or abdominal cavity or peritoneum or douglas' pouch or mesentery or mesocolon or omentum or peritoneal cavity or peritoneal stomata or retroperitoneal space).ti,ab,kw.

28. (Pelvis or pelvic floor).ti,ab,kw.

29. 25 or 26 or 27 or 28

30. (Animal/ or Nonhuman/) not Human/

31. Animal Experiment/ not (Human Experiment/ or Human/)

32. (animal model* or rat or rats or mouse or mice or rodent* or sheep or lambs or murine or pigs or piglets or swine or porcine or rabbit or rabbits or cat or cats or feline or dog or dogs or canine or cattle or bovine or marmoset* or monkey or monkeys or trout or zebra fish*).ti.

33. (Comment or editorial or letter or case reports).pt.

For peer review only

34. (endoscope or gastroscope or colonoscope or capsule endoscopy or endoscopy).ti,ab,kw.

35. 30 or 31 or 32 or 33 or 34

36. 19 and 24 and 29

37. 36 not 35

38. (2012* or 2013* or 2014* or 2015* or 2016* or 2017* or 2018* or 2019* or 2020* or 202101*).yr,em.

39. 37 and 38

For peer review only

Cochrane Central Register of Controlled Trials

#1 MeSH descriptor: [Artificial Intelligence] this term only MeSH

#2 MeSH descriptor: [Machine Learning] this term only MeSH

#3 MeSH descriptor: [Deep Learning] this term only MeSH

#4 MeSH descriptor: [Supervised Machine Learning] this term only MeSH

#5 MeSH descriptor: [Unsupervised Machine Learning] this term only MeSH

#6 MeSH descriptor: [Support Vector Machine] this term only MeSH

#7 MeSH descriptor: [Computer Heuristics] this term only MeSH

#8 MeSH descriptor: [Expert Systems] this term only MeSH

#9 MeSH descriptor: [Fuzzy Logic] this term only MeSH

#10 MeSH descriptor: [Knowledge Bases] this term only MeSH

#11 MeSH descriptor: [Biological Ontologies] this term only MeSH

#12 MeSH descriptor: [Gene Ontology] this term only MeSH

#13 MeSH descriptor: [Natural Language Processing] this term only MeSH

#14 MeSH descriptor: [Neural Networks, Computer] this term only MeSH

#15 MeSH descriptor: [Robotics] this term only MeSH

#16 (machine learning): ti,ab,kw

#17 (artificial intelligence):ti,ab,kw

#18 (naive bayes):ti,ab,kw

#19 (bayesian learning):ti,ab,kw

#20 (neural network*):ti,ab,kw

#21 (natural language processing):ti,ab,kw

#22 (support vector*):ti,ab,kw

#23 (random forest*):ti,ab,kw

#24 (boosting):ti,ab,kw

For peer review only

#25 (deep learning):ti,ab,kw

#26 (machine intelligence):ti,ab,kw

#27 (computational intelligence):ti,ab,kw

#28 (computer reasoning):ti,ab,kw

#29 #1 or #2 or #3 or #4 or #5 or #6 or #7 or #8 or #9 or #10 or #11 or #12 or #13 or #14 or #15 or #16 or #17 or #18 or #19 or #20 or #21 or #22 or #23 or #24 or #25 or #26 or #27 or #28

#30 MeSH descriptor: [Tomography] this term only MeSH

#31 MeSH descriptor: [Colonography, Computed Tomographic] this term only MeSH

#32 MeSH descriptor: [Computed Tomography Angiography] this term only MeSH

#33 MeSH descriptor: [Positron-Emission Tomography] this term only MeSH

#34 MeSH descriptor: [Positron Emission Tomography Computed Tomography] this term only MeSH

#35 MeSH descriptor: [Magnetic Resonance Imaging] this term only MeSH

#36 MeSH descriptor: [Cholangiopancreatography, Magnetic Resonance] this term only MeSH

#37 MeSH descriptor: [Diffusion Magnetic Resonance Imaging] this term only MeSH

#38 MeSH descriptor: [Diffusion Tensor Imaging] this term only MeSH

#39 MeSH descriptor: [Echo-Planar Imaging] this term only MeSH

#40 MeSH descriptor: [Fluorine-19 Magnetic Resonance Imaging] this term only MeSH

#41 MeSH descriptor: [Magnetic Resonance Angiography] this term only MeSH

#42 MeSH descriptor: [Magnetic Resonance Imaging, Cine] this term only MeSH

#43 MeSH descriptor: [Multiparametric Magnetic Resonance Imaging] this term only MeSH

For peer review only

#44 (MRI):ti,ab,kw

#45 (magnetic resonance imaging):ti,ab,kw

#46 #30 or #31 or #32 or #33 or #34 or #35 or #36 or #37 or #38 or #39 or #40 or #41 or #42 or #43 or #44 or #45

#47 MeSH descriptor: [Pelvis] this term only MeSH

#48 MeSH descriptor: [Lesser Pelvis] this term only MeSH

#49 MeSH descriptor: [Pelvic Floor] this term only MeSH

#50 ((radiography NEAR/3 abdominal)):ti,ab,kw

#51 ((radiography NEAR/3 abdomen)):ti,ab,kw

#52 ((Abdomen or abdominal cavity or peritoneum or douglas' pouch or mesentery or mesocolon or omentum or peritoneal cavity or peritoneal stomata or retroperitoneal space)):ti,ab,kw

#53 (pelvis or pelvic floor):ti,ab,kw

#54 #47 or #48 or #49 or #50 or #51 or #52 or #53

#55 #29 and #46 and #54

For peer review only

PRISMA-DTA for Abstracts Checklist

Section/topic	#	PRISMA-DTA for Abstracts Checklist item	Reported on page #
**TITLE and PURPOSE**			
Title	1	Identify the report as a systematic review (+/- meta-analysis) of diagnostic test accuracy (DTA) studies.	1
Objectives	2	Indicate the research question, including components such as participants, index test, and target conditions.	3
**METHODS**			
Eligibility criteria	3	Include study characteristics used as criteria for eligibility.	3
Information sources	4	List the key databases searched and the search dates.	3
Risk of bias & applicability	5	Indicate the methods of assessing risk of bias and applicability.	3
Synthesis of results	A1	Indicate the methods for the data synthesis.	3
**RESULTS**			
Included studies	6	Indicate the number and type of included studies and the participants and relevant characteristics of the studies (including the reference standard).	3
Synthesis of results	7	Include the results for the analysis of diagnostic accuracy, preferably indicating the number of studies and participants. Describe test accuracy including variability; if meta-analysis was done, include summary results and confidence intervals.	3
**DISCUSSION**			
Strengths and limitations	9	Provide a brief summary of the strengths and limitations of the evidence	4
Interpretation	10	Provide a general interpretation of the results and the important implications.	4
**OTHER**			
Funding	11	Indicate the primary source of funding for the review.	24
Registration	12	Provide the registration number and the registry name	4

Adapted From: McInnes MDF, Moher D, Thombs BD, McGrath TA, Bossuyt PM, The PRISMA-DTA Group (2018). Preferred Reporting Items for a Systematic Review and Meta-analysis of Diagnostic Test Accuracy Studies: The PRISMA-DTA Statement. JAMA. 2018 Jan 23;319(4):388-396. doi: 10.1001/jama.2017.19163.

For more information, visit: www.prisma-statement.org.

For peer review only - http://bmjopen.bmj.com/site/about/guidelines.xhtml

PRISMA-DTA Checklist

Section/topic	#	PRISMA-DTA Checklist Item	Reported on page #
**TITLE / ABSTRACT**			
Title	1	Identify the report as a systematic review (+/- meta-analysis) of diagnostic test accuracy (DTA) studies.	1
Abstract	2	Abstract: See PRISMA-DTA for abstracts.	3-4
**INTRODUCTION**			
Rationale	3	Describe the rationale for the review in the context of what is already known.	6-7
Clinical role of index test	D1	State the scientific and clinical background, including the intended use and clinical role of the index test, and if applicable, the rationale for minimally acceptable test accuracy (or minimum difference in accuracy for comparative design).	6-7
Objectives	4	Provide an explicit statement of question(s) being addressed in terms of participants, index test(s), and target condition(s).	9
**METHODS**			
Protocol and registration	5	Indicate if a review protocol exists, if and where it can be accessed (e.g., Web address), and, if available, provide registration information including registration number.	8
Eligibility criteria	6	Specify study characteristics (participants, setting, index test(s), reference standard(s), target condition(s), and study design) and report characteristics (e.g., years considered, language, publication status) used as criteria for eligibility, giving rationale.	9
Information sources	7	Describe all information sources (e.g., databases with dates of coverage, contact with study authors to identify additional studies) in the search and date last searched.	8
Search	8	Present full search strategies for all electronic databases and other sources searched, including any limits used, such that they could be repeated.	8-9
Study selection	9	State the process for selecting studies (i.e., screening, eligibility, included in systematic review, and, if applicable, included in the meta-analysis).	8-9
Data collection process	10	Describe method of data extraction from reports (e.g., piloted forms, independently, in duplicate) and any processes for obtaining and confirming data from investigators.	9-11
Definitions for data extraction	11	Provide definitions used in data extraction and classifications of target condition(s), index test(s), reference standard(s) and other characteristics (e.g. study design, clinical setting).	9-11
Risk of bias and applicability	12	Describe methods used for assessing risk of bias in individual studies and concerns regarding the applicability to the review question.	11
Diagnostic accuracy measures	13	State the principal diagnostic accuracy measure(s) reported (e.g. sensitivity, specificity) and state the unit of assessment (e.g. per-patient, per-lesion).	10-11
Synthesis of results	14	Describe methods of handling data, combining results of studies and describing variability between studies. This could include, but is not limited to: a) handling of multiple definitions of target condition. b) handling of multiple thresholds of test positivity, c) handling multiple index test readers, d) handling of indeterminate test results, e) grouping and comparing tests, f) handling of different reference standards	11

For peer review only - http://bmjopen.bmj.com/site/about/guidelines.xhtml

PRISMA-DTA Checklist

Section/topic	#	PRISMA-DTA Checklist Item	Reported on page #
Meta-analysis	D2	Report the statistical methods used for meta-analyses, if performed.	NA
Additional analyses	16	Describe methods of additional analyses (e.g., sensitivity or subgroup analyses, meta-regression), if done, indicating which were pre-specified.	11
**RESULTS**			
Study selection	17	Provide numbers of studies screened, assessed for eligibility, included in the review (and included in meta-analysis, if applicable) with reasons for exclusions at each stage, ideally with a flow diagram.	13, Figure 1
Study characteristics	18	For each included study provide citations and present key characteristics including: a) participant characteristics (presentation, prior testing), b) clinical setting, c) study design, d) target condition definition, e) index test, f) reference standard, g) sample size, h) funding sources	13
Risk of bias and applicability	19	Present evaluation of risk of bias and concerns regarding applicability for each study.	18-20, Table 3
Results of individual studies	20	For each analysis in each study (e.g. unique combination of index test, reference standard, and positivity threshold) report 2x2 data (TP, FP, FN, TN) with estimates of diagnostic accuracy and confidence intervals, ideally with a forest or receiver operator characteristic (ROC) plot.	NA
Synthesis of results	21	Describe test accuracy, including variability; if meta-analysis was done, include results and confidence intervals.	17-18
Additional analysis	23	Give results of additional analyses, if done (e.g., sensitivity or subgroup analyses, meta-regression; analysis of index test: failure rates, proportion of inconclusive results, adverse events).	NA
**DISCUSSION**			
Summary of evidence	24	Summarize the main findings including the strength of evidence.	21-23
Limitations	25	Discuss limitations from included studies (e.g. risk of bias and concerns regarding applicability) and from the review process (e.g. incomplete retrieval of identified research).	22
Conclusions	26	Provide a general interpretation of the results in the context of other evidence. Discuss implications for future research and clinical practice (e.g. the intended use and clinical role of the index test).	23
**FUNDING**			
Funding	27	For the systematic review, describe the sources of funding and other support and the role of the funders.	24

Adapted From: McInnes MDF, Moher D, Thombs BD, McGrath TA, Bossuyt PM, The PRISMA-DTA Group (2018). Preferred Reporting Items for a Systematic Review and Meta-analysis of Diagnostic Test Accuracy Studies: The PRISMA-DTA Statement. JAMA. 2018 Jan 23;319(4):388-396. doi: 10.1001/jama.2017.19163.

For more information, visit: **www.prisma-statement.org**.

For peer review only - http://bmjopen.bmj.com/site/about/guidelines.xhtml

BMJ Open

Artificial intelligence as a diagnostic aid in cross-sectional radiological imaging of surgical pathology in the abdominopelvic cavity: a systematic review

Journal:	*BMJ Open*
Manuscript ID	bmjopen-2022-064739.R2
Article Type:	Original research
Date Submitted by the Author:	24-Nov-2022

Complete List of Authors:	Fowler, George; NIHR Bristol Biomedical Research Centre, Population Health Sciences, Bristol Medical School. University of Bristol, UK<br>Blencowe, Natalie; NIHR Bristol Biomedical Research Centre, Population Health Sciences, Bristol Medical School. University of Bristol, UK<br>Hardacre, Conor; Bristol Medical School<br>Callaway, Mark; Bristol Royal Infirmary, Department of Clinical Radiology<br>Smart , Neil ; Royal Devon and Exeter NHS Foundation Trust, Exeter Surgical Health Services Research Unit (HeSRU)<br>Macefield, Rhiannon; NIHR Bristol Biomedical Research Centre, Population Health Sciences, Bristol Medical School. University of Bristol, UK

**Primary Subject Heading**:	Surgery
Secondary Subject Heading:	Diagnostics
Keywords:	SURGERY, Diagnostic radiology < RADIOLOGY & IMAGING, Adult surgery < SURGERY

SCHOLARONE™
Manuscripts

I, the Submitting Author has the right to grant and does grant on behalf of all authors of the Work (as defined in the below author licence), an exclusive licence and/or a non-exclusive licence for contributions from authors who are: i) UK Crown employees; ii) where BMJ has agreed a CC-BY licence shall apply, and/or iii) in accordance with the terms applicable for US Federal Government officers or employees acting as part of their official duties; on a worldwide, perpetual, irrevocable, royalty-free basis to BMJ Publishing Group Ltd ("BMJ") its licensees and where the relevant Journal is co-owned by BMJ to the co-owners of the Journal, to publish the Work in this journal and any other BMJ products and to exploit all rights, as set out in our licence.

The Submitting Author accepts and understands that any supply made under these terms is made by BMJ to the Submitting Author unless you are acting as an employee on behalf of your employer or a postgraduate student of an affiliated institution which is paying any applicable article publishing charge ("APC") for Open Access articles. Where the Submitting Author wishes to make the Work available on an Open Access basis (and intends to pay the relevant APC), the terms of reuse of such Open Access shall be governed by a Creative Commons licence – details of these licences and which Creative Commons licence will apply to this Work are set out in our licence referred to above.

Other than as permitted in any relevant BMJ Author's Self Archiving Policies, I confirm this Work has not been accepted for publication elsewhere, is not being considered for publication elsewhere and does not duplicate material already published. I confirm all authors consent to publication of this Work and authorise the granting of this licence.

For peer review only

Artificial intelligence as a diagnostic aid in cross-sectional radiological imaging of surgical pathology in the abdominopelvic cavity: a systematic review

Authors: George E Fowler[1]
george.fowler@bristol.ac.uk

Natalie S Blencowe[1]
natalie.blencowe@bristol.ac.uk

Conor Hardacre[2]
ch17008@bristol.ac.uk

Mark P Callaway[3]
mark.callaway@uhbw.nhs.uk

Neil J Smart[4]
drneilsmart@hotmail.com

Rhiannon C Macefield[1]
r.macefield@bristol.ac.uk

Affiliations: [1]NIHR Bristol Biomedical Research Centre, Population Health Sciences, Bristol Medical School. University of Bristol, UK.
[2]Bristol Medical School, University of Bristol, UK.
[3]Department of Clinical Radiology, University Hospital Bristol and Weston NHS Foundation Trust, Bristol, UK.
[4]Exeter Surgical Health Services Research Unit (HeSRU), Royal Devon and Exeter Hospital, Exeter, UK.

Correspondence to: George E Fowler

For peer review only

NIHR Bristol Biomedical Research Centre, Population Health Sciences, Bristol Medical School. University of Bristol, UK
george.fowler@bristol.ac.uk

ORCID ID(s):
GEF (0000-0002-4133-802X)
RCM (0000-0002-6606-5427)
CH (0000-0001-9800-0479)
MPC NA
NJS (0000-0002-3043-8324)
NSB (0000-0002-6111-2175)

Keywords: Artificial intelligence; deep learning; systematic review; surgery; diagnostic

Word count: 2,545 words

For peer review only

Abstract

Objectives

There is emerging use of artificial intelligence (AI) models to aid diagnostic imaging. This review examined and critically appraised the application of AI models to identify surgical pathology from radiological images of the abdominopelvic cavity, to identify current limitations and inform future research.

Design

Systematic review.

Data sources

Systematic database searches (Medline, EMBASE, Cochrane Central Register of Controlled Trials) were performed. Date limitations (January 2012-July 2021) were applied.

Eligibility criteria

Primary research studies were considered for eligibility using the PIRT (participants, index test(s), reference standard and target condition) framework. Only publications in the English language were eligible for inclusion in the review.

Data extraction and synthesis

Study characteristics, descriptions of AI models and outcomes assessing diagnostic performance were extracted by independent reviewers. A narrative synthesis was performed in accordance with the Synthesis Without Meta-analysis guidelines. Risk of bias was assessed (QUADAS-2).

Results

Fifteen retrospective studies were included. Studies were diverse in surgical speciality, the intention of the AI applications and the models used. AI training and test sets comprised a median of 130 (range: 5–2440) and 37 (range: 10–1045) patients

For peer review only

respectively. Diagnostic performance of models varied (range: 70-95% sensitivity, 53%–98% specificity). Only four studies compared the AI model with human performance. Reporting of studies was unstandardised and often lacking in detail. Most studies (n=14) were judged as having overall high risk of bias with concerns regarding applicability.

Conclusions

AI application in this field is diverse. Adherence to reporting guidelines is warranted. With finite healthcare resources, future endeavours may benefit from targeting areas where radiological expertise is in high demand to provide greater efficiency in clinical care. Translation to clinical practice and adoption of a multidisciplinary approach should be of high priority.

PROSPERO registration number: CRD42021237249.

For peer review only

Strengths and limitations of this study

- This systematic review examined and critically appraised the application of artificial intelligence models to identify surgical pathology from cross-sectional radiological images, including CT, MR, CT-PET and bone scans, and identified current limitations and evidence gaps, and thereby focusing future research efforts.
- Robust methodology was undertaken including screening, data extraction and of risk of bias assessment by two independent reviewers.
- Findings may be limited by including English language publications only and incomplete reporting in some of the included studies.

For peer review only

INTRODUCTION

The widespread adoption of digital healthcare provides vast data to enable the application of artificial intelligence (AI) in pattern recognition [1]. This can alleviate the burden of, or replace, tasks traditionally dependent on clinicians. Examples include the interpretation of medical images for diagnostic, prognostic, surveillance, and management decisions, which otherwise rely on a limited number of interpreters and human resources [2]. There has been a surge of research into the use of AI in diagnostic imaging, exploring how it can support clinicians and provide greater efficacy and efficiency in clinical care [3].

Systematic reviews on the diagnostic accuracy of AI in medical imaging (including respiratory medicine, ophthalmology and breast cancer) [4], gastroenterology [5], neurosurgery [6] and vascular surgery [7] have demonstrated the diverse application of AI models to detect many pathologies. A variety of imaging modalities have been explored (e.g., computer tomography (CT), magnetic resonance (MR) and positron emission tomography (PET)). AI models can demonstrate diagnostic performances equivalent to that of experts [8] and with greater efficiency, for example, in the time taken to diagnose childhood cataracts (5.7 minutes quicker than senior consultants) [9]. However, while AI technologies offer to markedly reduce the clinical workload, “black box” techniques (AI algorithms) can be difficult or impossible to interpret, which can be a barrier to adopting these techniques into clinical practice. Furthermore, many AI studies are proof-of-concept [10] and poorly reported [11,12], including limited details on participants, making it difficult to replicate or interpret the study findings [12].

For peer review only

A systematic review of the diagnostic accuracy of AI models in cross-sectional radiological imaging of the abdominopelvic cavity is lacking [13]. Synthesis of the current AI research in this area could benefit several different surgical specialities which image this region, such as endocrine surgery, gastrointestinal surgery, obstetrics and gynaecology, urology and vascular surgery to guide their clinical decision making. This study aimed to conduct a systematic review to examine and critically appraise the application of AI models to identify surgical pathology from cross-sectional radiological images, including CT, MR, CT-PET and bone scans, of the abdominopelvic cavity, to identify current limitations and inform future research efforts.

For peer review only

METHODS

Protocol and registration

This systematic review was registered with the International Prospective Register of Systematic Reviews (PROSPERO: CRD42021237249). A study protocol has previously been published [13]. It is reported in accordance with the Preferred Reporting Items for Systematic Reviews and Meta-analysis of Diagnostic Test Accuracy Studies (PRISMA-DTA) [14].

Information sources

Electronic searches of OVID SP versions of Medline, EMBASE and the Cochrane Central Register of Controlled Trials (CENTRAL) databases were conducted to identify all potentially relevant studies. Date limitations between January 1st 2012 and July 31st 2021 were applied, to account for advancements in machine learning performance and the development of deep learning approaches since 2012, in line with existing reviews [15]. Reference lists of included articles were screened to identify further relevant studies.

Search strategy and study selection

A comprehensive search syntax was developed with adaptation from three existing search strategies [6,7,16] and guidance from an information specialist using text words and Medical Subject Headings (MeSH) related to three domains: “artificial intelligence”, “diagnostic imaging” and the “abdominopelvic cavity” (Appendix S1). Database search results were imported into reference management software (EndNote X9, Clarivate Analytics, USA) and duplicates removed.

For peer review only

Assessment of study eligibility was performed in two stages. First, titles and abstracts were screened for inclusion by two independent reviewers (GEF and CH). Any conflicts were resolved through discussion, referring to the wider study team if required. Final eligibility was assessed by a full-text review of potentially eligible studies by the same process. Management of the screening process was aided by Rayyan software (Rayyan Systems Inc., Cambridge, MA, USA) [17].

Eligibility criteria

Primary research studies were considered for eligibility using the PIRT (participants, index test(s), reference standard and target condition) framework [18]. Participants were adults with pathology within the abdominopelvic cavity diagnosed using the following radiological modalities: CT, MR, CT-PET or bone scans. Diagnostic endoscopy was excluded as existing reviews have explored the performance of AI models in this area [19,20]. The index test was studies considering AI models as an intervention with the aim to provide a diagnosis. The reference standard was 'standard practice' to allow for variation across the included studies. The target condition was abdominopelvic cavity pathology which has had, or may warrant, an invasive procedure [21] for therapeutic intent.

Excluded were secondary research studies (e.g., systematic reviews), case reports and case series, absence of full text (e.g., conference abstracts), animal studies and non-English articles.

Data extraction and management

For peer review only

Data extraction from the included articles was independently performed by two reviewers (GEF and CH). Data management software (REDCap version 9.5.23, Vanderbilt University, USA) [22] and a pre-designed standardized form were used. Data were extracted under the following three subheadings:

(1) Study characteristics

Extracted data included the name of the first author, their affiliated country, composition of the study team (e.g., software engineers, radiologists, and surgeons who routinely operate on surgical pathology within the abdominopelvic cavity (e.g., gastrointestinal surgeons, urologists and gynaecologists)), year of publication, study aim and design (i.e., ‘prospective’ or ‘retrospective’), surgical subspeciality and pathology studied (i.e., benign, malignant tumours, multiple or other). Information on the reporting of ethics and/or regulatory approval (e.g., Medicines and Healthcare products Regulatory Agency), patient and publication involvement and authors’ mention of using a reporting guideline (e.g., Standards for Reporting of Diagnostic Accuracy Studies) was recorded.

(2) Training data

Extracted data on the input features (data used to develop the AI model) included the modality of cross-sectional radiological imaging (CT, MR, CT-PET and bone scans), the AI model used, the reference standard and the reporting and size of the training and test sets. Information on whether the training data came from the studies collected data or readily available datasets was recorded.

(3) Outcomes

For peer review only

The performance of the AI models and human comparator (where applicable) were extracted. Diagnostic measures of accuracy included reported sensitivity, specificity, positive predictive values and the area under the receiver operating characteristic curve (AUC). The interpretation time (seconds) for studies comparing the performance of the AI model with a human comparator (where reported) were extracted.

Risk of bias and applicability

Risk of bias was assessed independently by two reviewers (GEF and RCM) using the QUADAS-2 tool [23]. A version of the QUADAS-2 tool for AI studies [24] was still in development and not available at the time of conducting the current review. The generic QUADAS-2 tool with the pre-existing modified signalling questions was used to assess four domains including patient selection, index test, reference standard and flow and timing [23]. An overall judgement of "at risk of bias" or "concerns regarding applicability" was assigned if more one or more domains were judged as "high" or "unclear" [23]. Judgements of applicability assessed whether the study matched the review question [23].

Data synthesis

A narrative synthesis was conducted according to the Synthesis Without Meta-analysis guidelines [25]. The synthesis was planned to focus on the primary outcome with studies grouped by the modality of radiological imaging, surgical subspeciality and pathology studied, as outlined in the protocol [13]. A broader approach was, however, adopted due to the small number of included studies and their heterogeneity. A meta-analysis was not performed due to the broad nature of the included studies.

For peer review only

Patient and public involvement

As part of the wider programme of work (Bristol Biomedical Research Centre, National Institute for Health Research (NIHR) Bristol BRC), patients and the public were consulted on their views of AI being used to guide doctors to make decisions about treatment. Overall, it was perceived positively, and they were supportive of its adoption in healthcare.

For peer review only

RESULTS

Database searching identified 628 records, with a further five studies identified through reference lists of included articles. After the removal of duplicates, 580 were screened and, 52 full-text articles were assessed for eligibility. Fifteen studies were finally included (Figure 1) [26–40].

Study characteristics

Characteristics of the included studies and details of the AI models are summarised (Tables 1 and 2). All were retrospective studies conducted in six different countries: Japan (n=4), USA (n=4), China (n=3), India (n=2), Turkey (n=1) and South Korea (n=1). Studies were all proof-of-concept (i.e., not applied in a clinical setting;), from four surgical specialities: urology (n=6), gastrointestinal surgery (n=6), endocrine surgery (n=1) and gynaecology (n=1). One study was not specific to a single speciality and involved whole-body CT-PET across four anatomical regions (head-and-neck, chest, abdomen, and pelvis)[29]. Most studies focused on malignant tumours (n=11) using CT as the imaging modality (n=8). Most studies (n=14) included an ethical approval statement. No studies, however, mentioned patient and public involvement or the use of a reporting guideline.

For peer review only

Table 1. Characteristics of the included studies

Characteristics	Number of studies n=15
**Country of origin**	
Japan	4
USA	4
China	3
India	2
Turkey	1
South Korea	1
**Year of publication**	
2020	5
2019	5
2018	2
2017	1
2016	1
2013	1
**Surgical subspecialty**	
Urology	6
Gastrointestinal	6
Endocrine	1
Gynaecology	1
Not reported	1
**Pathology studied**	
Malignant tumours	11
Multiple pathologies	2
Other	2
**Modality of radiological imaging**	
CT	8
CT-PET	2
MR	2
PET	1
MR and CT-PET	1
Bone scans	1

Abbreviations: CT (computer tomography), PET (positron emission tomography) and MR (magnetic resonance).

For peer review only

Table 2. Study details and AI models

First author (Reference)	Country of origin	Surgical speciality	Pathology studied	Modality of radiological imaging	AI models	Reference standard	Size of training set (patients)	Size of test set (patients)
**Acar [25]**	Turkey	Urology	Malignancy	CT-PET	KNN	2 Nuclear Medicine Physicians	"90% [total 75 patients]"	"10% [total 75 patients]"
**Coy [26]**	USA	Urology	Malignancy	CT	CNN	Pathologist (number not specified)	90% [total 179 patients]	10% [total 179 patients]
**Han [32]**	South Korea	Urology	Malignancy	CT	CNN	Pathologist (number not specified)	135	34
**Koizumi [34]**	Japan	Urology	Malignancy	Bone scans	ANN	2 Radiology Nuclear Physicians	Not reported	226
**Lee [35]**	USA	Urology	Malignancy	PET	CNN	1 Imaging Physicians	Not reported	Not reported
**Oberai [39]**	USA	Urology	Malignancy	CT	CNN	1 Genitourinary Pathologists	120	23
**Lu [36]**	China	Gastrointestinal	Malignancy	MR	CNN	Radiologists (number not specified)	Not reported	Not reported (+ 414 validation test set)
**Nayak [38]**	India	Gastrointestinal	Multiple (cirrhosis, hepatocellular carcinoma)	CT	SVM	1 Radiologist	40	Not reported
**Sethi [28]**	India	Gastrointestinal	Multiple (normal, cystic, calculus or tumour tissues)	CT	ANN, SVM	Unclear	Not reported	Not reported
**Yasaka [29]**	Japan	Gastrointestinal	Malignancy	CT	CNN	Radiologists (number not specified)	460	100

For peer review only

**Yuan [30]**	China	Gastrointestinal	Malignancy	CT	SVM, CNN	1 Pathologist	130	40
**Zhao [31]**	China	Gastrointestinal	Malignancy	MR	CNN	3 Radiologists	293	81
**Saiprasad [27]**	USA	Endocrine Surgery	Other (normal vs abnormal)	CT	RFC	2 Radiologists	5	10
**Nakagawa [37]**	Japan	Gynaecology	Malignancy	MR and CT-PET	Logistic regression (univariate and multivariate)	2 Radiologists and pathologists (number not specified)	66	Unclear
**Kawauchi, [33]**	Japan	Uncharacterized	Other (benign/malignant /equivocal)	CT-PET	CNN	1 Nuclear Medicine Physician	2440	1045

Abbreviations: Artificial neural network (ANN), Computerized tomography (CT), convolutional neural network (CNN), k-nearest neighbours algorithm (KNN), magnetic resonance (MR), positron emission tomography (PET), random forest classification (RFC) and support vector machine (SVM).

For peer review only

The composition and expertise within each study team varied. Three studies (n=3) comprised teams including software engineers, radiologists and surgeons. A small number of studies were comprised of only radiologists (n=2) or software engineers alone (n=1). Four study teams were comprised software engineers and radiologists together. Of the remaining five studies, three had no apparent radiological team and two were comprised of radiologists with either a team of software engineers, surgeons or physicians.

Training data

AI training and test sets within the included studies comprised a median of 130 (range: 5 – 2440) and 37 (range: 10 – 1045) patients, respectively (Table 2). This information, however, was not always available (n=6 studies). All training data came from the studies collected data and not from pre-existing readily available datasets. There was variability in the reference standard, including the number of clinicians (range: 1–3) for either a radiological (n=9) or histological (n=4) diagnosis or both (n=1). Only one study had an unclear reference standard (Table 2).

Outcomes

The intent of the AI applications in the included studies varied, with the majority focusing on diagnosing advanced or recurrent cancer (n=6 studies) and four studies classifying the pathology (i.e., normal or abnormal, or benign or malignant) (online supplementary Table 1). Diagnostic performance of the AI models ranged between 70.0 and 95.0% sensitivity, and 52.9% and 98.0% specificity (online supplementary Table 1). The reporting of the diagnostic measures of accuracy were unstandardised,

For peer review only

for example, there was different output measures across the studies and three studies did not report all of their outcome measures (online supplementary Table 1).

Following model development with a training and tuning set, four studies had an external validation test set and compared the performance of the AI model with a human comparator (a radiologist) (online supplementary Table 1) [32,33,39,40]. The diagnostic performance of the radiologists ranged between 57.4% and 62.8% sensitivity and 0.89 and 0.97 AUC (online supplementary Table 1). In these studies, there was variability in both the number of patients (range 50–414; from one to six different centres) and number of radiologists (range 2–4) and their years of experience. These studies reported the diagnostic performance of the AI model as either superior (n=2; in rectal and advanced gastrointestinal cancer) [39,40] or comparable (n=2; in metastatic gastrointestinal pathology and a gynaecology study distinguishing between malignant tumours and benign pathology) to radiologists [32,33]. Two of these studies included a comparison of the interpretation time of the AI model with the radiologists [32,40]. AI models outperformed the radiologists in both studies (1–2 seconds vs 200 seconds per case, $p<0.05$ [40] and 20s vs 600s for an average of 100 MR images [32]).

For peer review only

Risk of bias and applicability

With the exception of one study [40], all the included studies had an overall judgement of “at risk of bias” and “concerns regarding applicability” (Table 3). This was predominantly due to comparisons based on either a single clinician’s assessment (n=10) or an unclear number of clinicians for the assessment (n=5), from either a small (e.g., 10 patients in one study) or unclear size of test set and most models were

developed only from internal validations (n=11) (Table 2 and online supplementary Table 1).

For peer review only

Table 3. Study quality assessment (QUADAS-2 tool)

First author (reference)	RISK OF BIAS				APPLICABILITY CONCERNS		
	PATIENT SELECTION	INDEX TEST	REFERENCE STANDARD	FLOW AND TIMING	PATIENT SELECTION	INDEX TEST	REFERENCE STANDARD
Acar [25]	Low	Low	High	Low	Low	Low	High
Coy [26]	Low	Low	High	Low	Low	Low	High
Han [32]	Unclear	Low	High	Low	Low	Low	High
Kawauchi [33]	Low	Low	High	Low	Low	Low	High
Lu [36]	Unclear	Low	High	Low	Low	Low	High
Nakagawa [37]	Low	Low	High	Low	Low	Low	High
Koizumi [34]	Low	Low	High	Low	Low	Low	High
Lee [35]	Low	Low	High	Low	Low	Low	High
Nayak [38]	Unclear	Low	Unclear	Low	Low	Low	High
Oberai [39]	Low	Low	High	Low	Low	Low	High
Yasaka [29]	Unclear	Low	High	Low	Low	Low	High
Saiprasad [27]	Unclear	Low	High	Low	Low	Low	High
Sethi [28]	High	Low	Unclear	Low	Low	Low	Unclear
Yuan [30]	Low	Low	High	Low	Low	Low	High
Zhao [31]	Low	Low	Low	Low	Low	Low	Low

For peer review only

DISCUSSION

The major finding of this review was the heterogeneity in the AI applications across the included studies regardless of the surgical speciality or pathology. Early phase studies of AI innovation, particularly focusing on advanced or recurrent malignancy, were identified with promising diagnostic accuracies to support clinical decision making. Future AI research could benefit from targeting areas where radiological expertise is in high demand or the data is complex to interpret; for example, adrenal incidentalomas [41] and images from virtual colonoscopy [2]. Attention should also be directed to the governance of AI, particularly on where the responsibility lies if the AI model misses a lesion.

In this review, several reporting issues were identified, including for the reference standard and training data. Poor adherence to reporting guidelines is a common finding in the existing literature for diagnostic accuracy studies assessing AI interventions [4,11]. The Standards for Reporting of Diagnostic Accuracy-AI (STARD-AI) Steering Group are developing an AI-specific extension to the STARD statement, which aims to improve reporting of AI diagnostic accuracy studies [42]. This steering group highlighted three pitfalls, which are also reflected in this review: (1) unclear methodological interpretation (e.g. methods of validation and comparison to human performance), (2) unstandardised nomenclature (e.g. varying definition of the term 'validation'), and (3) heterogeneity of the outcome measures (e.g. sensitivity, specificity, predictive values and AUC) [43]. Endeavours to address this include the development of specific reporting guidelines for authors of AI studies, including protocols (SPIRIT-AI) [44], reports (CONSORT-AI) [45] and proposals (MINIMAR)

For peer review only

[46]. These efforts should improve both the reporting quality and make it easier to interpret and compare AI studies.

A minority of the included studies compared the diagnostic performance of the AI model with a clinician's diagnosis. These studies reported a faster and superior or equivalent diagnostic performance with the AI model. A recent review found only 51 studies worldwide reporting the implementation and evaluation of AI applications in clinical practice [47]. While many AI studies are currently retrospective and proof-of-concept [10,11,47], which may be appropriate for early phase surgical research, future efforts should evaluate the role of AI in a clinical setting. This should adopt a multidisciplinary team of all relevant stakeholders (e.g., software engineers, radiologists, and surgeons) to ensure the diverse and relevant skills sets can work together to produce both high-quality and clinically relevant AI research.

This review included a robust methodology, comprehensive search strategy and a multidisciplinary team. Some limitations, however, are acknowledged. Despite having a broad search strategy, relevant studies may have been missed by excluding articles that were not published in the English language. It did not encompass all diagnostic applications of AI in this region, such as diagnostic imaging for prognostic, surveillance, and management decisions meaning findings are not generalisable to these wider contexts. However, recommendations for prioritising future endeavours on clinical need, adhering to reporting guidelines and standardised and transparent reporting can be considered appropriate for all studies assessing AI interventions in healthcare.

For peer review only

This review identified a diverse application of AI innovation in this field. Most studies were proof-of-concept and more ‘comparator’ studies in the clinical setting are needed. Future AI research could build on existing studies with translation to clinical practice, adopting a multidisciplinary approach, including patient and public involvement, which was lacking in the studies of this review. This could target areas of clinical need. Adherence to existing and developing guidelines for reporting AI studies, such as SPIRIT-AI [44], CONSORT-AI [45], STARD-AI [43] and DECIDE-AI [48], are warranted.

For peer review only

ETHICS STATEMENT

Ethical approval was not required as no primary data were collected.

CONTRIBUTORS

GEF and NSB conceived the idea for this systematic review. All authors (GEF, NSB, CH, MPC, NJS and RCM) contributed to the design of the study. GEF and CH performed screening and data extraction. GEF and RCM conducted the QUADAS-2 assessments. All authors (GEF, NSB, CH, MPC, NJS and RCM) contributed towards the data analysis and interpretation of the data. GEF drafted the manuscript (guarantor of review) and all authors (GEF, NSB, CH, MPC, NJS and RCM) were involved in critiquing the manuscript. All authors approved the final manuscript before submission.

COMPETING INTERESTS

The authors have no conflicts of interest to declare.

FUNDING

NSB is funded by an MRC Clinical Scientist Award (grant number: MR/S001751/1). This study was supported by the National Institute for Health and Care Research Bristol Biomedical Research Centre. The views expressed are those of the author(s) and not necessarily those of the NIHR or the Department of Health and Social Care.

ACKNOWLEDGEMENTS

The authors would like to thank Ms Catherine Borwick, Information Specialist, University of Bristol, for her input and expertise to develop the search strategy. The authors are grateful to Dr Christin Hoffmann (University of Bristol) for leading patient

For peer review only

and public feedback sessions as part of the wider programme of this research and Dr Xiaoxuan Liu (University of Birmingham and University Hospitals Birmingham NHS Foundation Trust) for providing expert comments on the manuscript.

DATA AVAILABILITY STATEMENT

All data relevant to the study are included in the article or uploaded as supplementary information.

For peer review only

References

1 Oren O, Gersh BJ, Bhatt DL. Artificial intelligence in medical imaging: switching from radiographic pathological data to clinically meaningful endpoints. *Lancet Digit Health* 2020;**2**:e486–8. doi:10.1016/S2589-7500(20)30160-6
2 Hosny A, Parmar C, Quackenbush J, *et al.* Artificial intelligence in radiology. *Nat Rev Cancer* 2018;**18**:500–10. doi:10.1038/s41568-018-0016-5.Artificial
3 He J, Baxter SL, Xu J, *et al.* The practical implementation of artificial intelligence technologies in medicine. *Nat Med* 2019;**25**:30–6. doi:10.1038/s41591-018-0307-0
4 Aggarwal R, Sounderajah V, Martin G, *et al.* Diagnostic accuracy of deep learning in medical imaging: a systematic review and meta-analysis. *NPJ Digit Med* 2021;**4**. doi:10.1038/s41746-021-00438-z
5 Jin P, Ji X, Kang W, *et al.* Artificial intelligence in gastric cancer: a systematic review. *J Cancer Res Clin Oncol* 2020;**146**:2339–50. doi:10.1007/s00432-020-03304-9
6 Senders JT, Arnaout O, Karhade A v., *et al.* Natural and artificial intelligence in neurosurgery: A systematic review. *Clin Neurosurg* 2018;**83**:181–92. doi:10.1093/neuros/nyx384
7 Raffort J, Adam C, Carrier M, *et al.* Artificial intelligence in abdominal aortic aneurysm. *J Vasc Surg* 2020;**72**:321-333.e1. doi:10.1016/j.jvs.2019.12.026
8 Esteva A, Kuprel B, Novoa RA, *et al.* Dermatologist-level classification of skin cancer with deep neural networks. *Nature* 2017;**542**:115–8. doi:10.1038/nature21056
9 Lin H, Li R, Liu Z, *et al.* Diagnostic Efficacy and Therapeutic Decision-making Capacity of an Artificial Intelligence Platform for Childhood Cataracts in Eye Clinics: A Multicentre Randomized Controlled Trial. *EClinicalMedicine* 2019;**9**:52–9. doi:10.1016/j.eclinm.2019.03.001
10 Ben-Israel D, Jacobs WB, Casha S, *et al.* The impact of machine learning on patient care: A systematic review. *Artif Intell Med* 2020;**103**:101785. doi:10.1016/j.artmed.2019.101785
11 Nagendran M, Chen Y, Lovejoy CA, *et al.* Artificial intelligence versus clinicians: Systematic review of design, reporting standards, and claims of deep learning studies in medical imaging. *The BMJ* 2020;**368**:1–12. doi:10.1136/bmj.m689
12 Yusuf M, Atal I, Li J, *et al.* Reporting quality of studies using machine learning models for medical diagnosis: a systematic review. *BMJ Open* 2020;**10**:e034568. doi:10.1136/bmjopen-2019-034568
13 Fowler GE, Macefield RC, Hardacre C, *et al.* Artificial intelligence as a diagnostic aid in cross-sectional radiological imaging of the abdominopelvic cavity: a protocol for a systematic review. *BMJ Open* 2021;**11**:e054411. doi:10.1136/bmjopen-2021-054411
14 McInnes MDF, Moher D, Thombs BD, *et al.* Preferred Reporting Items for a Systematic Review and Meta-analysis of Diagnostic Test Accuracy Studies The PRISMA-DTA Statement. *JAMA - Journal of the American Medical Association* 2018;**319**:388–96. doi:10.1001/jama.2017.19163
15 Liu X, Faes L, Kale AU, *et al.* A comparison of deep learning performance against health-care professionals in detecting diseases from medical imaging: a systematic review and meta-analysis. *Lancet Digit Health* 2019;**1**:e271–97. doi:10.1016/S2589-7500(19)30123-2
16 Yang Y, Jin G, Pang Y, *et al.* The diagnostic accuracy of artificial intelligence in thoracic diseases. *Medicine* 2020;**99**:e19114. doi:10.1097/md.0000000000019114
17 Ouzzani M, Hammady H, Fedorowicz Z, *et al.* Rayyan-a web and mobile app for systematic reviews. *Syst Rev* 2016;**5**:1–10. doi:10.1186/s13643-016-0384-4

18 JJ, Deeks, S, Wisniewski DC, Diagnostic C, Accuracy T, *et al.* Guide to the contents of a Cochrane Diagnostic Test Accuracy Protocol. *Cochrane Handbook for Systematic Reviews of Diagnostic Test Accuracy Version 100 The Cochrane Collaboration* 2013;:1–15.

19 Hassan C, Spadaccini M, Iannone A, *et al.* Performance of artificial intelligence for colonoscopy regarding adenoma and polyp detection: a meta-analysis. *Gastrointest Endosc* Published Online First: 2020. doi:10.1016/j.gie.2020.06.059

20 Lui TKL, Tsui VWM, Leung WK. Accuracy of artificial intelligence–assisted detection of upper GI lesions: a systematic review and meta-analysis. *Gastrointest Endosc* 2020;**92**:821-830.e9. doi:10.1016/j.gie.2020.06.034

21 Cousins S, Blencowe NS, Blazeby JM. What is an invasive procedure? A definition to inform study design, evidence synthesis and research tracking. *BMJ Open* 2019;**9**:2018–20. doi:10.1136/bmjopen-2018-028576

22 Harris PA, Taylor R, Minor BL, *et al.* The REDCap consortium: Building an international community of software platform partners. *J Biomed Inform* 2019;**95**:103208. doi:10.1016/j.jbi.2019.103208

23 Whiting PF, Rutjes AW, Westwood ME, Mallett S, Deeks JJ, Reitsma JB, Leeflang MM, Sterne JA BP. QUADAS-2 Group. QUADAS-2: a revised tool for the quality assessment of diagnostic accuracy studies. *Ann Intern Med* 2011;**8**:29–36. doi:0.7326/0003-4819-155-8-201110180-00009. PMID: 22007046.

24 Viknesh Sounderajah, Hutan Ashrafian, Sherri Rose, Nigam H. Shah, Marzyeh Ghassemi, Robert Golub, Charles E. Kahn Jr, Andre Esteva, Alan Karthikesalingam, Bilal Mateen, Dale Webster, Dan Milea, Daniel Ting, Darren Treanor, Dominic Cushnan, Dominic King, D AD. A quality assessment tool for artificial intelligence-centered diagnostic test accuracy studies: QUADAS-AI. *Nat Med* 2021;**27**:1663–5. doi:10.1038/s41591-021-01494-4

25 Campbell M, McKenzie JE, Sowden A, *et al.* Synthesis without meta-analysis (SWiM) in systematic reviews: Reporting guideline. *The BMJ* 2020;**368**:1–6. doi:10.1136/bmj.l6890

26 Acar E, Leblebici A, Ellidokuz BE, *et al.* Full paper: Machine learning for differentiating metastatic and completely responded sclerotic bone lesion in prostate cancer: A retrospective radiomics study. *British Journal of Radiology* 2019;**92**. doi:http://dx.doi.org/10.1259/bjr.20190286

27 Coy H, Hsieh K, Wu W, *et al.* Deep learning and radiomics: the utility of Google TensorFlow™ Inception in classifying clear cell renal cell carcinoma and oncocytoma on multiphasic CT. *Abdominal Radiology* 2019;**44**:2009–20. doi:10.1007/s00261-019-01929-0

28 Han S, Hwang S Il, Lee HJ. The Classification of Renal Cancer in 3-Phase CT Images Using a Deep Learning Method. *J Digit Imaging* 2019;**32**:638–43. doi:10.1007/s10278-019-00230-2

29 Kawauchi K, Furuya S, Hirata K, *et al.* A convolutional neural network-based system to classify patients using FDG PET/CT examinations. *BMC Cancer* 2020;**20**:227. doi:https://dx.doi.org/10.1186/s12885-020-6694-x

30 Koizumi M, Motegi K, Koyama M, *et al.* Diagnostic performance of a computer-assisted diagnosis system for bone scintigraphy of newly developed skeletal metastasis in prostate cancer patients: search for low-sensitivity subgroups. *Ann Nucl Med* 2017;**31**:521–8. doi:http://dx.doi.org/10.1007/s12149-017-1175-2

31 Lee JJ, Yang H, Franc BL, *et al.* Deep learning detection of prostate cancer recurrence with 18F-FACBC (fluciclovine, Axumin®) positron emission tomography. *Eur J Nucl Med Mol Imaging* 2020;**47**:2992–7. doi:10.1007/s00259-020-04912-w

For peer review only

32 Lu Y, Yu Q, Gao Y, *et al.* Identification of metastatic lymph nodes in MR imaging with faster region-based convolutional neural networks. *Cancer Res* 2018;**78**:5135–43. doi:10.1158/0008-5472.CAN-18-0494

33 Nakagawa M, Nakaura T, Namimoto T, *et al.* A multiparametric MRI-based machine learning to distinguish between uterine sarcoma and benign leiomyoma: comparison with 18F-FDG PET/CT. *Clin Radiol* 2019;**74**:167.e1-167.e7. doi:10.1016/j.crad.2018.10.010

34 Nayak A, Baidya Kayal E, Arya M, *et al.* Computer-aided diagnosis of cirrhosis and hepatocellular carcinoma using multi-phase abdomen CT. *Int J Comput Assist Radiol Surg* 2019;**14**:1341–52. doi:http://dx.doi.org/10.1007/s11548-019-01991-5

35 Oberai A, Varghese B, Cen S, *et al.* Deep learning based classification of solid lipid-poor contrast enhancing renal masses using contrast enhanced CT. *Br J Radiol* 2020;**93**:20200002. doi:http://dx.doi.org/10.1259/bjr.20200002

36 Saiprasad G, Chang CI, Safdar N, *et al.* Adrenal gland abnormality detection using random forest classification. *J Digit Imaging* 2013;**26**:891–7. doi:http://dx.doi.org/10.1007/s10278-012-9554-7

37 Sethi G, Saini BS. Computer aided diagnosis system for abdomen diseases in computed tomography images. *Biocybern Biomed Eng* 2016;**36**:42–55. doi:10.1016/j.bbe.2015.10.008

38 Yasaka K, Akai H, Abe O, *et al.* Deep learning with CNN showed high diagnostic performance in differentiation of liver masses at dynamic CT. *Radiology* 2018;**286**:887–96.

39 Yuan Z, Xu T, Cai J, *et al.* Development and Validation of an Image-based Deep Learning Algorithm for Detection of Synchronous Peritoneal Carcinomatosis in Colorectal Cancer. *Ann Surg* 2020;**16**. doi:http://dx.doi.org/10.1097/SLA.0000000000004229

40 Zhao X, Xie P, Wang M, *et al.* Deep learning-based fully automated detection and segmentation of lymph nodes on multiparametric-mri for rectal cancer: A multicentre study. *EBioMedicine* 2020;**56**:102780. doi:https://dx.doi.org/10.1016/j.ebiom.2020.102780

41 Garrett RW, Nepute JC, El Hayek M, *et al.* Adrenal Incidentalomas: Clinical controversies and modified recommendations. *American Journal of Roentgenology* 2016;**206**:1170–8. doi:10.2214/AJR.15.15475

42 Sounderajah V, Ashrafian H, Karthikesalingham A, Marker SR, Normanhani P, Collins GS, Bossuyt PM DD. Developing Specific Reporting Standards in Artificial Intelligence Centred Research Viknesh. *Ann Surg* 2021;:6. doi:10.1097/SLA.0000000000005294

43 Sounderajah V, Ashrafian H, Aggarwal R, *et al.* Developing specific reporting guidelines for diagnostic accuracy studies assessing AI interventions: The STARD-AI Steering Group. *Nat Med* 2020;**26**:807–8. doi:10.1038/s41591-020-0941-1

44 Cruz Rivera S, Liu X, Chan AW, *et al.* Guidelines for clinical trial protocols for interventions involving artificial intelligence: the SPIRIT-AI extension. *Nat Med* 2020;**26**:1351–63. doi:10.1038/s41591-020-1037-7

45 Liu X, Cruz Rivera S, Moher D, *et al.* Reporting guidelines for clinical trial reports for interventions involving artificial intelligence: the CONSORT-AI extension. *Nat Med* 2020;**26**:1364–74. doi:10.1038/s41591-020-1034-x

46 Hernandez-Boussard T, Bozkurt S, Ioannidis JPA, *et al.* MINIMAR (MINimum Information for Medical AI Reporting): Developing reporting standards for artificial intelligence in health care. *Journal of the American Medical Informatics Association* 2020;**00**:1–5. doi:10.1093/jamia/ocaa088

For peer review only

47 Yin J, Ngiam KY TH. Role of Artificial Intelligence Applications in Real-Life Clinical Practice: Systematic Review. *J Med Internet Res* 2021;**23**:e25759. doi:10.2196/25759

48 Vasey B, Clifton DA, Collins GS, *et al.* DECIDE-AI: new reporting guidelines to bridge the development-to-implementation gap in clinical artificial intelligence. Nat Med. 2021;**27**:186–7. doi:10.1038/s41591-021-01229-5

FIGURE LEGEND/CAPTIONS

Figure 1. PRISMA flow diagram

For peer review only

Figure 1 PRISMA flow diagram

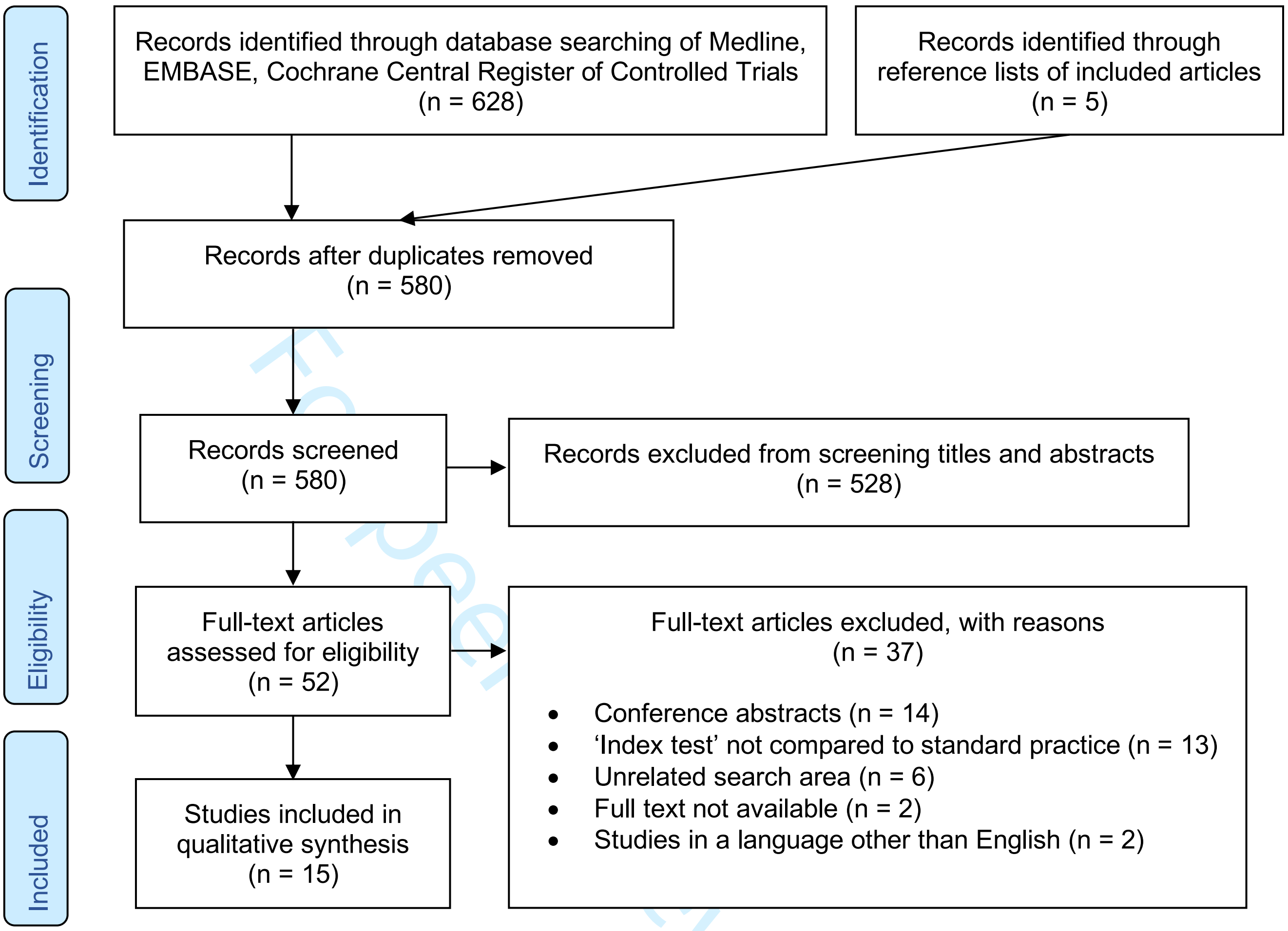

For peer review only

Online supplementary Table 1 Performance of AI models

First author (Reference)	Study aim	Best AI model	Output measures	AI performance	Human comparator	Human performance	External validation
**Acar [25]**	Distinguish the metastatic and completely responded areas regarding sclerotic bone lesions imaged via 68Ga-PSMA PET/CT in patients with known bone metastasis.	KNN	Sensitivity<br>Specificity<br>Predictive values (PPV, NPV)<br>AUC<br>Overall accuracy	73.5%<br>73.7%<br>86.9%, 53.8%<br>76.0%<br>73.5%	NA	NA	No
**Coy [26]**	Investigate the diagnostic value and feasibility of a deep learning-based renal lesion classifier in differentiating ccRCC from ONC on routine four-phase MDCT in patients with pathologically confirmed renal masses.	CNN	Sensitivity<br>Specificity<br>Predictive values (PPV, NPV)<br>Overall accuracy	88.3%<br>52.9%<br>79.6%, 59.5%<br>75.4%	NA	NA	No
**Han [32]**	Develop reproducible and generalizable models for discriminating three major subtypes of RCCs using CT image analysis along with a ML algorithm.	CNN	Sensitivity<br>Specificity<br>AUC<br>Overall accuracy	0.98<br>0.93<br>0.9<br>0.85	NA	NA	No
**Koizumi [34]**	Investigate the diagnostic performance of computer-assisted diagnostic system for bone scintigraphy in prostate cancer patients with and without skeletal metastasis.	ANN	Sensitivity<br>Specificity<br>AUC	82.0%<br>83.0%<br>0.89	NA	NA	No
**Lee [35]**	Evaluate the performance of deep learning classifiers in discriminating normal and abnormal 18F-FACBC PET scans based on the presence of tumour recurrence and/or metastases in patients with	CNN	Sensitivity<br>Specificity<br>AUC	90.7%<br>95.1%<br>0.97	NA	NA	No

For peer review only - http://bmjopen.bmj.com/site/about/guidelines.xhtml

	prostate cancer and biochemical recurrence.						
**Oberai [39]**	Classify lipid poor enhancing renal masses into 'benign' and 'malignant' categories.	CNN	Sensitivity<br>Specificity<br>AUC<br>Overall accuracy	70.0%<br>81.0%<br>0.82<br>78.0%	NA	NA	No
**Lu [36]**	To develop an assistive tool to aid radiologists to more effectively and accurately determine lymph node metastases.	CNN	Sensitivity<br>Specificity<br>AUC<br>Overall accuracy	Not reported<br>Not reported<br>0.91<br>99.9%	Radiologists (number not specified)	Values not explicitly reported.	Yes
**Nayak [38]**	Study proposes a computer-aided diagnosis system for detecting cirrhosis and HCC.	SVM	Prediction accuracy	80.8 - 86.9%	NA	NA	No
**Sethi [28]**	Paper proposed automated CAD system for classification of abdomen diseases like tumour, cyst, stone and normal tissues from CT images of abdomen.	ANN	Sensitivity<br>Specificity<br>Predictive values (PPV, NPV)<br>AUC<br>Overall accuracy	95.0%<br>98.0%<br>No raw data<br>No raw data<br>95.1%	NA	NA	No
**Yasaka [29]**	Investigate diagnostic performance by using a deep learning method with a CNN for the differentiation of liver masses at dynamic contrast agent-enhanced CT.	CNN	Sensitivity<br>Specificity<br>AUC<br>Overall accuracy	0.33 - 1.00<br>Not reported<br>0.92<br>0.84	NA	NA	No
**Yuan [30]**	Develop a CNN-based ResNet3d algorithm and SVM classified to detect synchronous peritoneal carcinomatosis in colorectal cancer.	CNN + SVM combination	Sensitivity<br>Specificity<br>Predictive values (PPV, NPV)<br>AUC<br>Overall accuracy	93.8%<br>94.4%<br>93.8%, 94.4%<br>0.92<br>94.1%	2 Radiologists (senior and junior)	57.4%, 58.1%<br>100%, 98%<br>No raw data<br>0.79, 0.78<br>No raw data	Yes
**Zhao [31]**	Develop and validate a deep-learning-based, fully automated lymph node detection and segmentation model based on mpMRI.	CNN	Sensitivity<br><br>Predictive values (PPV, NPV)	80.0%<br><br>73.5%, NA	4 Radiologists	38.2% (Int), 62.6% (Ext)<br>43.8% (Int), 35.9% (Ext)	Yes
**Saiprasad [27]**	Identify adrenal glands from a volumetric CT acquisition and	RFC	Sensitivity<br>Specificity	80.0%<br>90.0%	NA	NA	No

	determine whether they are normal or abnormal.						
**Nakagawa [37]**	Compare the performance of ML using mp-MRI and PET to distinguish between uterine sarcoma and leiomyoma.	Logistic regression (multivariate)	AUC	0.92	2 Radiologists (11 and 20 years of experience)	0.97 and 0.89	Yes
**Kawauchi [33]**	Develop a CNN-based system that can classify whole-body FDG PET as 1) benign, 2) malignant or 3) equivocal.	CNN	Prediction	91.0% - 94.9% (abdomen) 95.3% - 99.7% (pelvic region)	NA	NA	No

Abbreviations: Artificial neural network (ANN), area under the curve (AUC), benign oncocytoma (ONC), clear cell renal cell carcinoma (ccRCC), CNN (convolutional neural network), computer tomography (CT), Ext (external data set), hepatocellular carcinoma (HCC), Int (internal data set), machine learning (ML), k-nearest neighbours algorithm (KNN), multiparametric magnetic resonance imaging (mpMRI), negative predictive value (NPV), positive predictive value (PPV), positron emission tomography (PET), random forest classification (RFC) and support vector machine (SVM).
.

For peer review only

APPENDIX S1

Artificial intelligence as a diagnostic aid in cross-sectional radiological imaging of surgical pathology in the abdominopelvic cavity: A systematic review

Medline Search

1. artificial intelligence/ or machine learning/ or deep learning/ or supervised machine learning/ or support vector machine/ or unsupervised machine learning/

2. computer heuristics/ or expert systems/ or fuzzy logic/ or knowledge bases/ or biological ontologies/ or gene ontology/ or natural language processing/ or neural networks, computer/ or robotics/

3. Machine learning.ti,ab,kf.

4. Artificial Intelligence.ti,ab,kf.

5. Naive Bayes.ti,ab,kf.

6. bayesian learning.ti,ab,kf.

7. Neural network*.ti,ab,kf.

8. Natural language processing.ti,ab,kf.

9. support vector*.ti,ab,kf.

10. random forest*.ti,ab,kf.

11. boosting.ti,ab,kf.

12. deep learning.ti,ab,kf.

13. machine intelligence.ti,ab,kf.

14. computational intelligence.ti,ab,kf.

15. computer reasoning.ti,ab,kf.

16. 1 or 2 or 3 or 4 or 5 or 6 or 7 or 8 or 9 or 10 or 11 or 12 or 13 or 14 or 15

For peer review only

17. tomography/ or colonography, computed tomographic/ or computed tomography angiography/ or positron emission tomography computed tomography/

18. magnetic resonance imaging/ or cholangiopancreatography, magnetic resonance/ or diffusion magnetic resonance imaging/ or diffusion tensor imaging/ or echo-planar imaging/ or fluorine-19 magnetic resonance imaging/ or magnetic resonance angiography/ or magnetic resonance imaging, cine/ or multiparametric magnetic resonance imaging/

19. (MRI or Magnetic Resonance Imaging).ti,ab,kf.

20. 17 or 18 or 19

21. pelvis/ or lesser pelvis/ or pelvic floor/

22. (radiography adj3 abdominal).ti,ab,kf.

23. (Abdomen or abdominal cavity or peritoneum or douglas' pouch or mesentery or mesocolon or omentum or peritoneal cavity or peritoneal stomata or retroperitoneal space).ti,ab,kf.

24. (Pelvis or pelvic floor).ti,ab,kf.

25. 21 or 22 or 23 or 24

26. exp Animals/ not Humans/

27. (animal model* or rat or rats or mouse or mice or rodent* or sheep or lambs or murine or pigs or piglets or swine or porcine or rabbit or rabbits or cat or cats or feline or dog or dogs or canine or cattle or bovine or marmoset* or monkey or monkeys or trout or zebra fish*).ti.

28. (Comment or editorial or letter or case reports).pt.

29. (endoscope or gastroscope or colonoscope or capsule endoscopy or endoscopy).ti,ab,kf.

30. 26 or 27 or 28 or 29

For peer review only

31. 16 and 20 and 25

32. 31 not 30

33. (2012* or 2013* or 2014* or 2015* or 2016* or 2017* or 2018* or 2019* or 2020* or 202101*).yr,ed,dc,ep.

34. 32 and 33

For peer review only

Embase via Ovid

1. artificial intelligence/ or ambient intelligence/ or automated reasoning/ or computer heuristics/ or multicriteria decision analysis/

2. exp machine learning/

3. deep learning/

4. supervised machine learning/

5. exp support vector machine/

6. unsupervised machine learning/

7. hyperheuristics/ or metaheuristics/

8. expert systems/ or fuzzy logic/ or knowledge bases/ or natural language processing/ or neural networks, computer/ or robotics/

9. biological ontologies/ or gene ontology/

10. ((machine or bayesian or deep) adj1 learning).ti,ab,kw.

11. ((artificial or machine or computational) adj1 intelligence).ti,ab,kw.

12. Naive Bayes.ti,ab,kw.

13. Neural network*.ti,ab,kw.

14. Natural language processing.ti,ab,kw.

15. support vector*.ti,ab,kw.

16. random forest*.ti,ab,kw.

17. boosting.ti,ab,kw.

18. computer reasoning.ti,ab,kw.

19. 1 or 2 or 3 or 4 or 5 or 6 or 7 or 8 or 9 or 10 or 11 or 12 or 13 or 14 or 15 or 16 or 17 or 18

20. exp Tomography/

For peer review only

21. colonography, computed tomographic/ or computed tomography angiography/ or four-dimensional computed tomography/ or positron emission tomography computed tomography/ or single photon emission computed tomography computed tomography/ or tomography, spiral computed/

22. magnetic resonance imaging/ or cholangiopancreatography, magnetic resonance/ or diffusion magnetic resonance imaging/ or diffusion tensor imaging/ or echo-planar imaging/ or fluorine-19 magnetic resonance imaging/ or magnetic resonance angiography/ or magnetic resonance imaging, cine/ or multiparametric magnetic resonance imaging/

23. (MRI or Magnetic Resonance Imaging).ti,ab,kw.

24. 20 or 21 or 22 or 23

25. pelvis/ or lesser pelvis/ or pelvic floor/

26. (radiography adj3 abdominal).ti,ab,kw.

27. (Abdomen or abdominal cavity or peritoneum or douglas' pouch or mesentery or mesocolon or omentum or peritoneal cavity or peritoneal stomata or retroperitoneal space).ti,ab,kw.

28. (Pelvis or pelvic floor).ti,ab,kw.

29. 25 or 26 or 27 or 28

30. (Animal/ or Nonhuman/) not Human/

31. Animal Experiment/ not (Human Experiment/ or Human/)

32. (animal model* or rat or rats or mouse or mice or rodent* or sheep or lambs or murine or pigs or piglets or swine or porcine or rabbit or rabbits or cat or cats or feline or dog or dogs or canine or cattle or bovine or marmoset* or monkey or monkeys or trout or zebra fish*).ti.

33. (Comment or editorial or letter or case reports).pt.

For peer review only

34. (endoscope or gastroscope or colonoscope or capsule endoscopy or endoscopy).ti,ab,kw.

35. 30 or 31 or 32 or 33 or 34

36. 19 and 24 and 29

37. 36 not 35

38. (2012* or 2013* or 2014* or 2015* or 2016* or 2017* or 2018* or 2019* or 2020* or 202101*).yr,em.

39. 37 and 38

For peer review only

Cochrane Central Register of Controlled Trials

#1 MeSH descriptor: [Artificial Intelligence] this term only MeSH

#2 MeSH descriptor: [Machine Learning] this term only MeSH

#3 MeSH descriptor: [Deep Learning] this term only MeSH

#4 MeSH descriptor: [Supervised Machine Learning] this term only MeSH

#5 MeSH descriptor: [Unsupervised Machine Learning] this term only MeSH

#6 MeSH descriptor: [Support Vector Machine] this term only MeSH

#7 MeSH descriptor: [Computer Heuristics] this term only MeSH

#8 MeSH descriptor: [Expert Systems] this term only MeSH

#9 MeSH descriptor: [Fuzzy Logic] this term only MeSH

#10 MeSH descriptor: [Knowledge Bases] this term only MeSH

#11 MeSH descriptor: [Biological Ontologies] this term only MeSH

#12 MeSH descriptor: [Gene Ontology] this term only MeSH

#13 MeSH descriptor: [Natural Language Processing] this term only MeSH

#14 MeSH descriptor: [Neural Networks, Computer] this term only MeSH

#15 MeSH descriptor: [Robotics] this term only MeSH

#16 (machine learning): ti,ab,kw

#17 (artificial intelligence):ti,ab,kw

#18 (naive bayes):ti,ab,kw

#19 (bayesian learning):ti,ab,kw

#20 (neural network*):ti,ab,kw

#21 (natural language processing):ti,ab,kw

#22 (support vector*):ti,ab,kw

#23 (random forest*):ti,ab,kw

#24 (boosting):ti,ab,kw

For peer review only

#25 (deep learning):ti,ab,kw

#26 (machine intelligence):ti,ab,kw

#27 (computational intelligence):ti,ab,kw

#28 (computer reasoning):ti,ab,kw

#29 #1 or #2 or #3 or #4 or #5 or #6 or #7 or #8 or #9 or #10 or #11 or #12 or #13 or #14 or #15 or #16 or #17 or #18 or #19 or #20 or #21 or #22 or #23 or #24 or #25 or #26 or #27 or #28

#30 MeSH descriptor: [Tomography] this term only MeSH

#31 MeSH descriptor: [Colonography, Computed Tomographic] this term only MeSH

#32 MeSH descriptor: [Computed Tomography Angiography] this term only MeSH

#33 MeSH descriptor: [Positron-Emission Tomography] this term only MeSH

#34 MeSH descriptor: [Positron Emission Tomography Computed Tomography] this term only MeSH

#35 MeSH descriptor: [Magnetic Resonance Imaging] this term only MeSH

#36 MeSH descriptor: [Cholangiopancreatography, Magnetic Resonance] this term only MeSH

#37 MeSH descriptor: [Diffusion Magnetic Resonance Imaging] this term only MeSH

#38 MeSH descriptor: [Diffusion Tensor Imaging] this term only MeSH

#39 MeSH descriptor: [Echo-Planar Imaging] this term only MeSH

#40 MeSH descriptor: [Fluorine-19 Magnetic Resonance Imaging] this term only MeSH

#41 MeSH descriptor: [Magnetic Resonance Angiography] this term only MeSH

#42 MeSH descriptor: [Magnetic Resonance Imaging, Cine] this term only MeSH

#43 MeSH descriptor: [Multiparametric Magnetic Resonance Imaging] this term only MeSH

For peer review only

#44 (MRI):ti,ab,kw

#45 (magnetic resonance imaging):ti,ab,kw

#46 #30 or #31 or #32 or #33 or #34 or #35 or #36 or #37 or #38 or #39 or #40 or #41 or #42 or #43 or #44 or #45

#47 MeSH descriptor: [Pelvis] this term only MeSH

#48 MeSH descriptor: [Lesser Pelvis] this term only MeSH

#49 MeSH descriptor: [Pelvic Floor] this term only MeSH

#50 ((radiography NEAR/3 abdominal)):ti,ab,kw

#51 ((radiography NEAR/3 abdomen)):ti,ab,kw

#52 ((Abdomen or abdominal cavity or peritoneum or douglas' pouch or mesentery or mesocolon or omentum or peritoneal cavity or peritoneal stomata or retroperitoneal space)):ti,ab,kw

#53 (pelvis or pelvic floor):ti,ab,kw

#54 #47 or #48 or #49 or #50 or #51 or #52 or #53

#55 #29 and #46 and #54

For peer review only

PRISMA-DTA for Abstracts Checklist

Section/topic	#	PRISMA-DTA for Abstracts Checklist item	Reported on page #
**TITLE and PURPOSE**			
Title	1	Identify the report as a systematic review (+/- meta-analysis) of diagnostic test accuracy (DTA) studies.	1
Objectives	2	Indicate the research question, including components such as participants, index test, and target conditions.	3
**METHODS**			
Eligibility criteria	3	Include study characteristics used as criteria for eligibility.	3
Information sources	4	List the key databases searched and the search dates.	3
Risk of bias & applicability	5	Indicate the methods of assessing risk of bias and applicability.	3
Synthesis of results	A1	Indicate the methods for the data synthesis.	3
**RESULTS**			
Included studies	6	Indicate the number and type of included studies and the participants and relevant characteristics of the studies (including the reference standard).	3
Synthesis of results	7	Include the results for the analysis of diagnostic accuracy, preferably indicating the number of studies and participants. Describe test accuracy including variability; if meta-analysis was done, include summary results and confidence intervals.	3
**DISCUSSION**			
Strengths and limitations	9	Provide a brief summary of the strengths and limitations of the evidence	4
Interpretation	10	Provide a general interpretation of the results and the important implications.	4
**OTHER**			
Funding	11	Indicate the primary source of funding for the review.	24
Registration	12	Provide the registration number and the registry name	4

Adapted From: McInnes MDF, Moher D, Thombs BD, McGrath TA, Bossuyt PM, The PRISMA-DTA Group (2018). Preferred Reporting Items for a Systematic Review and Meta-analysis of Diagnostic Test Accuracy Studies: The PRISMA-DTA Statement. JAMA. 2018 Jan 23;319(4):388-396. doi: 10.1001/jama.2017.19163.

For more information, visit: www.prisma-statement.org.

For peer review only - http://bmjopen.bmj.com/site/about/guidelines.xhtml

PRISMA-DTA Checklist

Section/topic	#	PRISMA-DTA Checklist Item	Reported on page #
**TITLE / ABSTRACT**			
Title	1	Identify the report as a systematic review (+/- meta-analysis) of diagnostic test accuracy (DTA) studies.	1
Abstract	2	Abstract: See PRISMA-DTA for abstracts.	3-4
**INTRODUCTION**			
Rationale	3	Describe the rationale for the review in the context of what is already known.	6-7
Clinical role of index test	D1	State the scientific and clinical background, including the intended use and clinical role of the index test, and if applicable, the rationale for minimally acceptable test accuracy (or minimum difference in accuracy for comparative design).	6-7
Objectives	4	Provide an explicit statement of question(s) being addressed in terms of participants, index test(s), and target condition(s).	9
**METHODS**			
Protocol and registration	5	Indicate if a review protocol exists, if and where it can be accessed (e.g., Web address), and, if available, provide registration information including registration number.	8
Eligibility criteria	6	Specify study characteristics (participants, setting, index test(s), reference standard(s), target condition(s), and study design) and report characteristics (e.g., years considered, language, publication status) used as criteria for eligibility, giving rationale.	9
Information sources	7	Describe all information sources (e.g., databases with dates of coverage, contact with study authors to identify additional studies) in the search and date last searched.	8
Search	8	Present full search strategies for all electronic databases and other sources searched, including any limits used, such that they could be repeated.	8-9
Study selection	9	State the process for selecting studies (i.e., screening, eligibility, included in systematic review, and, if applicable, included in the meta-analysis).	8-9
Data collection process	10	Describe method of data extraction from reports (e.g., piloted forms, independently, in duplicate) and any processes for obtaining and confirming data from investigators.	9-11
Definitions for data extraction	11	Provide definitions used in data extraction and classifications of target condition(s), index test(s), reference standard(s) and other characteristics (e.g. study design, clinical setting).	9-11
Risk of bias and applicability	12	Describe methods used for assessing risk of bias in individual studies and concerns regarding the applicability to the review question.	11
Diagnostic accuracy measures	13	State the principal diagnostic accuracy measure(s) reported (e.g. sensitivity, specificity) and state the unit of assessment (e.g. per-patient, per-lesion).	10-11
Synthesis of results	14	Describe methods of handling data, combining results of studies and describing variability between studies. This could include, but is not limited to: a) handling of multiple definitions of target condition. b) handling of multiple thresholds of test positivity, c) handling multiple index test readers, d) handling of indeterminate test results, e) grouping and comparing tests, f) handling of different reference standards	11

For peer review only - http://bmjopen.bmj.com/site/about/guidelines.xhtml

PRISMA-DTA Checklist

Section/topic	#	PRISMA-DTA Checklist Item	Reported on page #
Meta-analysis	D2	Report the statistical methods used for meta-analyses, if performed.	NA
Additional analyses	16	Describe methods of additional analyses (e.g., sensitivity or subgroup analyses, meta-regression), if done, indicating which were pre-specified.	11
**RESULTS**			
Study selection	17	Provide numbers of studies screened, assessed for eligibility, included in the review (and included in meta-analysis, if applicable) with reasons for exclusions at each stage, ideally with a flow diagram.	13, Figure 1
Study characteristics	18	For each included study provide citations and present key characteristics including: a) participant characteristics (presentation, prior testing), b) clinical setting, c) study design, d) target condition definition, e) index test, f) reference standard, g) sample size, h) funding sources	13
Risk of bias and applicability	19	Present evaluation of risk of bias and concerns regarding applicability for each study.	18-20, Table 3
Results of individual studies	20	For each analysis in each study (e.g. unique combination of index test, reference standard, and positivity threshold) report 2x2 data (TP, FP, FN, TN) with estimates of diagnostic accuracy and confidence intervals, ideally with a forest or receiver operator characteristic (ROC) plot.	NA
Synthesis of results	21	Describe test accuracy, including variability; if meta-analysis was done, include results and confidence intervals.	17-18
Additional analysis	23	Give results of additional analyses, if done (e.g., sensitivity or subgroup analyses, meta-regression; analysis of index test: failure rates, proportion of inconclusive results, adverse events).	NA
**DISCUSSION**			
Summary of evidence	24	Summarize the main findings including the strength of evidence.	21-23
Limitations	25	Discuss limitations from included studies (e.g. risk of bias and concerns regarding applicability) and from the review process (e.g. incomplete retrieval of identified research).	22
Conclusions	26	Provide a general interpretation of the results in the context of other evidence. Discuss implications for future research and clinical practice (e.g. the intended use and clinical role of the index test).	23
**FUNDING**			
Funding	27	For the systematic review, describe the sources of funding and other support and the role of the funders.	24

Adapted From: McInnes MDF, Moher D, Thombs BD, McGrath TA, Bossuyt PM, The PRISMA-DTA Group (2018). Preferred Reporting Items for a Systematic Review and Meta-analysis of Diagnostic Test Accuracy Studies: The PRISMA-DTA Statement. JAMA. 2018 Jan 23;319(4):388-396. doi: 10.1001/jama.2017.19163.

For more information, visit: www.prisma-statement.org.

For peer review only - http://bmjopen.bmj.com/site/about/guidelines.xhtml

Synthesis Without Meta-analysis (SWiM) reporting items

The citation for the Synthesis Without Meta-analysis explanation and elaboration article is: Campbell M, McKenzie JE, Sowden A, Katikireddi SV, Brennan SE, Ellis S, Hartmann-Boyce J, Ryan R, Shepperd S, Thomas J, Welch V, Thomson H. Synthesis without meta-analysis (SWiM) in systematic reviews: reporting guideline BMJ 2020;368:l6890 http://dx.doi.org/10.1136/bmj.l6890

**SWiM is intended to complement and be used as an extension to PRISMA**			
**SWiM reporting item**	**Item description**	**Page in manuscript where item is reported**	**Other***
Methods			
**1** Grouping studies for synthesis	1a) Provide a description of, and rationale for, the groups used in the synthesis (e.g., groupings of populations, interventions, outcomes, study design)	11	Published protocol
	1b) Detail and provide rationale for any changes made subsequent to the protocol in the groups used in the synthesis	11	
**2** Describe the standardised metric and transformation methods used	Describe the standardised metric for each outcome. Explain why the metric(s) was chosen, and describe any methods used to transform the intervention effects, as reported in the study, to the standardised metric, citing any methodological guidance consulted	11	PROSPERO(CRD42021237249.)
**3** Describe the synthesis methods	Describe and justify the methods used to synthesise the effects for each outcome when it was not possible to undertake a meta-analysis of effect estimates	11	
**4** Criteria used to prioritise results for summary and synthesis	Where applicable, provide the criteria used, with supporting justification, to select the particular studies, or a particular study, for the main synthesis or to draw conclusions from the synthesis (e.g., based on study design, risk of bias assessments, directness in relation to the review question)	NA – narrative synthesis undertaken. No prioritising was undertaken of the results for	PROSPERO(CRD42021237249.)

For peer review only - http://bmjopen.bmj.com/site/about/guidelines.xhtml

Synthesis Without Meta-analysis (SWiM) reporting items

		summary and synthesis	
**SWiM reporting item**	**Item description**	**Page in manuscript where item is reported**	**Other***
**5** Investigation of heterogeneity in reported effects	State the method(s) used to examine heterogeneity in reported effects when it was not possible to undertake a meta-analysis of effect estimates and its extensions to investigate heterogeneity	NA – narrative synthesis undertaken. Meta-analysis not performed due to the broad nature of included studies	
**6** Certainty of evidence	Describe the methods used to assess certainty of the synthesis findings	NA – narrative synthesis undertaken	
**7** Data presentation methods	Describe the graphical and tabular methods used to present the effects (e.g., tables, forest plots, harvest plots).<br><br>Specify key study characteristics (e.g., study design, risk of bias) used to order the studies, in the text and any tables or graphs, clearly referencing the studies included	11	PROSPERO(CRD42021237249.)
Results			
**8** Reporting results	For each comparison and outcome, provide a description of the synthesised findings, and the certainty of the findings. Describe the result in language that is consistent with the question the synthesis addresses, and indicate which studies contribute to the synthesis	13 - 20	
Discussion			

For peer review only - http://bmjopen.bmj.com/site/about/guidelines.xhtml

Synthesis Without Meta-analysis (SWiM) reporting items

**9** Limitations of the synthesis	Report the limitations of the synthesis methods used and/or the groupings used in the synthesis, and how these affect the conclusions that can be drawn in relation to the original review question	22	

PRISMA=Preferred Reporting Items for Systematic Reviews and Meta-Analyses.
*If the information is not provided in the systematic review, give details of where this information is available (e.g., protocol, other published papers (provide citation details), or website (provide the URL)).

For peer review only

For peer review only - http://bmjopen.bmj.com/site/about/guidelines.xhtml
